# Equity in healthcare for coronary heart disease, Wales (UK) 2004–2010: A population-based electronic cohort study

**DOI:** 10.1371/journal.pone.0172618

**Published:** 2017-03-16

**Authors:** William King, Arron Lacey, James White, Daniel Farewell, Frank Dunstan, David Fone

**Affiliations:** 1 Aneurin Bevan Gwent Local Public Health Team, Public Health Wales, Newport, Wales, United Kingdom; 2 College of Medicine, Swansea University, Swansea, Wales, United Kingdom; 3 Centre for the Development and Evaluation of Complex Public Health Interventions and South East Wales Trials Unit, Cardiff University, Wales, United Kingdom; 4 Division of Population Medicine, Cardiff University, Cardiff, Wales, United Kingdom; University of Bologna, ITALY

## Abstract

**Background:**

Despite substantial falls in coronary heart disease (CHD) mortality in the United Kingdom (UK), marked socioeconomic inequalities in CHD risk factors and CHD mortality persist. We investigated whether inequity in CHD healthcare in Wales (UK) could contribute to the observed social gradient in CHD mortality.

**Methods and findings:**

Linking data from primary and secondary care we constructed an electronic cohort of individuals (n = 1199342) with six year follow-up, 2004–2010. We identified indications for recommended CHD interventions, measured time to their delivery, and estimated risk of receiving the interventions for each of five ordered deprivation groups using a time-to-event approach with Cox regression frailty models. Interventions in primary and secondary prevention included risk-factor measurement, smoking management, statins and antihypertensive therapy, and in established CHD included medication and revascularization. For primary prevention, five of the 11 models favoured the more deprived and one favoured the less deprived. For medication in secondary prevention and established CHD, one of the 15 models favoured the more deprived and one the less deprived. In relation to revascularization, six of the 12 models favoured the less deprived and none favoured the more deprived–this evidence of inequity exemplified by a hazard ratio for revascularization in stable angina of 0.79 (95% confidence interval 0.68, 0.92). The main study limitation is the possibility of under-ascertainment or misclassification of clinical indications and treatment from variability in coding.

**Conclusions:**

Primary care components of CHD healthcare were equitably delivered. Evidence of inequity was found for revascularization procedures, although this inequity is likely to have only a modest effect on social gradients in CHD mortality. Policymakers should focus on reducing inequalities in CHD risk factors, particularly smoking, as these, rather than inequity in healthcare, are likely to be key drivers of inequalities in CHD mortality.

## Introduction

Coronary heart disease (CHD) mortality rates have declined rapidly in recent decades in most middle- to high-income countries **[1,2,3].** However a steep social gradient in age-adjusted CHD mortality persists **[1,4].** In the UK the rate ratio for premature CHD death in men was 1.84 comparing residents in the most and least deprived quintiles in 2008 **[1]** and the decline in CHD mortality in England (1982–2006) was faster in the least deprived areas **[5].** Marked socioeconomic inequalities in major risk factors for CHD have been found in the UK **[6,7, 8]** but it is not clear whether these inequalities fully explain the mortality gradient, as inequity (inequality to the disadvantage of more deprived groups) in provision of or access to healthcare might contribute to the gradient.

Modelling studies of UK populations have estimated that the decline in CHD mortality has been largely due to population-level reduction in risk factors, particularly rates of smoking and levels of blood pressure and cholesterol. [**9–12].** The IMPACT studies, which estimated the proportions of the fall in CHD mortality attributable to changes in risk factors or treatments (effectiveness and provision) suggest that in England and Wales, between 1981 and 2000, 58% of the fall in CHD mortality could be attributed to population-level reduction in major risk factors and 42% to treatments [**9**]. An IMPACT study of the period 2000–2007 in England, during which CHD mortality fell by 36%, estimated that improved uptake of treatments accounted for approximately 50% of the fall [**13**].

A number of UK-based ecological studies have reported inequity in use of antihypertensive medication [**14–16**] and lipid-lowering medication [**17,18**] using analysis of practice-level data. A large individual-level UK study of secondary prevention of CHD found no evidence of inequity, and some findings suggested that more deprived groups were more likely to receive treatment [**19**]. UK-based individual-level studies of the management of diabetes found no evidence of inequity in the prescribing of antihypertensive medication and lipid-lowering therapy [**20–22**]. Other studies have reported clear evidence of inequity in the use of percutaneous coronary intervention (PCI) and coronary artery bypass grafting (CABG) in the UK [**23–29**].

A number of studies have examined different components of the CHD healthcare pathway [**13,14,19**]. We are not aware of any that examined whether there are inequities across the entire CHD pathway, from risk assessment, to primary and secondary prevention, medication in established CHD, and revascularization procedures. This is an important gap in the literature as the existing studies of different parts of the CHD pathway do not permit strong inferences to be made about the cumulative effect of inequity in one part of the pathway on inequities that become apparent at a later stage, and do not investigate inequity as a systematic, whole-pathway phenomenon.

We examined socioeconomic inequalities across a recommended CHD healthcare pathway in a population-level record-linked cohort study based on primary care, secondary care and demographic and mortality data from over one million adults, 2004–2010.

The study period followed introduction of the National Service Frameworks (NSF) for Coronary Heart Disease (introduced in 2000 in England and in 2001 in Wales) **[30, 31]** which set standards for all aspects of management of CHD, and the period coincided with the Quality Outcomes Framework (QOF) **[32]** introduced in 2004 to improve primary care including CHD healthcare in the UK.

## Methods

All analyses were performed within the Secure Anonymized Information Linkage databank (SAIL) at Swansea University **[33,34]**. This system allows researchers to link anonymised data, including routine primary care, hospital activity, mortality and demographic data in a secure environment. SAIL implements rigorous information governance arrangements involving systematic data anonymisation, access limitations and disclosure controls. Permission to undertake the analysis was obtained from the Information Governance Review Panel at SAIL in line with the Collaborative Review System (project reference number 0156).

### Datasets

We defined an electronic cohort of individuals aged 20 or over, resident in Wales and registered with SAIL-submitting general practices between 1 January 2004 and 31 December 2010. Routine data from the Welsh Demographic Service, Office of National Statistics (ONS) mortality files (ICD10 codes for cause of death), Patient Episode Data for Wales (PEDW) hospital admission data (ICD 10 and Office of Population and Censuses (OPCS) codes for CHD-related hospital episodes and procedures), and primary care data (Read codes for diagnosis, investigation and treatment of CHD and for the prescribing of antihypertensive, lipid-lowering and anti-platelet therapy) was extracted to form a linked dataset (for codes see S1 File).

### Assessment of socioeconomic inequalities

The Welsh Index of Multiple Deprivation (WIMD) (2008) for the area of residence of the individual assessed at the Lower Super Output Area (LSOA) was used as a measure of socioeconomic deprivation. An LSOA is a unit of small-area geography used in the UK with a mean population of 1500. WIMD 2008 at LSOA level is based on residents’ income, employment status, education, housing, health and geographical access to services [**35].**

### Pathway of CHD care

**Fig 1:**
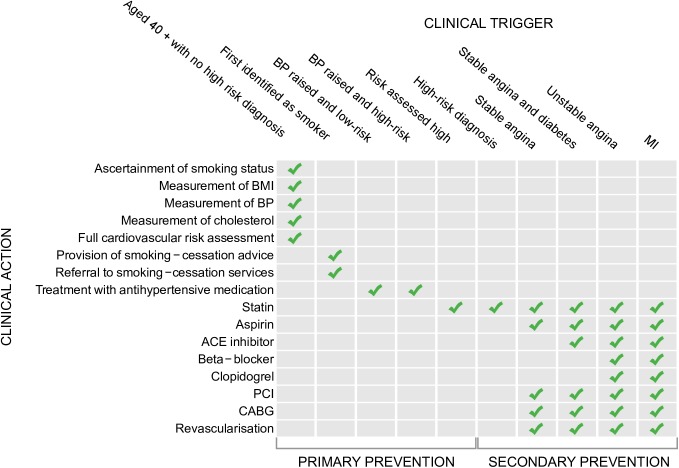
Clinical triggers and clinical actions investigated in the healthcare pathway for coronary heart disease. Top row shows the clinical triggers in the healthcare pathway. The left-hand side shows the clinical actions identified in the pathway of care. Where boxes corresponding to a clinical trigger and clinical action are ticked, equity in the provision of care for that combination of clinical trigger and clinical action was investigated.

The pathway consisted of a sequence of evidence-based interventions recommended in NSFs **[30, 31]** and National Institute for Health and Clinical Excellence (NICE) guidelines **[36–40].** These guidelines include CHD risk-assessment, primary prevention, secondary prevention, and medical and surgical treatment of established disease. The pathway of care investigated is shown in Fig 1.

Because of the complexity of the clinical algorithms for treating hypertension, the analysis of this area of the pathway was simplified by considering initiation of treatment as the prescription of any antihypertensive medication.

We identified ‘clinical trigger states’, defined as arising when an individual had an indication for an intervention according to NICE guidelines. Fig 1 shows the points at which clinical triggers (along the top of the figure) would be expected to prompt clinical actions (left hand side of the figure). In our comparisons for revascularization procedures we included a composite measure that included both PCI and CABG in order to avoid the possibility that increased use of either procedure might lead to a reduced need for the other procedure.

#### Covariates

For each clinical trigger identified in an individual, we determined covariates at the first appearance of the clinical trigger. Covariates available included demographic factors (age, sex, WIMD 2008); risk-factors (systolic blood pressure (SBP), BMI, smoking status, cholesterol: HDL ratio); co-morbidities based on the Charlson co-morbidity index [**41**] collapsed to a binary variable because some components (CHD, cerebrovascular disease and diabetes) were already considered as covariates; and the Framingham non-laboratory risk assessment score (comprising sex, age, SBP, BMI, smoking status, reported diabetic status, and current treatment for hypertension) [**42**]. To take account of an individual’s previous progress in the pathway we included covariates for the timing of successive indications for the same intervention (for example high cardiovascular disease risk and subsequent angina both being indications for lipid-lowering therapy). Thus our models were able to represent an individual’s cumulative experience in the pathway.

### Statistical methods

We used a Cox model with random effects to examine associations with time-to-healthcare provision, measuring from the initiation of the clinical trigger state to the delivery of the indicated clinical action. We adjusted for important covariates, with the individual’s general practice or admitting hospital modelled as random effects, to allow for unobserved hospital or GP specific factors. Modelling was performed using the *coxme* package in R **[43]**. For each point on the pathway we selected covariates on the basis of relevance. Information on the covariates used in the models is provided in S2 File.

Absolute inequalities were examined by comparing WIMD quintile 5 (most deprived) to quintile 1 (least deprived). Deprivation quintile (Welsh Index of Multiple Deprivation 2008) of residence for the individual was included as a term in every model. We used multiple imputations with chained equations (MICE) to create five imputed datasets. We imputed all missing covariates (systolic blood pressure, BMI, cholesterol: HDL ratio, smoking status, admission specialty, and admission type), using the MICE package in R 2.13.2. **[44].** The type 1 error probability was set to 0.05 for all analyses.

We performed several sensitivity analyses. We re-ran the analyses using the Framingham 1991 risk-assessment tool **[45]** rather than the non-laboratory tool, the 2001 Townsend deprivation index **[46]** rather than WIMD, and 20 imputations rather than five in the chained equations for multiple imputation. We also repeated models using a slope index of inequality across all quintiles, instead of looking at the HR between the most deprived and least deprived quintiles.

## Results

The initial cohort totalled 1201399 but after exclusion of individuals with clearly incorrectly coded date of birth (202), absent coding for gender (7) or with discontinuous registration with SAIL (1848) the cohort was reduced to 1199342. The primary care data available for our study was available only from SAIL-submitting practices and covered approximately 40% of the population of Wales, with a disproportionately high level of coverage in south west Wales. There is no available evidence that these practices were unrepresentative, and the distribution of urban and rural residency of the population resembled that of Wales as a whole. Comparing our data with ONS mid-year data for the whole of Wales, small differences were seen in age distribution, our cohort having 1.2% fewer in the proportion aged over 40.

The clinical triggers and related clinical actions are summarized in Table 1.

**Table 1:** Numbers of clinical triggers and associated clinical actions at different positions in the pathway of care for coronary heart disease.

Pathway position	Clinical trigger	Clinical action	Number of clinical triggers	Number of clinical actions
1	Aged 40+ with no high risk diagnosis	Ascertainment of smoking status	122486	72291
2	Aged 40+ with no high risk diagnosis	Measurement of BMI	122486	46235
3	Aged 40+ with no high risk diagnosis	Measurement of BP	122486	64312
4	Aged 40+ with no high risk diagnosis	Measurement of cholesterol	122486	28652
5	Aged 40+ with no high risk diagnosis	Full cardiovascular risk assessment	122486	84969
6	First identified as smoker	Referral to smoking-cessation services	55161	2514
7	First identified as smoker	Provision of smoking-cessation advice	55161	45926
8	BP raised and low-risk	Treatment with antihypertensive medication	13814	9899
9	BP raised and high-risk	Treatment with antihypertensive medication	106079	75797
10	Risk assessed high	Statin	105301	20661
11	High-risk diagnosis	Statin	34387	19389
12	Stable angina	Statin	11104	4660
13	Stable angina and diabetes	Statin	2457	968
14	Unstable angina	Statin	4462	2178
15	MI	Statin	10442	5372
16	Stable angina	Aspirin	9433	3923
17	Stable angina and diabetes	Aspirin	2736	919
18	Unstable angina	Aspirin	4172	2041
19	Stable angina	Statin	11104	4660
20	Stable angina and diabetes	ACE inhibitor	3361	1092
21	Unstable angina	ACE inhibitor	5287	1967
22	MI	ACE inhibitor	10595	5270
23	Unstable angina	Beta-blocker	10405	285
24	MI	Beta-blocker	16639	363
25	Unstable angina	Clopidogrel	13907	5783
26	MI	Clopidogrel	20467	10132
27	Stable angina	PCI	18934	1172
28	Stable angina and diabetes	PCI	8956	300
29	Unstable angina	PCI	13907	2130
30	MI	PCI	20467	5118
31	Stable angina	CABG	18934	1150
32	Stable angina and diabetes	CABG	8956	385
33	Unstable angina	CABG	13907	1155
34	MI	CABG	20467	1645
35	Stable angina	Revascularisation	18934	2298
36	Stable angina and diabetes	Revascularisation	8956	676
37	Unstable angina	Revascularisation	13907	3230
38	MI	Revascularisation	20467	6649

**Fig 2:**
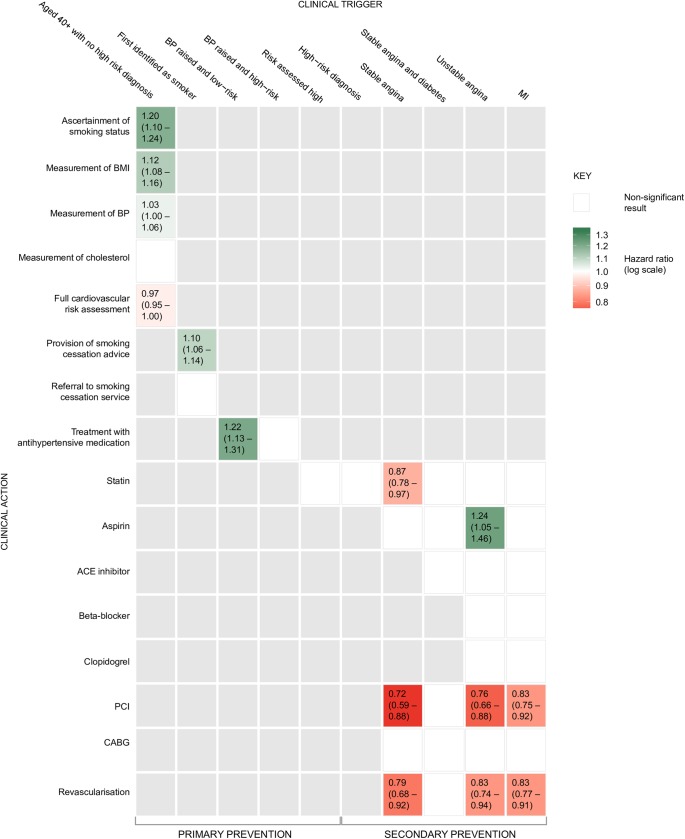
Hazard ratios (HRs) and 95% confidence intervals (CIs) for the association between absolute socioeconomic inequalities and provision of healthcare for or coronary heart disease. Where the association is not statistically significant at the p<0.05 level the box is coloured white. For statistically significant results, the box is coloured according to the magnitude of effect on a logarithmic scale; green shading indicates that the most deprived quintile of the population was more likely to receive the clinical action; red shading indicates that the least deprived quintile of the population was more likely to receive the clinical action.

Fig 2 shows the hazard ratio for the most deprived compared with the least deprived quintile (with 95% confidence intervals) for socioeconomic inequalities across the pathway of CHD healthcare.

### Management of risk-factors

The clinical trigger, based on NSF and NICE guidance, for CHD risk-factor assessment was that the individual was aged 40 or over and was not previously recognized as being at high CHD risk. Three of the five comparisons showed components of CHD risk-assessment that favoured the most deprived quintile: ascertainment of smoking status, HR 1.20 (95% CI 1.17–1.24), BMI ascertainment, HR 1.12 (95% CI 1.08–1.16), measurement of BP, HR 1.03 (95% CI 1.00–1.06). The recording of a full risk-profile favoured the least deprived quintile, HR 0.97 (95% CI 0.95–1.00).

Provision of smoking-cessation advice favoured the most deprived group, HR 1.1 (95% CI 1.06–1.14).

Use of antihypertensive medication in individuals with raised systolic blood pressure (three readings above 160 mm Hg **[38]**) but otherwise at low risk, favoured the most deprived group, HR 1.22 (95% CI 1.13–1.31).

### Medication in established disease

Use of statins in individuals with stable angina favoured the least deprived, HR 0.87 (95% CI 0.78–0.97). Use of aspirin in individuals with unstable angina favoured the most deprived, HR 1.24 (95% CI 1.05–1.46). The other 13 comparisons showed no statistically significant differences.

### Revascularization

Of the 12 comparisons made in relation to revascularization procedures six favoured the least deprived, and six showed no significant difference although favouring at a non-significant level the least deprived. In individuals with stable angina the HR for PCI was 0.72 (95% CI 0.59–0.88) and for revascularization (combined) 0.79 (95% CI 0.68–0.92). In unstable angina the HR for PCI was 0.76 (95% CI 0.66–0.88) and for revascularization (combined) 0.83 (95% CI 0.74–0.94). In myocardial infraction (MI) the HR for PCI was 0.83 (95% CI 0.75–0.92) and for revascularization (combined) 0.83 (95% CI 0.77–0.91).

### Sensitivity analyses

Sensitivity analyses, using the Framingham 1991 risk-assessment tool rather than the non-laboratory tool, the 2001 Townsend deprivation quintiles rather than WIMD (2005), 20 imputations in the chained equations for multiple imputations, and using a slope-index-of-equality (instead of looking at the HR for the most deprived compared to least deprived deprivation quintile) all had little effect on the overall pattern of our findings.

## Discussion

### Main findings

For the healthcare pathway (excluding the composite revascularisation outcome), we found six points in the pathway where the most deprived quintile were more likely to receive the clinical action in question and five points where the least deprived were more likely to receive the clinical action; at 23 points there was no significant difference. Our interpretation of these findings was that, in a population-level analysis of the entire CHD healthcare pathway, there was no evidence of systematic inequity in utilisation of healthcare adjusted for need.

The study was not designed to identify inequity at specific points in the pathway, and, with the problem of multiple comparison in mind, interpretation of findings at specific stages in the pathway needs to be undertaken with caution. We did identify evidence that pattern of the inequalities related to the stages of the pathway. For primary prevention, significant inequalities favouring the most deprived quintile were found in five of the 11 interventions. Of the 13 interventions for medication in secondary prevention and in established CHD one inequality favoured the least deprived quintile and one the most deprived. There was evidence of inequality favouring the least deprived quintile in six of the 12 interventions relating to revascularization.

### Comparison with other studies

An individual-level study comprehensively examined prescribing in secondary prevention of CHD in the UK between 1999 and 2007 and detected no evidence of inequity, its findings suggesting greater prescribing in the most than in the least deprived groups **[19]**. An IMPACT modelling study to examine the relationship between socioeconomic status and the factors that explain the declining rates of CHD mortality in the UK between 2000 and 2007 found no evidence of inequity in the delivery of a wide range of interventions **[13].** Our findings therefore broadly concur with those of these studies and a number of other individual-level studies [**20–22]** although they conflict with those of several ecological studies [**14–18**] and studies of UK populations during the 1990’s **[47–49]** that reported evidence of inequity in primary care elements of CHD healthcare.

Our findings replicate those of a number of studies covering the same time period relating to revascularization in UK populations, in which rates of revascularization favoured the less deprived [**23–29**]. Several studies reporting inequity in revascularization suggest that individuals’ attitudes, expectations and consultation thresholds, differing according to socioeconomic status, may contribute to the inequalities observed [**28,50,51].** One qualitative study suggested that attitudes such as low expectations of treatment and fear of hospitals were more likely to be found in more deprived groups and formed barriers to referral for angiography and subsequent revascularization [**52**].

### Implications for policy

We found that the primary care components of CHD healthcare were delivered equitably in the population of Wales during the study period 2004–2010. Despite this finding and the results of comparable studies **[13, 19–22]**, there is evidence that the fall in CHD mortality in the UK has been faster in more affluent groups and that relative inequality in CHD mortality has increased **[13,53]**. Adverse effects of increasing rates of diabetes and obesity are expected to offset improvements in population risk-profiles [**9–12**] and, because of higher rates of these conditions in more deprived groups, to increase relative inequality. These studies suggest that even if CHD healthcare is equitably delivered, inequalities in CHD outcomes are likely to persist, and relative inequalities to increase, unless inequalities in major risk-factors such as tobacco use are addressed.

We found clear evidence of inequity in revascularization procedures. Marked increases in the resources for revascularization, and in the volume of procedures carried out, were observed in the UK during our study period. Between 2000 and 2012 the rates of PCI (all types) in Wales increased from 550 per million population to 1363 per million, and between 2004 and 2008 increased from 900 to 1150 per million population. **[54].** Our findings suggest that this increase was not applied equitably. It would be valuable to examine reasons for this, in particular deprivation-related attitudes (such as low expectations of healthcare and fear of hospitals) that limit demand, and the distribution of co-morbidities across the social gradient. Comorbidities, potentially acting as contraindications to revascularization procedures, may explain part of the observed inequality, and in our study the evidence of inequity weakened when the model was adjusted for comorbidities.

Evidence from modelling studies helps to clarify the potential effect on CHD mortality of the observed inequity in revascularization. In England and Wales between 1981 and 2000, while 42% of the decline in CHD mortality was due to medical and surgical intervention, only 3.8% of deaths prevented or postponed (DPP) were due to CABG or angioplasty **[9**]. This estimate is similar to that reported in a comparable USA study **[55**]. In a study of USA cardiac patients (1980–2000) it was estimated that less than 6% of total life-years gained were attributable to revascularization procedures **[56]**. In a study of declining CHD mortality in New Zealand 1983–1993 **[57]** DPP by revascularization procedures were estimated to contribute 5% of the total CHD mortality reduction. A study using IMPACT modelling to explain the decline in CHD mortality in Northern Ireland between 1987 and 2007, calculated that CABG or angioplasty in acute MI or unstable angina accounted for less than 1% of DPP [**11**]. We assess from such estimates that the degree of inequity that we observed in revascularization procedures would make only a small contribution to the steep social gradient of CHD mortality (hazard ratio 1.72 in our adjusted model). Further work to monitor inequalities in revascularization and to quantify their effects on mortality, would be valuable to policy-makers.

### Strengths of the study

We based our study on data from a large number of individuals (more than one million) and identified a large number of clinical triggers for each of which we determined the time to clinical action. In contrast to previous studies that had examined different parts of CHD risk-factor assessment, management and treatments, we were able to examine inequalities across an entire recommended CHD pathway at the individual level. We were able to use a hierarchical structure (individual/practice/hospital) in our modelling and this allowed us to take account of supply-side factors as random variables. By using a time-to-event approach we eliminated in our analysis the use of an arbitrary standard for acceptable time-intervals between clinical trigger and action.

We used a set of algorithms for identifying, collecting and classifying relevant information on clinical triggers from a large and unrefined data source. Information on the length of time for which a particular clinical action was indicated and the number of different previous indications that had arisen for that action, enabled us to adjust for elements in an individual’s history as potential confounders in models.

This methodology may be applicable to studies of pathways of care for diseases other than CHD.

### Limitations

The main study limitation is that inaccuracies and variability in the use of Read and ICD codes in medical records are known to occur **[58,59].** Under-ascertainment or misclassification of clinical indications and interventions would tend to bias the results towards null, potentially reducing the power of our study to identify genuine inequity.

Prescribing data relating to inpatient treatment of acute coronary syndromes was not available in our routine data and we therefore had information on drug treatments for individuals only after they had left hospital. We did not to include anti-anginal therapy in the pathway as it is not considered directly to affect CHD mortality, and because our data would not necessarily allow us to distinguish whether some types of medication, including calcium channel blockers and beta blockers, were used in an individual to treat angina or hypertension.

Our study examined a health service in which healthcare is free at the point of delivery and there is no charge for prescriptions. The system of healthcare in the UK, in which interventions such as QOF can operate, contrasts with less integrated systems such as those in the USA, and further work using a similar approach in such health systems without might be revealing.

### Conclusions and recommendations

Primary care components of CHD healthcare were equitably delivered in the population of Wales between 2004 and 2011. Clear evidence of inequity was found in relation to revascularization procedures.

Organisations and policymakers should focus on the clear social gradients in risk factors as it is these, rather than inequity in healthcare, that are the key drivers of social gradients in CHD mortality. They should address the increasing rates of obesity and diabetes that are offsetting the benefits of recent reductions in other major CHD risk factors such as smoking.

The time-to-event methodology of this study has been shown to be an effective way of examining evidence of equity in utilization of healthcare and could be similarly used in studies of other disease areas.

## Supporting information

S1 File Clinical codes. Clinical codes used to define clinical conditions from routine data.
(PDF)

S2 File Covariates and outputs from Cox models. (PDF)

## Abbreviations

BMI: body mass index
CABG: coronary artery bypass graft
CHD: coronary heart disease
CI: confidence interval
DPP: deaths prevented or postponed
HDL: high-density lipoprotein
HR: hazard ratio
LSOA: lower super output area
MI: myocardial infarction
MICE: multiple imputations with chained equations
NICE: National Institute for Clinical Excellence (before 1^st^ April 2005), National Institute for Health and Clinical Excellence (1^st^ April 2005-31^st^ March 2013), National Institute for Health and Care excellence (from 1^st^ April 2013).
NSF: National Service Framework
ONS: Office for National Statistics
OPCS: Office of Population Censuses and Surveys
PCI: percutaneous coronary intervention
PEDW: Patient Episode Data for Wales
PSALF: Project specific anonymised linking field
QOF: Quality and Outcomes Framework
SAIL: Secure Anonymized Data Linkage
SBP: systolic blood pressure
UK: United Kingdom
WIMD: Welsh Index of Multiple Deprivation

## References

[1] McCartney D, Scarborough P, Webster P, Rayner M. Trends in social inequalities for premature coronary heart disease mortality in Great Britain, 1994–2008: a time trend ecological study. BMJ Open. 2012 1 1;2(3):e000737 10.1136/bmjopen-2011-000737 22710128 PMC3378944

[2] Wilmot KA, O’Flaherty M, Capewell S, Ford ES, Vaccarino V. Coronary Heart Disease Mortality Declines in the United States From 1979 Through 2011 Evidence for Stagnation in Young Adults, Especially Women. Circulation. 2015 9 15;132(11):997–1002. 10.1161/CIRCULATIONAHA.115.015293 26302759 PMC4828724

[3] Ford ES, Ajani UA, Croft JB, Critchley JA, Labarthe DR, Kottke TE, et al Explaining the decrease in US deaths from coronary disease, 1980–2000. N Engl J Med. 2007 6 7;356(23):2388–98. 10.1056/NEJMsa053935 17554120

[4] Leyland AH. Increasing inequalities in premature mortality in Great Britain. J Epidemiol Community Health. 2004 4 1;58(4):296–302. 10.1136/jech.2003.007278 15026442 PMC1732731

[5] Bajekal M, Scholes S, O’Flaherty M, Raine R, Norman P, Capewell S. Unequal trends in coronary heart disease mortality by socioeconomic circumstances, England 1982–2006: an analytical study. PLoS One. 2013 3 20;8(3):e59608 10.1371/journal.pone.0059608 23527228 PMC3603902

[6] Jefferis BJ, Power C, Graham H, Manor O. Changing social gradients in cigarette smoking and cessation over two decades of adult follow-up in a British birth cohort. J Public Health (Oxf). 2004 3 1;26(1):13–8. 15044567 10.1093/pubmed/fdh110

[7] Lyratzopoulos G, Heller RF, McElduff P, Hanily M, Lewis P. Deprivation and trends in blood pressure, cholesterol, body mass index and smoking among participants of a UK primary care-based cardiovascular risk factor screening programme: both narrowing and widening in cardiovascular risk factor inequalities. Heart. 2006 9 1;92(9):1198–206. 10.1136/hrt.2005.081042 16467458 PMC1861148

[8] Simpson CR, Hippisley-Cox J, Sheikh A. Trends in the epidemiology of smoking recorded in UK general practice. Br J Gen Pract. 2010 3 1;60(572):e121–7. 10.3399/bjgp10X483544 20202355 PMC2828860

[9] Unal B, Critchley JA, Capewell S. Explaining the decline in coronary heart disease mortality in England and Wales between 1981 and 2000. Circulation. 2004 3 9;109(9):1101–7. 10.1161/01.CIR.0000118498.35499.B2 14993137

[10] O'Flaherty M, Buchan I, Capewell S. Contributions of treatment and lifestyle to declining CVD mortality: why have CVD mortality rates declined so much since the 1960s? Heart. 2013 2 1;99(3):159–62. 10.1136/heartjnl-2012-302300 22962283

[11] Hughes J, Kee F, O’Flaherty M, Critchley J, Cupples M, Capewell S, et al Modelling coronary heart disease mortality in Northern Ireland between 1987 and 2007: broader lessons for prevention. Eur J Prev Cardiol. 2013 4 1;20(2):310–21. 10.1177/2047487312441725 22403395

[12] Hotchkiss JW, Davies CA, Dundas R, Hawkins N, Jhund PS, Scholes S, et al Explaining trends in Scottish coronary heart disease mortality between 2000 and 2010 using IMPACTSEC model: retrospective analysis using routine data. BMJ 2014;348:g1088 10.1136/bmj.g1088 24503058 PMC3915926

[13] Bajekal M, Scholes S, Love H, Hawkins N, O'Flaherty M, Raine R, et al Analysing recent socioeconomic trends in coronary heart disease mortality in England, 2000–2007: a population modelling study. PLoS Med. 2012 6 12;9(6):e1001237 10.1371/journal.pmed.1001237 22719232 PMC3373639

[14] Saxena S, Car J, Eldred D, Soljak M, Majeed A. Practice size, caseload, deprivation and quality of care of patients with coronary heart disease, hypertension and stroke in primary care: national cross-sectional study. BMC Health Serv Res. 2007 6 27;7(1) 10.1186/1472-6963-7-96 PMC1919365 17597518

[15] Ashworth M, Medina J, Morgan M. Effect of social deprivation on blood pressure monitoring and control in England: a survey of data from the quality and outcomes framework. BMJ 2008 10 29;337:a2030 10.1136/bmj.a2030 18957697 PMC2590907

[16] Ward PR, Noyce PR, St Leger AS. Exploring the equity of GP practice prescribing rates for selected coronary heart disease drugs: a multiple regression analysis with proxies of healthcare need. Int J Equity Health. 2005 2 8;4(1):3 10.1186/1475-9276-4-3 15701165 PMC548940

[17] Ward PR, Noyce PR, St Leger AS. Are GP practice prescribing rates for coronary heart disease drugs equitable? A cross sectional analysis in four primary care trusts in England. J Epidemiol Community Health. 2004 2 1;58(2):89–96. 10.1136/jech.58.2.89 14729882 PMC1732682

[18] McLean G, Sutton M, Guthrie B. Deprivation and quality of primary care services: evidence for persistence of the inverse care law from the UK Quality and Outcomes Framework. J Epidemiol Community Health. 2006 11 1;60(11):917–22. 10.1136/jech.2005.044628 17053278 PMC2465488

[19] Hawkins NM, Scholes S, Bajekal M, Love H, O’Flaherty M, Raine R, et al The UK National Health Service Delivering Equitable Treatment Across the Spectrum of Coronary Disease. Circ Cardiovasc Qual Outcomes. 2013 3 1;6(2):208–16. 10.1161/CIRCOUTCOMES.111.000058 23481523

[20] McGovern MP, Williams DJ, Hannaford PC, Taylor MW, Lefevre KE, Boroujerdi MA, et al Introduction of a new incentive and target-based contract for family physicians in the UK: good for older patients with diabetes but less good for women? Diabet Med. 2008 9 1;25(9):1083–9. 10.1111/j.1464-5491.2008.02544.x 18937676

[21] Hippisley-Cox J O'Hanlon S, Coupland C. Association of deprivation, ethnicity, and sex with quality indicators for diabetes: population based survey of 53 000 patients in primary care. BMJ 2004 11 25;329(7477):1267–9. 10.1136/bmj.38279.588125.7C 15548561 PMC534442

[22] Hamilton FL, Bottle A, Vamos EP, Curcin V, Molokhia M, Majeed A, et al Impact of a pay-for-performance incentive scheme on age, sex, and socioeconomic disparities in diabetes management in UK primary care. J Ambul Care Manage. 2010 10 1;33(4):336–49. 10.1097/JAC.0b013e3181f68f1d 20838113

[23] Cosh H. Coronary heart disease in Wales: an ecological study of socioeconomic variations in mortality and hospital treatment, 1992–2006 [master’s thesis]. [Birmingham (UK)]: University of Birmingham; 2008

[24] Lester N. Is there equity of access to coronary angiography and revascularisation according to socioeconomic deprivation for people in Wales? A cross-sectional ecological study [master’s thesis]. [Cardiff]: University of Cardiff; 2004.

[25] Gatrell A, Lancaster G, Chapple A, Horsley S, Smith M. Variations in use of tertiary cardiac services in part of North-West England. Health Place. 2002 9 30;8(3):147–53. 12135638 10.1016/s1353-8292(01)00044-2

[26] Hippisley-Cox J, Pringle M. Inequalities in access to coronary angiography and revascularisation: the association of deprivation and location of primary care services. Br J Gen Pract. 2000 6 1;50(455):449–54. 10962781 PMC1313721

[27] Manson-Siddle CJ, Robinson MB. Super Profile analysis of socioeconomic variations in coronary investigation and revascularisation rates. J Epidemiol Community Health. 1998 8 1;52(8):507–12. 9876362 10.1136/jech.52.8.507 PMC1756742

[28] Manson-Siddle CJ, Robinson MB. Does increased investment in coronary angiography and revascularisation reduce socioeconomic inequalities in utilisation? J Epidemiol Community Health. 1999 9 1;53(9):572–7. 10562882 10.1136/jech.53.9.572 PMC1756968

[29] Ben-Shlomo Y, Chaturvedi N. Assessing equity in access to health care provision in the UK: does where you live affect your chances of getting a coronary artery bypass graft? J Epidemiol Community Health. 1995 4 1;49(2):200–4. 7798051 10.1136/jech.49.2.200 PMC1060108

[30] Department of Health. National Service Framework for Coronary Heart Disease. 2000 (Department of Health, London).

[31] National Service Framework for Coronary Heart Disease. 2001 (National Assembly for Wales, Cardiff)

[32] Doran T, Fullwood C, Gravelle H, Reeves D, Kontopantelis E, Hiroeh U, et al Pay-for-performance programs in family practices in the United Kingdom. N Engl J Med. 2006 7 27;355(4):375–84. 10.1056/NEJMsa055505 16870916

[33] Ford DV, Jones KH, Verplancke JP, Lyons RA, John G, Brown G, Brooks CJ, et al The SAIL Databank: building a national architecture for e-health research and evaluation. BMC Health Serv Res. 2009 9 4;9(1):157. 19732426 10.1186/1472-6963-9-157 PMC2744675

[34] Lyons RA, Jones KH, John G, Brooks CJ, Verplancke JP, Ford DV, et al The SAIL databank: linking multiple health and social care datasets. BMC Med Inform Decis Mak. 2009 1 16;9(1):1. 19149883 10.1186/1472-6947-9-3 PMC2648953

[35] Welsh index of multiple deprivation 2008: Technical report; Available: http://wales.gov.uk/topics/statistics/publications/publication-archive/wimd2008tech/?lang=en; 2009

[36] National Institute for Clinical Excellence (2001) Prophylaxis for patients who have experienced a myocardial infarction. (CGA)

[37] National Institute for Clinical Excellence (2002) Management of type 2 diabetes–management of blood pressure and blood lipids (CGH)

[38] National Institute for Health and Clinical Excellence (2004) Hypertension: management of hypertension in adults in primary care (CG18)

[39] National Institute for Health and Clinical Excellence (2006) Hypertension: management of hypertension in adults in primary care (CG34)

[40] National Institute for Health and Clinical Excellence (2008) Lipid modification: Cardiovascular risk assessment and the modification of blood lipids for the primary and secondary prevention of cardiovascular disease (CG67) 25340243

[41] Charlson ME, Pompei P, Ales KL, MacKenzie CR. A new method of classifying prognostic comorbidity in longitudinal studies: development and validation. J chronic dis. 1987 12 31;40(5):373–83. 3558716 10.1016/0021-9681(87)90171-8

[42] D'Agostino RB Sr, Vasan RS, Pencina MJ, Wolf PA, Cobain M, Massaro M et al General cardiovascular risk profile for use in primary care. Circulation. 2008 2 12: 117(6): 743–53. 10.1161/CIRCULATIONAHA.107.699579 18212285

[43] Therneau T. coxme: Mixed effects Cox models. R package version. 2012;2(3).

[44] Van Buuren S, Groothuis-Oudshoorn K. mice: Multivariate imputation by chained equations in R. J stat softw. 2011;45(3).

[45] Anderson KM, Odell PM, Wilson PW, Kannel WB. Cardiovascular disease risk profiles. Am Heart J. 1991 1 31;121(1):293–8. 1985385 10.1016/0002-8703(91)90861-b

[46] Jarman B, Townsend P, Carstairs V. Deprivation indices. BMJ. 1991 8 31;303(6801):523. 10.1136/bmj.303.6801.523-a PMC1670803 1912874

[47] Packham C, Robinson J, Morris J, Richards C, Marks P, Gray D. Statin prescribing in Nottingham general practices: a cross-sectional study. J Public Health (Oxf). 1999 3 1;21(1):60–4. 10.1093/pubmed/21.1.60 10321861

[48] Packham C, Pearson J, Robinson J, Gray D. Use of statins in general practices, 1996–8: cross sectional study. BMJ. 2000 6 10;320(7249):1583–4. 10845969 10.1136/bmj.320.7249.1583 PMC27405

[49] Bradshaw NS, Fone DL, Walker R. Equity of health care: a ward-based analysis of primary care prescribing. Pharm J.1998;261:R11.

[50] Payne N, Saul C. Variations in use of cardiology services in a health authority: comparison of coronary artery revascularisation rates with prevalence of angina and coronary mortality. BMJ. 1997 1 25;314(7076):257 9022488 10.1136/bmj.314.7076.257 PMC2125748

[51] Langham S, Basnett I, McCartney P, Normand C, Pickering J, Sheers D, Thorogood M. Addressing the inverse care law in cardiac services. J Public Health (Oxf). 2003 9 1;25(3):202–7. 10.1093/pubmed/fdg054 14575194

[52] Gardner K, Green J, Chapple A. Barriers to referral in patients with angina: qualitative study. Commentary: Generalisability and validity in qualitative research. BMJ. 1999 8 14;319(7207):418–21. 10445924 10.1136/bmj.319.7207.418 PMC28197

[53] Bajekal M, Scholes S, O'Flaherty M, Raine R, Norman P, Capewell S. Trends in coronary heart disease mortality in England by socioeconomic circumstances, 1982–2006. J Epidemiol Community Health. 2010;64:A2-. 10.1371/journal.pone.0059608 PMC3603902 23527228

[54] British Cardiovascular Intervention Society National Audit of Percutaneous Coronary Interventional Procedures Public Report (Annual Public Report January 2012-December 2012). 29 Jan 2014. British Cardiovascular Intervention Society. Available: www.bcis.org.uk/resources/PCI_Audit_Report_2012_final.pdf

[55] Doliszny KM, Luepker RV, Burke GL, Pryor DB, Blackburn H. Estimated contribution of coronary artery bypass graft surgery to the decline in coronary heart disease mortality: the Minnesota Heart Survey. J Am Coll Cardiol. 1994 7 1;24(1):95–103. 8006288 10.1016/0735-1097(94)90547-9

[56] Capewell S, Hayes DK, Ford ES, Critchley JA, Croft JB, Greenlund KJ, et al Life-years gained among US adults from modern treatments and changes in the prevalence of 6 coronary heart disease risk factors between 1980 and 2000. Am J Epidemiol 2009:kwp150. 10.1093/aje/kwp150 19541856

[57] Capewell S, Beaglehole R, Seddon M, McMurray J. Explanation for the decline in coronary heart disease mortality rates in Auckland, New Zealand, between 1982 and 1993. Circulation. 2000 9 26;102(13):1511–6. 11004141 10.1161/01.cir.102.13.1511

[58] Campbell SE, Campbell MK, Grimshaw JM, Walker AE. A systematic review of discharge coding accuracy. J Public Health. 2001 9 1;23(3):205–11. 10.1093/pubmed/23.3.205 11585193

[59] Jordan K, Porcheret M, Croft P. Quality of morbidity coding in general practice computerized medical records: a systematic review. Fam Pract. 2004 8 1;21(4):396–412. 10.1093/fampra/cmh409 15249528

