# Supplementary material for: Equity in healthcare for coronary heart disease, Wales (UK) 2004–2010: A population-based electronic cohort study
Source: PLoS One. 2017 Mar 16;12(3):e0172618. doi: 10.1371/journal.pone.0172618 (PMC5354260; doi:10.1371/journal.pone.0172618)
Supplement: S1 File — Clinical codes used to define clinical conditions from routine data. (PDF) [file pone.0172618.s001.pdf]

S1: Clinical coding

Codes used to define clinical conditions from routine data appear in the following pages.

The table is taken from the thesis on which this paper is based. Further information on the way these codes were employed in the full thesis can be obtained at: http://orca.cf.ac.uk/73460/

Table E.1: Summary of clinical codes used for defining different conditions in this thesis

Defined	System	Code	Description	Notes
Diabetes	ICD-10	E10	Insulin-dependent diabetes mellitus	
Diabetes	ICD-10	E100	Insulin-dependent diabetes mellitus: With coma	
Diabetes	ICD-10	E101	Insulin-dependent diabetes mellitus: With ketoacidosis	
Diabetes	ICD-10	E102	Insulin-dependent diabetes mellitus: With renal complications	
Diabetes	ICD-10	E103	Insulin-dependent diabetes mellitus: With ophthalmic complications	
Diabetes	ICD-10	E104	Insulin-dependent diabetes mellitus: With neurological complications	
Diabetes	ICD-10	E105	Insulin-dependent diabetes mellitus: With peripheral circulatory complications	
Diabetes	ICD-10	E106	Insulin-dependent diabetes mellitus: With other specified complications	
Diabetes	ICD-10	E107	Insulin-dependent diabetes mellitus: With multiple complications	
Diabetes	ICD-10	E108	Insulin-dependent diabetes mellitus: With unspecified complications	
Diabetes	ICD-10	E109	Insulin-dependent diabetes mellitus: Without complications	
Diabetes	ICD-10	E11	Non-insulin-dependent diabetes mellitus	
Diabetes	ICD-10	E110	Non-insulin-dependent diabetes mellitus: With coma	
Diabetes	ICD-10	E111	Non-insulin-dependent diabetes mellitus: With ketoacidosis	
Diabetes	ICD-10	E112	Non-insulin-dependent diabetes mellitus: With renal complications	

Continued on next page

Defined	System	Code	Description	Notes
Diabetes	ICD-10	E113	Non-insulin-dependent diabetes mellitus: With ophthalmic complications	
Diabetes	ICD-10	E114	Non-insulin-dependent diabetes mellitus: With neurological complications	
Diabetes	ICD-10	E115	Non-insulin-dependent diabetes mellitus: With peripheral circulatory complications	
Diabetes	ICD-10	E116	Non-insulin-dependent diabetes mellitus: With other specified complications	
Diabetes	ICD-10	E117	Non-insulin-dependent diabetes mellitus: With multiple complications	
Diabetes	ICD-10	E118	Non-insulin-dependent diabetes mellitus: With unspecified complications	
Diabetes	ICD-10	E119	Non-insulin-dependent diabetes mellitus: Without complications	
Diabetes	ICD-10	E14	Unspecified diabetes mellitus	
Diabetes	ICD-10	E140	Unspecified diabetes mellitus: With coma	
Diabetes	ICD-10	E141	Unspecified diabetes mellitus: With ketoacidosis	
Diabetes	ICD-10	E142	Unspecified diabetes mellitus: With renal complications	
Diabetes	ICD-10	E143	Unspecified diabetes mellitus: With ophthalmic complications	
Diabetes	ICD-10	E144	Unspecified diabetes mellitus: With neurological complications	
Diabetes	ICD-10	E145	Unspecified diabetes mellitus: With peripheral circulatory complications	
Diabetes	ICD-10	E146	Unspecified diabetes mellitus: With other specified complications	

Continued on next page

Table E.1 – *Continued from previous page*

Defined	System	Code	Description	Notes
Diabetes	ICD-10	E147	Unspecified diabetes mellitus: With multiple complications	
Diabetes	ICD-10	E148	Unspecified diabetes mellitus: With unspecified complications	
Diabetes	ICD-10	E149	Unspecified diabetes mellitus: Without complications	
Diabetes	Read v2	66A..	Diabetic monitoring	
Diabetes	Read v2	66AS.	Diabetic annual review	
Diabetes	Read v2	68A7.	Diabetic retinopathy screening	
Diabetes	Read v2	9OL..	Diabetes monitoring admin.	
Diabetes	Read v2	9OL4.	Diabetes monitoring 1st letter	
Diabetes	Read v2	C10F.	Type 2 diabetes mellitus	
Diabetes	Read v2	66AP.	Diabetes: practice programme	
Diabetes	Read v2	66A4.	Diabetic on oral treatment	
Diabetes	Read v2	66A2.	Follow-up diabetic assessment	
Diabetes	Read v2	C10..	[X]Diabetes mellitus	
Diabetes	Read v2	Cyu2.	[X]Diabetes mellitus	
Diabetes	Read v2	66A3.	Diabetic on diet only	
Diabetes	Read v2	66Ac.	Diabetic periph neurop screen	
Diabetes	Read v2	9NND.	Under care of diab foot screen	
Diabetes	Read v2	2G5E.	O/E - R diab foot at low risk	
Diabetes	Read v2	2G5I.	O/E - L diab foot at low risk	
Diabetes	Read v2	66A5.	Diabetic on insulin	

Continued on next page

Defined	System	Code	Description	Notes
Diabetes	Read v2	8B3l.	Diabetes medication review	
Diabetes	Read v2	13AB.	Diabetic lipid lowering diet	
Diabetes	Read v2	C109.	Non-insulin depd diabetes mell	
Diabetes	Read v2	9OLA.	Diabetes monitor. check done	
Diabetes	Read v2	9OL5.	Diabetes monitoring 2nd letter	
Diabetes	Read v2	66AD.	Fundoscopy - diabetic check	
Diabetes	Read v2	F4200	Background diabetic retinopath	
Diabetes	Read v2	66Aq.	Diabetic foot screen	
Diabetes	Read v2	66AZ.	Diabetic monitoring NOS	
Diabetes	Read v2	66A8.	Has seen dietician - diabetes	
Diabetes	Read v2	8BL2.	Pt on max tol ther for diabet	
Diabetes	Read v2	F420.	Diabetic retinopathy	
Diabetes	Read v2	C10E.	Type 1 diabetes mellitus	
Diabetes	Read v2	66Ab.	Diabetic foot examination	
Diabetes	Read v2	66AI.	Diabetic - good control	
Diabetes	Read v2	66AY.	Diabetic diet-good compliance	
Diabetes	Read v2	679L.	Health education - diabetes	
Diabetes	Read v2	66AU.	Diabetes care by hospital only	
Diabetes	Read v2	9OL6.	Diabetes monitoring 3rd letter	
Diabetes	Read v2	66AR.	Diabetes management plan given	
Diabetes	Read v2	13AC.	Diabetic weight reducing diet	
Diabetes	Read v2	66AJ.	Diabetic - poor control	

Continued on next page

Table E.1 – *Continued from previous page*

Defined	System	Code	Description	Notes
Diabetes	Read v2	C1001	Diab.mell.no comp. - adult	
Diabetes	Read v2	2BBF.	Retina abnormal - diabet relat	
Diabetes	Read v2	C109J	Insul treated Type 2 diab mell	
Diabetes	Read v2	C10FJ	Insul treated Type 2 diab mell	
Diabetes	Read v2	8Hl1.	Ref diabetc retinopathy screen	
Diabetes	Read v2	2G5F.	O/E - R diab foot at mod risk	
Diabetes	Read v2	2G5J.	O/E - L diab foot at mod risk	
Diabetes	Read v2	66A9.	Understands diet - diabetes	
Diabetes	Read v2	9OL7.	Diabetes monitor.verbal invite	
Diabetes	Read v2	66AW.	Diabetic foot risk assessment	
Diabetes	Read v2	9OL8.	Diabetes monitor.phone invite	
Diabetes	Read v2	C108.	Insulin depnd diabetes melitus	
Diabetes	Read v2	F4204	Diabetic maculopathy	
Diabetes	Read v2	66AT.	Annual diabetic blood test	
Diabetes	Read v2	1434.	H/O: diabetes mellitus	
Diabetes	Read v2	66Aa.	Diabetic diet-poor compliance	
Diabetes	Read v2	8I3W.	Diabetic foot exam declined	
Diabetes	Read v2	8HBG.	Diab retinopathy 12 mth review	
Diabetes	Read v2	2G5G.	O/E - R diab foot at high risk	
Diabetes	Read v2	F372.	Polyneuropathy in diabetes	
Diabetes	Read v2	2G5K.	O/E - L diab foot at high risk	
Diabetes	Read v2	9OLZ.	Diabetes monitoring admin.NOS	

Continued on next page

Table E.1 – *Continued from previous page*

Defined	System	Code	Description	Notes
Diabetes	Read v2	68A9.	Diabetic retinopathy scr offer	
Diabetes	Read v2	C1000	Diab.mell.no comp. - juvenile	
Diabetes	Read v2	F4201	Proliferative diabetic retinop	
Diabetes	Read v2	66AV.	Diabetic on insulin+oral treat	
Diabetes	Read v2	9h4..	Except report: diabet qual ind	
Diabetes	Read v2	8I3X.	Diab retinopath screen refused	
Diabetes	Read v2	9OLD.	Diabet pt unsuit dig ret photo	
Diabetes	Read v2	9OL3.	Diabetes monitoring default	
Diabetes	Read v2	C101.	Diab.mell.with ketoacidosis	
Diabetes	Read v2	9360.	Pt held diabetic record issued	
Diabetes	Read v2	F4202	Preproliferative diabetic ret	
Diabetes	Read v2	C104.	Diab.mell. with nephropathy	
Diabetes	Read v2	66AH.	Diabetic treatment changed	
Diabetes	Read v2	2G5B.	O/E-Left diabet foot at risk	
Diabetes	Read v2	66AK.	Diabetic - cooperative patient	
Diabetes	Read v2	2G5A.	O/E-Right diabet foot at risk	
Diabetes	Read v2	2BBL.	O/E - diabet maculop both eyes	
Diabetes	Read v2	7276.	Pan retinal photocoag diabetes	
Diabetes	Read v2	C106.	Diab.mell. with neuropathy	
Diabetes	Read v2	F4206	Non prolif diab retinop	
Diabetes	Read v2	F4640	Diabetic cataract	
Diabetes	Read v2	9Non.	Seen community diab spec clin	

Continued on next page

Table E.1 – *Continued from previous page*

Defined	System	Code	Description	Notes
Diabetes	Read v2	66A0.	Diabetes type 2 review	
Diabetes	Read v2	9NM0.	Attending diabetes clinic	
Diabetes	Read v2	8Hj4.	Refer to DESMOND diab st ed pr	
Diabetes	Read v2	66AJz	Diabetic - poor control NOS	
Diabetes	Read v2	C100.	Diab.mell. - no complication	
Diabetes	Read v2	679L0	Educa self management diabetes	
Diabetes	Read v2	8HHy.	Referral to diabetic register	
Diabetes	Read v2	66Af.	Pt diabetes education review	
Diabetes	Read v2	C1097	Type 2 diab mell+poor control	
Diabetes	Read v2	C10F7	Type 2 diab mell+poor control	
Diabetes	Read v2	F420z	Diabetic retinopathy NOS	
Diabetes	Read v2	9OL2.	Refuses diabetes monitoring	
Diabetes	Read v2	8H2J.	Admit diabetic emergency	
Diabetes	Read v2	F3722	Asymptomatic diab neuropathy	
Diabetes	Read v2	679R.	Pt offered diab struct ed prog	
Diabetes	Read v2	66AN.	Date diabetic treatment start	
Diabetes	Read v2	M2711	Neuropathic diab ulcer - foot	
Diabetes	Read v2	9m0A.	Declined diabetic retinop scrn	
Diabetes	Read v2	F1711	Autonomic neuropathy-diabetes	
Diabetes	Read v2	M2712	Mixed diabetic ulcer - foot	
Diabetes	Read v2	8A13.	Diabetic stabilisation	
Diabetes	Read v2	F3721	Chron painful diab neuropathy	

Continued on next page

Table E.1 – *Continued from previous page*

Defined	System	Code	Description	Notes
Diabetes	Read v2	M2710	Ischaemic ulcer diabetic foot	
Diabetes	Read v2	2G5H.	O/E - R diab foot - ulcerated	
Diabetes	Read v2	F4203	Advanced diabetic maculopathy	
Diabetes	Read v2	2G5L.	O/E - L diab foot - ulcerated	
Diabetes	Read v2	2G510	Foot abnormal-diabetes related	
Diabetes	Read v2	2G5C.	Foot abnormal-diabetes related	
Diabetes	Read v2	C1096	Type 2 diab mell + retinopathy	
Diabetes	Read v2	C10F6	Type 2 diab mell + retinopathy	
Diabetes	Read v2	C10FC	Type 2 diab mell + nephropathy	
Diabetes	Read v2	9OLM.	Diabetes struc edu prog declin	
Diabetes	Read v2	66AL.	Diabetic-uncooperative patient	
Diabetes	Read v2	M0372	Cellulitis in diabetic foot	
Diabetes	Read v2	C105.	Diab.mell.+ eye manifestation	
Diabetes	Read v2	C1099	Non-insul-dep diab mel no comp	
Diabetes	Read v2	N0301	Diabetic Charcot arthropathy	
Diabetes	Read v2	8HBH.	Diab retinopathy 6 mth review	
Diabetes	Read v2	C107.	Diab.mell.+periph.circul.dis	
Diabetes	Read v2	C1087	Type 1 diab mell + retinopathy	
Diabetes	Read v2	C10E7	Type 1 diab mell + retinopathy	
Diabetes	Read v2	8CR2.	Diabetes clin management plan	
Diabetes	Read v2	C1088	Type 1 diab mell poor control	
Diabetes	Read v2	C10E8	Type 1 diab mell poor control	

Continued on next page

Table E.1 – *Continued from previous page*

Defined	System	Code	Description	Notes
Diabetes	Read v2	C1090	Type 2 diab mell + renal compl	
Diabetes	Read v2	C10F0	Type 2 diab mell + renal compl	
Diabetes	Read v2	C10F9	Type 2 diab mell without comp	
Diabetes	Read v2	N0300	Diabetic cheiroarthropathy	
Diabetes	Read v2	C1089	Type 1 diab mell matur onset	
Diabetes	Read v2	C10E9	Type 1 diab mell matur onset	
Diabetes	Read v2	66AJ1	Brittle diabetes	
Diabetes	Read v2	68AB.	Diabtic dig retnpthy scrn offd	
Diabetes	Read v2	F3720	Acute painful diab neuropathy	
Diabetes	Read v2	66At1	Type II diabetic dietary revie	
Diabetes	Read v2	F3y0.	Diabetic mononeuropathy	
Diabetes	Read v2	8CS0.	Diabetes care plan agreed	
Diabetes	Read v2	C101z	Diab.mell.+ketoacid -onset NOS	
Diabetes	Read v2	C106z	Diab.mell.+neuropathy NOS	
Diabetes	Read v2	C10ED	Type 1 diab mell + nephropathy	
Diabetes	Read v2	F3813	Myasthenic syndrome+diabetes	
Diabetes	Read v2	8A12.	Diabetic crisis monitoring	
Diabetes	Read v2	C104z	Diab.mell.+nephropathy NOS	
Diabetes	Read v2	C1094	Type 2 diab mell with ulcer	
Diabetes	Read v2	C10F4	Type 2 diab mell with ulcer	
Diabetes	Read v2	C1061	Diab.mell.+neuropathy - adult	
Diabetes	Read v2	K01x1	Nephrotic syndrome+diabetes M.	

Continued on next page

Table E.1 – *Continued from previous page*

Defined	System	Code	Description	Notes
Diabetes	Read v2	C1011	Diab.mell.+ketoacid - adult	
Diabetes	Read v2	F35z0	Diabetic mononeuritis NOS	
Diabetes	Read v2	F4205	Advanced diabetic retinal dis	
Diabetes	Read v2	R0542	[D]Gangrene of toe in diabetic	
Diabetes	Read v2	C1092	Type 2 diab mell + neurol comp	
Diabetes	Read v2	C10F2	Type 2 diab mell + neurol comp	
Diabetes	Read v2	C10ER	Latent autoimm diab mell adult	
Diabetes	Read v2	C103.	Diab.mell. + ketoacidotic coma	
Diabetes	Read v2	C10EE	Type 1 diab mell + hypo coma	
Diabetes	Read v2	C1085	Type 1 diab mell with ulcer	
Diabetes	Read v2	C10E5	Type 1 diab mell with ulcer	
Diabetes	Read v2	C109E	NIDDM with diabetic cataract	
Diabetes	Read v2	9OLF.	Diabetes struc ed prog complet	
Diabetes	Read v2	C100z	Diab.mell.no comp. - onset NOS	
Diabetes	Read v2	F4407	Diabetic iritis	
Diabetes	Read v2	8H3O.	Non-urgent diabetic admission	
Diabetes	Read v2	C10C.	Diab mell aut dom	
Diabetes	Read v2	C10z.	Diab.mell. + unspec comp	
Diabetes	Read v2	C1095	Type 2 diab mell + gangrene	
Diabetes	Read v2	C10F5	Type 2 diab mell + gangrene	
Diabetes	Read v2	C10FH	Type 2 diab mell neurop+arthr	
Diabetes	Read v2	C10E4	Unstab type 1 diabet mellitus	

Continued on next page

Table E.1 – *Continued from previous page*

Defined	System	Code	Description	Notes
Diabetes	Read v2	G73y0	Diabetic peripheral angiopathy	
Diabetes	Read v2	C1081	Type 1 diab mell + ophth comps	
Diabetes	Read v2	C10E1	Type 1 diab mell + ophth comps	
Diabetes	Read v2	C1091	Type 2 diab mell+ophthal comp	
Diabetes	Read v2	C10F1	Type 2 diab mell+ophthal comp	
Diabetes	Read v2	L1806	Pre-ex diab mel non insuln-dep	
Diabetes	Read v2	C10FG	Type 2 diab mell + arthropathy	
Diabetes	Read v2	2G5V.	O/E - R chron diab foot ulcer	
Diabetes	Read v2	C10FE	Type 2 diab mell+diab catarct	
Diabetes	Read v2	2G5W.	O/E - L chron diab foot ulcer	
Diabetes	Read v2	C10FB	Type 2 diab mell + polyneurop	
Diabetes	Read v2	C10FD	Type 2 diab mell+hypogly coma	
Diabetes	Read v2	8HLE.	Diabetology D.V. done	
Diabetes	Read v2	C1084	Unstab insul depend diab mell	
Diabetes	Read v2	C1041	Diab.mell.+nephropathy - adult	
Diabetes	Read v2	C10D.	Diab mell aut dom type 2	
Diabetes	Read v2	C1051	Diab.mell.+eye manif - adult	
Diabetes	Read v2	C10N1	Cyst fibro relat diab mellitus	
Diabetes	Read v2	C1086	Type 1 diab mell with gangrene	
Diabetes	Read v2	C10E6	Type 1 diab mell with gangrene	
Diabetes	Read v2	C10FA	Type 2 diab mell mononeurop	
Diabetes	Read v2	C10EJ	Type 1 diab mell+neuro arthrop	

Continued on next page

Table E.1 – *Continued from previous page*

Defined	System	Code	Description	Notes
Diabetes	Read v2	C10N.	Secondary diabetes mellitus	
Diabetes	Read v2	C102.	Diab.mell. + hyperosmolar coma	
Diabetes	Read v2	C10EC	Type 1 diab mell + polyneurop	
Diabetes	Read v2	C10y.	Diab.mell.+other manifestation	
Diabetes	Read v2	C1080	Insuln-dep diab mel+renal comp	
Diabetes	Read v2	C101y	Oth specfd diab mel+ketoacidos	
Diabetes	Read v2	C1083	Type 1 diab mell + mult comps	
Diabetes	Read v2	C10E3	Type 1 diab mell + mult comps	
Diabetes	Read v2	F3450	Diabet mononeuritis multiplex	
Diabetes	Read v2	C103z	Diab.mell.+ketoac coma NOS	
Diabetes	Read v2	C10z1	Diab.mell.+comp NOS - adult	
Diabetes	Read v2	C10EA	Type 1 diab mell without comp	
Diabetes	Read v2	C10F3	Type 2 diab mell + multip comp	
Diabetes	Read v2	C1072	Diabetic gangrene - adult	
Diabetes	Read v2	C10y1	Diab.mell.+other manif. -adult	
Diabetes	Read v2	C108F	IDDM with diabetic cataract	
Diabetes	Read v2	C103y	Oth specif diab mell with coma	
Diabetes	Read v2	C1021	Diab.mell.+hyperosm.coma-adult	
Diabetes	Read v2	C10A.	Malnutritn-relat diab mellitus	
Diabetes	Read v2	C1082	Type 1 diab mell + neuro comps	
Diabetes	Read v2	C10E2	Type 1 diab mell + neuro comps	
Diabetes	Read v2	C1050	Diab.mell.+eye manif -juvenile	

Continued on next page

Table E.1 – *Continued from previous page*

Defined	System	Code	Description	Notes
Diabetes	Read v2	Cyu20	[X]Oth specf diabetes mellitus	
Diabetes	Read v2	C102z	Diabetes+hyperosmolar coma NOS	
Diabetes	Read v2	C109F	NIDDM with periph angiopath	
Diabetes	Read v2	TJ23z	AR - insulins/antidiabetic NOS	
Diabetes	Read v2	6761.	Diabetic pre-pregnancy counsel	
Diabetes	Read v2	C10yy	Oth spec diab mel+oth spec cmp	
Diabetes	Read v2	C108G	IDDM with peripheral angiopath	
Diabetes	Read v2	C10EH	Type 1 diab mell + arthropathy	
Diabetes	Read v2	R0543	[D]Widespread diab foot gangr	
Diabetes	Read v2	C10zz	Diab.mell. + unspec comp NOS	
Diabetes	Read v2	C10EB	Type 1 diab mell + mononeurop	
Diabetes	Read v2	3883.	Diabetes treatmt satisf quest	
Diabetes	Read v2	9M00.	Informd consent diab nat audit	
Diabetes	Read v2	L1800	Preg.+diabetes mellitus unspec	
Diabetes	Read v2	C10z0	Diab.mell.+comp NOS - juvenile	
Diabetes	Read v2	2BBr.	Impair vision due diab retinop	
Diabetes	Read v2	C105y	Oth specfd diab mel+ophth comp	
Smoking ascertainment	Read v2	1371.	Never smoked tobacco	
Smoking ascertainment	Read v2	137S.	Ex smoker	
Smoking ascertainment	Read v2	137P.	Cigarette smoker	

Continued on next page

Table E.1 – *Continued from previous page*

Defined	System	Code	Description	Notes
Smoking ascertainment	Read v2	137L.	Current non-smoker	
Smoking ascertainment	Read v2	137R.	Current smoker	
Smoking ascertainment	Read v2	1374.	Moderate smoker - 10-19 cigs/d	
Smoking ascertainment	Read v2	1373.	Light smoker - 1-9 cigs/day	
Smoking ascertainment	Read v2	1379.	Ex-moderate smoker (10-19/day)	
Smoking ascertainment	Read v2	1375.	Heavy smoker - 20-39 cigs/day	
Smoking ascertainment	Read v2	137K.	Stopped smoking	
Smoking ascertainment	Read v2	1378.	Ex-light smoker (1-9/day)	
Smoking ascertainment	Read v2	137G.	Trying to give up smoking	
Smoking ascertainment	Read v2	137A.	Ex-heavy smoker (20-39/day)	
Smoking ascertainment	Read v2	137F.	Ex-smoker - amount unknown	
Smoking ascertainment	Read v2	1372.	Trivial smoker - < 1 cig/day	
Smoking ascertainment	Read v2	137M.	Rolls own cigarettes	

Continued on next page

Table E.1 – *Continued from previous page*

Defined	System	Code	Description	Notes
Smoking ascertainment	Read v2	137Z.	Tobacco consumption NOS	
Smoking ascertainment	Read v2	1377.	Ex-trivial smoker (<1/day)	
Smoking ascertainment	Read v2	137T.	Date ceased smoking	
Smoking ascertainment	Read v2	137H.	Pipe smoker	
Smoking ascertainment	Read v2	137J.	Cigar smoker	
Smoking ascertainment	Read v2	137B.	Ex-very heavy smoker (40+/day)	
Smoking ascertainment	Read v2	1376.	Very heavy smoker - 40+cigs/d	
Smoking ascertainment	Read v2	137X.	Cigarette consumption	
Smoking ascertainment	Read v2	137N.	Ex pipe smoker	
Smoking ascertainment	Read v2	137Q.	Smoking started	
Smoking ascertainment	Read v2	137O.	Ex cigar smoker	
Smoking ascertainment	Read v2	137Y.	Cigar consumption	
Smoking ascertainment	Read v2	137V.	Smoking reduced	

Continued on next page

Table E.1 – *Continued from previous page*

Defined	System	Code	Description	Notes
Smoking ascertainment	Read v2	137K0	Recently stopped smoking	
Smoking ascertainment	Read v2	9kn..	Non-smoker annual review - enhanced services administration	
Smoking ascertainment	Read v2	9ko..	Current smoker annual review - enhanced services administration	
Smoking advice	Read v2	8CAL.	Smoking cessation advice	
Smoking advice	Read v2	6791.	Health ed. - smoking	
Smoking advice	Read v2	67H1.	Lifestyle adv re smoking	
Smoking advice	Read v2	67H6.	Brf intervention smoking cessn	
Smoking cessation referral	Read v2	8H7i.	Referral: smok cessatn advisor	
Smoking cessation referral	Read v2	8HTK.	Referl to stop-smoking clinic	
Smoking cessation referral	Read v2	9N2k.	Seen by smoking cesstn advisor	
Smoking cessation referral	Read v2	9N4M.	DNA - Smoking cessation clinic	
Smoking cessation referral	Read v2	13p5.	Smoking cessn progm start date	

Continued on next page

Table E.1 – *Continued from previous page*

Defined	System	Code	Description	Notes
Smoking cessation referral	Read v2	8HkQ.	Refer to NHS stop smoking srvc	
Chronic renal disease	Read v2	66i..	CKD monitoring	
Chronic renal disease	Read v2	9Ot0.	CKD monitoring first letter	
Chronic renal disease	Read v2	K05..	Chronic renal failure	
Chronic renal disease	Read v2	1Z1..	Chronic renal impairment	
Chronic renal disease	Read v2	K060.	Renal impairment	
Chronic renal disease	Read v2	1Z1B.	CKD stage 3 with proteinuria	
Chronic renal disease	Read v2	9Ot1.	CKD monitoring second letter	
Chronic renal disease	Read v2	9Ot..	CKD monitoring administration	
Chronic renal disease	Read v2	9Ot4.	CKD monitoring telephone invte	
Chronic renal disease	Read v2	9Ot2.	CKD monitoring third letter	
Chronic renal disease	Read v2	K08..	Impaired renal function disord	
Chronic renal disease	Read v2	K050.	End stage renal failure	

Continued on next page

Table E.1 – *Continued from previous page*

Defined	System	Code	Description	Notes
Chronic renal disease	Read v2	9Ot3.	CKD monitoring verbal invite	
Chronic renal disease	Read v2	1Z1H.	CKD stage 4 with proteinuria	
Chronic renal disease	Read v2	1Z1D.	CKD stage 3A with proteinuria	
Chronic renal disease	Read v2	1Z1F.	CKD stage 3B with proteinuria	
Chronic renal disease	Read v2	1Z19.	CKD stage 2 with proteinuria	
Chronic renal disease	Read v2	Kyu2.	[X]Renal failure	
Chronic renal disease	Read v2	K08z.	Impaired renal funct.dis.NOS	
Chronic renal disease	Read v2	D2150	Anaemia secondary to CRF	
Chronic renal disease	Read v2	D215.	Anaemia second renal failure	
Chronic renal disease	Read v2	1Z1K.	CKD stage 5 with proteinuria	
Chronic renal disease	Read v2	K0E..	Acute-on-chronic renal failure	
Chronic renal disease	Read v2	Kyu21	[X]Other chronic renal failure	
Chronic renal disease	Read v2	G222.	Hypertens renal dis+renal fail	

Continued on next page

Table E.1 – *Continued from previous page*

Defined	System	Code	Description	Notes
Chronic renal disease	Read v2	G233.	Hypertn hrt+ren dis+renal fail	
Lipid disorders	Read v2	C3200	LDL hyperlipoproteinaemia	
Peripheral vascular disease	ICD-10	I702	Atherosclerosis of arteries of extremities	
Peripheral vascular disease	ICD-10	I708	Atherosclerosis of other arteries	
Peripheral vascular disease	ICD-10	I709	Generalized and unspecified atherosclerosis	
Peripheral vascular disease	ICD-10	I739	Peripheral vascular disease, unspecified	
Peripheral vascular disease	ICD-10	I792	Peripheral angiopathy in diseases classified elsewhere	
Old MI	ICD-10	I252	Old myocardial infarction	
Old MI	Read v2	G30..	Acute myocardial infarction	
Old MI	Read v2	G3115	Acute coronary syndrome	
Old MI	Read v2	G3111	Unstable angina	
Old MI	Read v2	G32..	Old myocardial infarction	
Old MI	Read v2	G308.	Inferior myocard. infarct NOS	
Old MI	Read v2	14A3.	H/O: myocardial infarct <60	
Old MI	Read v2	G30z.	Acute myocardial infarct. NOS	
Old MI	Read v2	G301z	Anterior myocard.infarct NOS	
Old MI	Read v2	G301.	Anterior myocard. infarct OS	
Old MI	Read v2	323..	ECG: myocardial infarction	

Continued on next page

Table E.1 – *Continued from previous page*

Defined	System	Code	Description	Notes
Old MI	Read v2	G31y0	Acute coronary insufficiency	
Old MI	Read v2	G3112	Angina at rest	
Old MI	Read v2	14A4.	H/O: myocardial infarct >60	
Old MI	Read v2	3232.	ECG: old myocardial infarction	
Old MI	Read v2	G304.	Posterior myocard.infarct NOS	
Old MI	Read v2	G305.	Lateral myocardial infarct NOS	
Old MI	Read v2	G35..	Subseqnt myocardial infarction	
Old MI	Read v2	G30y.	Other acute myocardial infarct	
Old MI	Read v2	323Z.	ECG: myocardial infarct NOS	
Old MI	Read v2	14AT.	H/O: myocardial infarction	
Old MI	Read v2	G30yz	Other acute myocardial inf.NOS	
Old MI	Read v2	14AH.	H/O: Myoc infarct in last year	
Old MI	Read v2	G351.	Subsqnt myocrd infarc/inf wall	
Old MI	Read v2	ZV719	[V]Obs/suspct myocard infarctn	
Old MI	Read v2	G306.	True posterior myocard.infarct	
Old MI	Read v2	G350.	Subsqnt myocrd infarc/ant wall	
Old MI	Read v2	G30B.	Acute posterol myocard infarct	
Old MI	Read v2	G33z0	Status anginosus	
MI	ICD-10	I21	Acute myocardial infarction	
MI	ICD-10	I210	Acute transmural myocardial infarction of anterior wall	
MI	ICD-10	I211	Acute transmural myocardial infarction of inferior wall	
MI	ICD-10	I212	Acute transmural myocardial infarction of other sites	

Continued on next page

Table E.1 – *Continued from previous page*

Defined	System	Code	Description	Notes
MI	ICD-10	I213	Acute transmural myocardial infarction of unspecified site	
MI	ICD-10	I214	Acute subendocardial myocardial infarction	
MI	ICD-10	I219	Acute myocardial infarction, unspecified	
MI	ICD-10	I22	Subsequent myocardial infarction	
MI	ICD-10	I220	Subsequent myocardial infarction of anterior wall	
MI	ICD-10	I221	Subsequent myocardial infarction of inferior wall	
MI	ICD-10	I228	Subsequent myocardial infarction of other sites	
MI	ICD-10	I229	Subsequent myocardial infarction of unspecified site	
Unstable angina	ICD-10	I200	Unstable angina	
Hypertension diagnosis	ICD-10	I110	Hypertensive heart disease with (congestive) heart failure	
Hypertension diagnosis	ICD-10	I119	Hypertensive heart disease without (congestive) heart failure	
Hypertension diagnosis	ICD-10	I120	Hypertensive renal disease with renal failure	
Hypertension diagnosis	ICD-10	I129	Hypertensive renal disease without renal failure	
Hypertension diagnosis	ICD-10	I130	Hypertensive heart and renal disease with (congestive) heart failure	
Hypertension diagnosis	ICD-10	I131	Hypertensive heart and renal disease with renal failure	
Hypertension diagnosis	ICD-10	I132	Hypertensive heart and renal disease with both (congestive) heart failure and renal failure	
Hypertension diagnosis	ICD-10	I139	Hypertensive heart and renal disease, unspecified	

Continued on next page

Table E.1 – *Continued from previous page*

Defined	System	Code	Description	Notes
Hypertension diagnosis	ICD-10	I150	Renovascular hypertension	
Hypertension diagnosis	ICD-10	I151	Hypertension secondary to other renal disorders	
Hypertension diagnosis	ICD-10	I152	Hypertension secondary to endocrine disorders	
Hypertension diagnosis	ICD-10	I158	Other secondary hypertension	
Hypertension diagnosis	ICD-10	I159	Secondary hypertension, unspecified	
Hypertension diagnosis	Read v2	G20..	Essential hypertension	
Hypertension diagnosis	Read v2	G2...	Hypertensive disease	
Hypertension diagnosis	Read v2	14A2.	H/O: hypertension	
Hypertension diagnosis	Read v2	G20z.	Essential hypertension NOS	
Hypertension diagnosis	Read v2	G201.	Benign essential hypertension	
Hypertension diagnosis	Read v2	G2z..	Hypertensive disease NOS	
Hypertension diagnosis	Read v2	G202.	Systolic hypertension	
Hypertension diagnosis	Read v2	G2y..	Hypertensive disease OS	

Continued on next page

Table E.1 – *Continued from previous page*

Defined	System	Code	Description	Notes
Hypertension diagnosis	Read v2	G200.	Malignant essential hypertension	
Hypertension diagnosis	Read v2	G203.	Diastolic hypertension	
Hypertension diagnosis	Read v2	Gyu2.	[X]Hypertensive diseases	
CVA	ICD-10	I110	Hypertensive heart disease with (congestive) heart failure	
CVA	ICD-10	I119	Hypertensive heart disease without (congestive) heart failure	
CVA	ICD-10	I120	Hypertensive renal disease with renal failure	
CVA	ICD-10	I129	Hypertensive renal disease without renal failure	
CVA	ICD-10	I130	Hypertensive heart and renal disease with (congestive) heart failure	
CVA	ICD-10	I131	Hypertensive heart and renal disease with renal failure	
CVA	ICD-10	I132	Hypertensive heart and renal disease with both (congestive) heart failure and renal failure	
CVA	ICD-10	I139	Hypertensive heart and renal disease, unspecified	
CVA	ICD-10	I150	Renovascular hypertension	
CVA	ICD-10	I151	Hypertension secondary to other renal disorders	
CVA	ICD-10	I152	Hypertension secondary to endocrine disorders	
CVA	ICD-10	I158	Other secondary hypertension	
CVA	ICD-10	I159	Secondary hypertension, unspecified	
CVA	Read v2	G65..	Transient cerebral ischaemia	
CVA	Read v2	G66..	Stroke/CVA unspecified	

Continued on next page

Table E.1 – *Continued from previous page*

Defined	System	Code	Description	Notes
CVA	Read v2	662M.	Stroke monitoring	
CVA	Read v2	9Om0.	Stroke/TIA monitor 1st letter	
CVA	Read v2	662e.	Stroke/CVA annual review	
CVA	Read v2	G6...	[X]Cerebrovascular diseases	
CVA	Read v2	Gyu6.	[X]Cerebrovascular diseases	
CVA	Read v2	9h21.	Except stroke qual ind: Pt uns	
CVA	Read v2	G64..	Cerebral arterial occlusion	
CVA	Read v2	14A7.	H/O: CVA/stroke	
CVA	Read v2	9h22.	Exc stroke qual ind: Infor dis	
CVA	Read v2	8HBJ.	Stroke / TIA referral	
CVA	Read v2	9Om1.	Stroke/TIA monitor 2nd letter	
CVA	Read v2	8HTQ.	Referral to stroke clinic	
CVA	Read v2	G64z.	Cerebral infarction NOS	
CVA	Read v2	9N0p.	Seen in stroke clinic	
CVA	Read v2	14AB.	H/O: TIA	
CVA	Read v2	9N4X.	Did not attend stroke clinic	
CVA	Read v2	9Om2.	Stroke/TIA monitor 3rd letter	
CVA	Read v2	G667.	Left sided CVA	
CVA	Read v2	388I.	Stroke risk	
CVA	Read v2	9h2..	Except report: stroke qual ind	
CVA	Read v2	G668.	Right sided CVA	
CVA	Read v2	G65z.	Transient cerebral ischaem.NOS	

Continued on next page

Table E.1 – *Continued from previous page*

Defined	System	Code	Description	Notes
CVA	Read v2	G65zz	Transient cerebral ischaem.NOS	
CVA	Read v2	9Om..	Stroke/TIA monitoring admin	
CVA	Read v2	1JA1.	Suspect cerebrovasculr disease	
CVA	Read v2	9Om4.	Stroke/TIA monitr phone invite	
CVA	Read v2	9Om3.	Stroke/TIA monitor verb invit	
CVA	Read v2	G6z..	Cerebrovascular disease NOS	
CVA	Read v2	G64z2	Left sided cerebral infarction	
CVA	Read v2	G640.	Cerebral thrombosis	
CVA	Read v2	6F...	Stroke prevention	
CVA	Read v2	G64z3	Right sided cerebral infarct	
CVA	Read v2	G63y0	Cerebr infct/throm/precere art	
CVA	Read v2	G6711	Chronic cerebral ischaemia	
CVA	Read v2	G663.	Brain stem stroke syndrome	
CVA	Read v2	G641.	Cerebral embolism	
CVA	Read v2	G664.	Cerebellar stroke syndrome	
CVA	Read v2	G67..	Other cerebrovascular disease	
CVA	Read v2	7A252	Embolisation cerebral art NEC	
CVA	Read v2	Gyu64	[X]Other cerebral infarction	
CVA	Read v2	G670.	Cerebral atherosclerosis	
CVA	Read v2	1JK..	Suspected TIA	
CVA	Read v2	G660.	Middle cerebral artery syndrm	
CVA	Read v2	7A250	PC TL embolisation cerebr art	

Continued on next page

Table E.1 – *Continued from previous page*

Defined	System	Code	Description	Notes
CVA	Read v2	1477.	H/O: cerebrovascular disease	
CVA	Read v2	G6400	Cerebr infct/throm/cerebrl art	
CVA	Read v2	G65y.	Other transient cerebral isch.	
CVA	Read v2	1JA10	Suspct cerebrovasclar accident	
CVA	Read v2	G6y..	Cerebrovascular disease OS	
CVA	Read v2	Fyu55	[X]Oth cerebral TIA's+rel synd	
CVA	Read v2	G63y1	Cerebr infct/embol/precere art	
CVA	Read v2	G63..	Precerebral arterial occlusion	
CVA	Read v2	G68X.	Seql/strok,n spc/h'm,infarc	
CVA	Read v2	G65z1	Intermittent CVA	
CVA	Read v2	38DM.	ABCD2 stroke risk score	
CVA	Read v2	G68..	Cerebrovasc.dis.-late effects	
CVA	Read v2	G6410	Cerebr infct/embol/cerebrl art	
CVA	Read v2	G661.	Anterior cerebral artery syn	
CVA	Read v2	G67z.	Other cerebrovasc.disease NOS	
CVA	Read v2	14AK.	H/O: Stroke in last year	
CVA	Read v2	7A246	Open embolisation cerebral art	
CVA	Read v2	G63z.	Precerebral artery occlus. NOS	
CVA	Read v2	G683.	Sequelae/cerebral infarction	
CVA	Read v2	G63y.	Other precerebral artery occl.	
CVA	Read v2	8HHM.	Ref to stroke func improv serv	
CVA	Read v2	1M4..	Central post-stroke pain	

Continued on next page

Table E.1 – *Continued from previous page*

Defined	System	Code	Description	Notes
CVA	Read v2	Gyu66	[X]Oc+sten/o cerebral arteries	
CVA	Read v2	7A244	Open embolectomy cerebral art	
CVA	Read v2	G65z0	Impending CVA	
CVA	Read v2	Gyu65	[X]Oc+steno/o precerebral artr	
CVA	Read v2	G654.	Multi+bilat precerebrl art syn	
CVA	Read v2	Gyu67	[X]Other spcfd cerebrovasc dis	
CVA	Read v2	Gyu6A	[X]Oth cerebrovasc diso/dis CE	
Beta-blocker	Read v2	G65..	Transient cerebral ischaemia	Antihypertensive
Beta-blocker	Read v2	G66..	Stroke/CVA unspecified	Antihypertensive
Beta-blocker	Read v2	662M.	Stroke monitoring	Antihypertensive
Beta-blocker	Read v2	9Om0.	Stroke/TIA monitor 1st letter	Antihypertensive
Beta-blocker	Read v2	662e.	Stroke/CVA annual review	Antihypertensive
Beta-blocker	Read v2	G6...	[X]Cerebrovascular diseases	Antihypertensive
Beta-blocker	Read v2	Gyu6.	[X]Cerebrovascular diseases	Antihypertensive
Beta-blocker	Read v2	9h21.	Except stroke qual ind: Pt uns	Antihypertensive
Beta-blocker	Read v2	G64..	Cerebral arterial occlusion	Antihypertensive
Beta-blocker	Read v2	14A7.	H/O: CVA/stroke	Antihypertensive
Beta-blocker	Read v2	9h22.	Exc stroke qual ind: Infor dis	Antihypertensive
Beta-blocker	Read v2	8HBJ.	Stroke / TIA referral	Antihypertensive
Beta-blocker	Read v2	9Om1.	Stroke/TIA monitor 2nd letter	Antihypertensive
Beta-blocker	Read v2	8HTQ.	Referral to stroke clinic	Antihypertensive
Beta-blocker	Read v2	G64z.	Cerebral infarction NOS	Antihypertensive

Continued on next page

Table E.1 – *Continued from previous page*

Defined	System	Code	Description	Notes
Beta-blocker	Read v2	9N0p.	Seen in stroke clinic	Antihypertensive
Beta-blocker	Read v2	14AB.	H/O: TIA	Antihypertensive
Beta-blocker	Read v2	9N4X.	Did not attend stroke clinic	Antihypertensive
Beta-blocker	Read v2	9Om2.	Stroke/TIA monitor 3rd letter	Antihypertensive
Beta-blocker	Read v2	G667.	Left sided CVA	Antihypertensive
Beta-blocker	Read v2	388I.	Stroke risk	Antihypertensive
Beta-blocker	Read v2	9h2..	Except report: stroke qual ind	Antihypertensive
Beta-blocker	Read v2	G668.	Right sided CVA	Antihypertensive
Beta-blocker	Read v2	G65z.	Transient cerebral ischaem.NOS	Antihypertensive
Beta-blocker	Read v2	G65zz	Transient cerebral ischaem.NOS	Antihypertensive
Beta-blocker	Read v2	9Om..	Stroke/TIA monitoring admin	Antihypertensive
Beta-blocker	Read v2	1JA1.	Suspect cerebrovasculr disease	Antihypertensive
Beta-blocker	Read v2	9Om4.	Stroke/TIA monitr phone invite	Antihypertensive
Beta-blocker	Read v2	9Om3.	Stroke/TIA monitor verb invit	Antihypertensive
Beta-blocker	Read v2	G6z..	Cerebrovascular disease NOS	Antihypertensive
Beta-blocker	Read v2	G64z2	Left sided cerebral infarction	Antihypertensive
Beta-blocker	Read v2	G640.	Cerebral thrombosis	Antihypertensive
Beta-blocker	Read v2	6F...	Stroke prevention	Antihypertensive
Beta-blocker	Read v2	G64z3	Right sided cerebral infarct	Antihypertensive
Beta-blocker	Read v2	G63y0	Cerebr infct/throm/precere art	Antihypertensive
Beta-blocker	Read v2	G6711	Chronic cerebral ischaemia	Antihypertensive
Beta-blocker	Read v2	G663.	Brain stem stroke syndrome	Antihypertensive

Continued on next page

Table E.1 – *Continued from previous page*

Defined	System	Code	Description	Notes
Beta-blocker	Read v2	G641.	Cerebral embolism	Antihypertensive
Beta-blocker	Read v2	G664.	Cerebellar stroke syndrome	Antihypertensive
Beta-blocker	Read v2	G67..	Other cerebrovascular disease	Antihypertensive
Beta-blocker	Read v2	7A252	Embolisation cerebral art NEC	Antihypertensive
Beta-blocker	Read v2	Gyu64	[X]Other cerebral infarction	Antihypertensive
Beta-blocker	Read v2	G670.	Cerebral atherosclerosis	Antihypertensive
Beta-blocker	Read v2	1JK..	Suspected TIA	Antihypertensive
Beta-blocker	Read v2	G660.	Middle cerebral artery syndrm	Antihypertensive
Beta-blocker	Read v2	7A250	PC TL embolisation cerebr art	Antihypertensive
Beta-blocker	Read v2	1477.	H/O: cerebrovascular disease	Antihypertensive
Beta-blocker	Read v2	G6400	Cerebr infct/throm/cerebrl art	Antihypertensive
Beta-blocker	Read v2	G65y.	Other transient cerebral isch.	Antihypertensive
Beta-blocker	Read v2	1JA10	Suspct cerebrovasclar accident	Antihypertensive
Beta-blocker	Read v2	G6y..	Cerebrovascular disease OS	Antihypertensive
Beta-blocker	Read v2	Fyu55	[X]Oth cerebral TIA's+rel synd	Antihypertensive
Beta-blocker	Read v2	G63y1	Cerebr infct/embol/precere art	Antihypertensive
Beta-blocker	Read v2	G63..	Precerebral arterial occlusion	Antihypertensive
Beta-blocker	Read v2	G68X.	Seql/strok,n spc/h'm,infarc	Antihypertensive
Beta-blocker	Read v2	G65z1	Intermittent CVA	Antihypertensive
Beta-blocker	Read v2	38DM.	ABCD2 stroke risk score	Antihypertensive
Beta-blocker	Read v2	G68..	Cerebrovasc.dis.-late effects	Antihypertensive
Beta-blocker	Read v2	G6410	Cerebr infct/embol/cerebrl art	Antihypertensive

Continued on next page

Table E.1 – *Continued from previous page*

Defined	System	Code	Description	Notes
Beta-blocker	Read v2	G661.	Anterior cerebral artery syn	Antihypertensive
Beta-blocker	Read v2	G67z.	Other cerebrovasc.disease NOS	Antihypertensive
Beta-blocker	Read v2	14AK.	H/O: Stroke in last year	Antihypertensive
Beta-blocker	Read v2	7A246	Open embolisation cerebral art	Antihypertensive
Beta-blocker	Read v2	G63z.	Precerebral artery occlus. NOS	Antihypertensive
Beta-blocker	Read v2	G683.	Sequelae/cerebral infarction	Antihypertensive
Beta-blocker	Read v2	G63y.	Other precerebral artery occl.	Antihypertensive
Beta-blocker	Read v2	8HHM.	Ref to stroke func improv serv	Antihypertensive
Beta-blocker	Read v2	1M4..	Central post-stroke pain	Antihypertensive
Beta-blocker	Read v2	Gyu66	[X]Oc+sten/o cerebral arteries	Antihypertensive
Beta-blocker	Read v2	7A244	Open embolectomy cerebral art	Antihypertensive
Beta-blocker	Read v2	G65z0	Impending CVA	Antihypertensive
Beta-blocker	Read v2	Gyu65	[X]Oc+steno/o precerebral artr	Antihypertensive
Beta-blocker	Read v2	G654.	Multi+bilat precerebrl art syn	Antihypertensive
Beta-blocker	Read v2	Gyu67	[X]Other spcfd cerebrovasc dis	Antihypertensive
Beta-blocker	Read v2	Gyu6A	[X]Oth cerebrovasc diso/dis CE	Antihypertensive
Clopidogrel	Read v2	bu51.	CLOPIDOGREL 75mg tablets	
Clopidogrel	Read v2	bu52.	PLAVIX 75mg tablets	
Clopidogrel	Read v2	bu54.	CLOPIDOGREL 300mg tablets	
Cholesterol	Read v2	44P..	Serum cholesterol	
Cholesterol	Read v2	44P3.	Serum cholesterol raised	
Cholesterol	Read v2	44PJ.	Serum total cholesterol level	

Continued on next page

Table E.1 – *Continued from previous page*

Defined	System	Code	Description	Notes
Cholesterol	Read v2	44P1.	Serum cholesterol normal	
Cholesterol	Read v2	44OE.	Plasma total cholesterol level	
Cholesterol	Read v2	44P2.	Serum cholesterol borderline	
Cholesterol	Read v2	44PZ.	Serum cholesterol NOS	
Cholesterol	Read v2	44PH.	Total cholesterol measurement	
Cholesterol	Read v2	44P4.	Serum cholesterol very high	
Cholesterol	Read v2	44P9.	Serum cholesterol studies	
Cholesterol	Read v2	44PK.	Serum fastng total cholesterol	
Cholesterol	Read v2	662a.	Pre-treatmnt serum cholest lev	
BMI	Read v2	22K..	Body Mass Index	
BMI	Read v2	22K5.	Body mass index 30+ - obesity	
BMI	Read v2	22K1.	Body Mass Index normal K/M2	
BMI	Read v2	22K2.	Body Mass Index high K/M2	
BMI	Read v2	22K4.	BMI 25-29 - overweight	
BMI	Read v2	22K8.	Body mass index 20-24 - normal	
BMI	Read v2	22K7.	BMI 40+ - severely obese	
BMI	Read v2	22K3.	Body Mass Index low K/M2	
BMI	Read v2	22K6.	Body mass index less than 20	
Blood pressure measurement	Read v2	246..	O/E - blood pressure	
Blood pressure measurement	Read v2	2464.	O/E - BP reading normal	

Continued on next page

Table E.1 – *Continued from previous page*

Defined	System	Code	Description	Notes
Blood pressure measurement	Read v2	2466.	O/E - BP reading raised	
Blood pressure measurement	Read v2	2469.	O/E - Systolic BP reading	
Blood pressure measurement	Read v2	662L.	24 hr blood pressure monitor.	
Blood pressure measurement	Read v2	2465.	O/E - BP borderline raised	
Blood pressure measurement	Read v2	246E.	Sitting blood pressure reading	
Blood pressure measurement	Read v2	246Z.	O/E-blood pressure reading NOS	
Blood pressure measurement	Read v2	246M.	White coat hypertension	
Blood pressure measurement	Read v2	246D.	Standing blood pressure reading	
Blood pressure measurement	Read v2	246W.	Ave 24h systol blood pressure	
Blood pressure measurement	Read v2	246Y.	Average day interval systolic blood pressure	
Blood pressure measurement	Read v2	246b.	Average night interval systolic blood pressure	
Blood pressure measurement	Read v2	2467.	O/E - BP reading very high	
Blood pressure measurement	Read v2	2462.	O/E - BP reading low	

Continued on next page

Table E.1 – *Continued from previous page*

Defined	System	Code	Description	Notes
Blood pressure measurement	Read v2	246N.	Standing systolic blood pressure	
Blood pressure measurement	Read v2	246C.	Lying blood pressure reading	
Blood pressure measurement	Read v2	246F.	O/E - blood pressure decreased	
Blood pressure measurement	Read v2	246V.	Ave 24h diastol blood pressure	
Blood pressure measurement	Read v2	2463.	O/E - BP borderline low	
Blood pressure measurement	Read v2	246Q.	Sitting systolic blood pressure	
Blood pressure measurement	Read v2	246X.	Ave day diastol blood pressure	
Blood pressure measurement	Read v2	246a.	Ave night diast blood pressure	
Blood pressure measurement	Read v2	246S.	Lying systolic blood pressure	
Blood pressure measurement	Read v2	246T.	Lying diastolic blood pressure	
HDL reading	Read v2	44P5.	Serum HDL cholesterol level	
HDL reading	Read v2	44PB.	Serum fast HDL cholesterol lev	
HDL reading	Read v2	44PC.	Ser random HDL cholesterol lev	
HDL reading	Read v2	44d3.	Plasma fast HDL cholest level	
HDL reading	Read v2	44R3.	Lipoprotein electroph. - HDL	

Continued on next page

Table E.1 – *Continued from previous page*

Defined	System	Code	Description	Notes
HDL reading	Read v2	44dA.	Plasma HDL cholesterol level	
HDL reading	Read v2	44d2.	Plasma rndm HDL cholest level	
Cholesterol HDL ratio reading	Read v2	44lF.	Serum cholesterol/HDL ratio	
Cholesterol HDL ratio reading	Read v2	44PF.	Total cholesterol:HDL ratio	
Cholesterol HDL ratio reading	Read v2	44l2.	Cholesterol/HDL ratio	
Cholesterol HDL ratio reading	Read v2	44lG.	Plasma cholesterol/HDL ratio	
Calcium channel blocker	Read v2	blb1.	AMLODIPINE 5mg tablets	Antihypertensive only
Calcium channel blocker	Read v2	blb2.	AMLODIPINE 10mg tablets	Antihypertensive only
Calcium channel blocker	Read v2	bl8i.	ADALAT LA 30mg m/r tablets	Antihypertensive only
Calcium channel blocker	Read v2	bl8j.	ADALAT LA 60mg m/r tablets	Antihypertensive only
Calcium channel blocker	Read v2	blh1.	LERCANIDIPINE HCL 10mg tablets	Antihypertensive only
Calcium channel blocker	Read v2	bl8M.	ADALAT LA 20mg m/r tablets	Antihypertensive only

Continued on next page

Table E.1 – *Continued from previous page*

Defined	System	Code	Description	Notes
Calcium channel blocker	Read v2	bl5Z.	TILDIEM LA 200mg m/r capsules	Antihypertensive only
Calcium channel blocker	Read v2	bl83.	ADALAT RETARD 20mg m/r tablets	Antihypertensive only
Calcium channel blocker	Read v2	ble2.	LACIDIPINE 4mg tablets	Antihypertensive only
Calcium channel blocker	Read v2	bl84.	ADALAT RETARD 10mg m/r tablets	Antihypertensive only
Calcium channel blocker	Read v2	bb3k.	SECURON SR 240mg m/r tabs 28CP	Antihypertensive only
Calcium channel blocker	Read v2	ble1.	LACIDIPINE 2mg tablets	Antihypertensive only
Calcium channel blocker	Read v2	bl5I.	ADIZEM-XL 240mg m/r capsules	Antihypertensive only
Calcium channel blocker	Read v2	bl5A.	TILDIEM LA 300mg m/r capsules	Antihypertensive only
Calcium channel blocker	Read v2	bl8A.	ADIPINE MR 20 m/r tablets	Antihypertensive only
Calcium channel blocker	Read v2	bl58.	TILDIEM RETARD 90mg m/r tabs	Antihypertensive only
Calcium channel blocker	Read v2	blb3.	ISTIN 5mg tablets	Antihypertensive only
Calcium channel blocker	Read v2	bl5K.	ADIZEM-XL 120mg m/r capsules	Antihypertensive only
Calcium channel blocker	Read v2	bb3j.	SECURON SR 240mg m/r tablets	Antihypertensive only

Continued on next page

Table E.1 – *Continued from previous page*

Defined	System	Code	Description	Notes
Calcium channel blocker	Read v2	bl8z.	NIFEDIPINE 20mg m/r tablets	Antihypertensive only
Calcium channel blocker	Read v2	bb31.	VERAPAMIL 40mg tablets	Antihypertensive only
Calcium channel blocker	Read v2	bl5E.	ADIZEM-XL 300mg m/r capsules	Antihypertensive only
Calcium channel blocker	Read v2	bl8B.	ADIPINE MR 10 m/r tablets	Antihypertensive only
Calcium channel blocker	Read v2	bl5J.	ADIZEM-XL 180mg m/r capsules	Antihypertensive only
Calcium channel blocker	Read v2	bl5B.	ADIZEM-SR 90mg m/r capsules	Antihypertensive only
Calcium channel blocker	Read v2	bl59.	TILDIEM RETARD 120mg m/r tabs	Antihypertensive only
Calcium channel blocker	Read v2	bl86.	NIFEDIPINE 10mg capsules	Antihypertensive only
Calcium channel blocker	Read v2	bl51.	TILDIEM 60mg tablets	Antihypertensive only
Calcium channel blocker	Read v2	bl5R.	ANGITIL SR 90 m/r capsules	Antihypertensive only
Calcium channel blocker	Read v2	bl8e.	CORACTEN SR 20mg m/r capsules	Antihypertensive only
Calcium channel blocker	Read v2	bb3A.	VERAPAMIL 240mg m/r tablets	Antihypertensive only
Calcium channel blocker	Read v2	bl8X.	CORACTEN XL 30mg m/r capsules	Antihypertensive only

Continued on next page

Table E.1 – *Continued from previous page*

Defined	System	Code	Description	Notes
Calcium channel blocker	Read v2	bl85.	NIFEDIPINE 5mg capsules	Antihypertensive only
Calcium channel blocker	Read v2	blb4.	ISTIN 10mg tablets	Antihypertensive only
Calcium channel blocker	Read v2	bl8w.	NIFEDIPINE 10mg m/r tablets	Antihypertensive only
Calcium channel blocker	Read v2	bl5Q.	SLOZEM 240mg m/r capsules	Antihypertensive only
Calcium channel blocker	Read v2	bl8k.	CORACTEN SR 10mg m/r capsules	Antihypertensive only
Calcium channel blocker	Read v2	bb32.	VERAPAMIL 80mg tablets	Antihypertensive only
Calcium channel blocker	Read v2	bl5C.	ADIZEM-SR 120mg m/r capsules	Antihypertensive only
Calcium channel blocker	Read v2	bb33.	VERAPAMIL 120mg tablets	Antihypertensive only
Calcium channel blocker	Read v2	bl5F.	DILZEM SR 60mg m/r capsules	Antihypertensive only
Calcium channel blocker	Read v2	bl5S.	ANGITIL SR 120 m/r capsules	Antihypertensive only
Calcium channel blocker	Read v2	bb3F.	HALF-SECURON SR 120mg 28CP	Antihypertensive only
Calcium channel blocker	Read v2	blh3.	LERCANIDIPINE HCl 20mg tablets	Antihypertensive only
Calcium channel blocker	Read v2	bb3y.	VERAPAMIL 240mg m/r capsules	Antihypertensive only

Continued on next page

Table E.1 – *Continued from previous page*

Defined	System	Code	Description	Notes
Calcium channel blocker	Read v2	bl8Y.	CORACTEN XL 60mg m/r capsules	Antihypertensive only
Calcium channel blocker	Read v2	bl5D.	ADIZEM-SR 180mg m/r capsules	Antihypertensive only
Calcium channel blocker	Read v2	bl5G.	DILZEM SR 90mg m/r capsules	Antihypertensive only
Calcium channel blocker	Read v2	bl82.	ADALAT 10mg capsules	Antihypertensive only
Calcium channel blocker	Read v2	bb3s.	VERTAB SR 240 m/r tablets	Antihypertensive only
Calcium channel blocker	Read v2	bl5O.	SLOZEM 120mg m/r capsules	Antihypertensive only
Calcium channel blocker	Read v2	ble3.	MOTENS 2mg tablets	Antihypertensive only
Calcium channel blocker	Read v2	bl5P.	SLOZEM 180mg m/r capsules	Antihypertensive only
Calcium channel blocker	Read v2	bl8u.	NIFEDIPINE 10mg m/r capsules	Antihypertensive only
Calcium channel blocker	Read v2	bl8v.	NIFEDIPINE 20mg m/r capsules	Antihypertensive only
Calcium channel blocker	Read v2	ble4.	MOTENS 4mg tablets	Antihypertensive only
Calcium channel blocker	Read v2	bb3n.	UNIVER 240mg m/r capsules x28	Antihypertensive only
Calcium channel blocker	Read v2	bl5M.	DILZEM-XL 180mg m/r capsules	Antihypertensive only

Continued on next page

Table E.1 – *Continued from previous page*

Defined	System	Code	Description	Notes
Calcium channel blocker	Read v2	bl5U.	ANGITIL SR 180 m/r capsules	Antihypertensive only
Calcium channel blocker	Read v2	bl5L.	DILZEM-XL 120mg m/r capsules	Antihypertensive only
Calcium channel blocker	Read v2	bl81.	ADALAT 5mg capsules	Antihypertensive only
Calcium channel blocker	Read v2	bb3C.	VERAPAMIL 120mg m/r tablets	Antihypertensive only
Calcium channel blocker	Read v2	bl7y.	NICARDIPINE 20mg capsules	Antihypertensive only
Calcium channel blocker	Read v2	bb3l.	UNIVER 120mg m/r capsules x28	Antihypertensive only
Calcium channel blocker	Read v2	bl54.	ADIZEM-SR 120mg m/r tablets	Antihypertensive only
Calcium channel blocker	Read v2	bl7z.	NICARDIPINE 30mg capsules	Antihypertensive only
Calcium channel blocker	Read v2	bl5x.	ANGITIL XL 240 m/r capsules	Antihypertensive only
Calcium channel blocker	Read v2	blh2.	ZANIDIP 10mg tablets	Antihypertensive only
Calcium channel blocker	Read v2	bl5N.	DILZEM-XL 240mg m/r capsules	Antihypertensive only
Calcium channel blocker	Read v2	bb3v.	VERAPAMIL 120mg m/r capsules	Antihypertensive only
Calcium channel blocker	Read v2	bl5h.	DILTIAZEM HCL 200mg m/r caps	Antihypertensive only

Continued on next page

Defined	System	Code	Description	Notes
Calcium channel blocker	Read v2	bb3B.	HALF SECURON SR 120mg m/r tabs	Antihypertensive only
Calcium channel blocker	Read v2	bl5y.	ANGITIL XL 300 m/r capsules	Antihypertensive only
Calcium channel blocker	Read v2	bl55.	DILTIAZEM HCL 120mg m/r tabs	Antihypertensive only
Calcium channel blocker	Read v2	bl71.	CARDENE 20mg capsules	Antihypertensive only
Calcium channel blocker	Read v2	bl7x.	NICARDIPINE 30mg m/r capsules	Antihypertensive only
Calcium channel blocker	Read v2	bl8L.	FORTIPINE LA40 m/r tablets	Antihypertensive only
Calcium channel blocker	Read v2	bb3w.	VERAPAMIL 160mg tablets	Antihypertensive only
Calcium channel blocker	Read v2	bl5t.	VIAZEM XL 360mg m/r capsules	Antihypertensive only
Calcium channel blocker	Read v2	bb3m.	UNIVER 180mg m/r capsules x56	Antihypertensive only
Calcium channel blocker	Read v2	bl5V.	CALCICARD CR 90mg m/r tablets	Antihypertensive only
Calcium channel blocker	Read v2	bb3g.	*SECURON 120mg tablets 56CP	Antihypertensive only
Calcium channel blocker	Read v2	bl73.	CARDENE SR 30mg m/r capsules	Antihypertensive only
Calcium channel blocker	Read v2	bl7w.	NICARDIPINE 45mg m/r capsules	Antihypertensive only

Continued on next page

Table E.1 – *Continued from previous page*

Defined	System	Code	Description	Notes
Calcium channel blocker	Read v2	bl72.	CARDENE 30mg capsules	Antihypertensive only
Calcium channel blocker	Read v2	bla1.	ISRADIPINE 2.5mg tablets	Antihypertensive only
Calcium channel blocker	Read v2	bb3z.	VERAPAMIL 180mg m/r capsules	Antihypertensive only
Calcium channel blocker	Read v2	bl8h.	*NIFENSAR XL 20mg m/r tablets	Antihypertensive only
Calcium channel blocker	Read v2	bla2.	PRESCAL 2.5mg tablets	Antihypertensive only
Calcium channel blocker	Read v2	bl74.	CARDENE SR 45mg m/r capsules	Antihypertensive only
Calcium channel blocker	Read v2	bl5W.	CALCICARD CR 120mg m/r tablets	Antihypertensive only
Calcium channel blocker	Read v2	bl8S.	NIFEDIPRESS MR 10 m/r tablets	Antihypertensive only
Calcium channel blocker	Read v2	bl8F.	NIFEDIPINE 40mg m/r tablets	Antihypertensive only
Calcium channel blocker	Read v2	bb39.	*CORDILOX 80mg tablets	Antihypertensive only
Calcium channel blocker	Read v2	bb38.	*CORDILOX 40mg tablets	Antihypertensive only
Calcium channel blocker	Read v2	bb3D.	VERAPAMIL 40mg/5mL s/f soln	Antihypertensive only
Calcium channel blocker	Read v2	bl57.	*ADIZEM 60mg tablets	Antihypertensive only

Continued on next page

Table E.1 – *Continued from previous page*

Defined	System	Code	Description	Notes
Calcium channel blocker	Read v2	bb3e.	*SECURON 80mg tablets	Antihypertensive only
Calcium channel blocker	Read v2	blh4.	ZANIDIP 20mg tablets	Antihypertensive only
Calcium channel blocker	Read v2	bl8P.	*ANGIOPINE MR 10mg m/r tablets	Antihypertensive only
Calcium channel blocker	Read v2	bl8t.	*NIFOPRESS RETRD 20mg m/r tabs	Antihypertensive only
Calcium channel blocker	Read v2	bl8D.	*NIMODREL MR 10 m/r tablets	Antihypertensive only
Calcium channel blocker	Read v2	bl8q.	HYPOLAR RETARD 20 m/r tablets	Antihypertensive only
Calcium channel blocker	Read v2	bl53.	*BRITIAZIM 60mg tablets	Antihypertensive only
Calcium channel blocker	Read v2	bb3Q.	VERA-TIL SR 120mg m/r tablets	Antihypertensive only
Calcium channel blocker	Read v2	bb3P.	VERA-TIL SR 240mg m/r tablets	Antihypertensive only
Calcium channel blocker	Read v2	dt13.	NIMODIPINE 30mg tablets	Antihypertensive only
Calcium channel blocker	Read v2	blb5.	AMLOSTIN 5mg tablets	Antihypertensive only
Calcium channel blocker	Read v2	bb3i.	*SECURON 160mg tablets	Antihypertensive only
Calcium channel blocker	Read v2	bb3x.	*VERPAMIL HCL 120mg tabs x56	Antihypertensive only

Continued on next page

Table E.1 – *Continued from previous page*

Defined	System	Code	Description	Notes
Calcium channel blocker	Read v2	blb6.	AMLOSTIN 10mg tablets	Antihypertensive only
Calcium channel blocker	Read v2	bl8C.	*UNIPINE XL 30mg m/r tablets	Antihypertensive only
Calcium channel blocker	Read v2	bl56.	*ANGIOZEM 60mg tablets	Antihypertensive only
Calcium channel blocker	Read v2	dt14.	NIMOTOP 30mg tablets	Antihypertensive only
Calcium channel blocker	Read v2	bl8R.	*GENALAT RETARD 20mg m/r tabs	Antihypertensive only
Calcium channel blocker	Read v2	bl8G.	*ANGIOPINE 40 LA m/r tablets	Antihypertensive only
Calcium channel blocker	Read v2	bb30.	SECURON IV 5mg/2mL injection	Antihypertensive only
Calcium channel blocker	Read v2	bl8O.	*SLOFEDIPINE 20mg m/r tablets	Antihypertensive only
Calcium channel blocker	Read v2	bb3h.	*SECURON 160mg tablets 56CP	Antihypertensive only
Calcium channel blocker	Read v2	bl52.	*CALCICARD 60mg tablets	Antihypertensive only
ACE inhibitor	Read v2	bi67.	RAMIPRIL 10mg capsules	Antihypertensive
ACE inhibitor	Read v2	bi63.	RAMIPRIL 5mg capsules	Antihypertensive
ACE inhibitor	Read v2	bi62.	RAMIPRIL 2.5mg capsules	Antihypertensive
ACE inhibitor	Read v2	bi34.	LISINOPRIL 20mg tablets	Antihypertensive
ACE inhibitor	Read v2	bi33.	LISINOPRIL 10mg tablets	Antihypertensive

Continued on next page

Table E.1 – *Continued from previous page*

Defined	System	Code	Description	Notes
ACE inhibitor	Read v2	bi52.	PERINDOPRIL ERBUMINE 4mg tabs	Antihypertensive
ACE inhibitor	Read v2	bi32.	LISINOPRIL 5mg tablets	Antihypertensive
ACE inhibitor	Read v2	bi51.	PERINDOPRIL ERBUMINE 2mg tabs	Antihypertensive
ACE inhibitor	Read v2	bk42.	VALSARTAN 80mg capsules	Antihypertensive
ACE inhibitor	Read v2	bi61.	RAMIPRIL 1.25mg capsules	Antihypertensive
ACE inhibitor	Read v2	bk32.	LOSARTAN POTASSIUM 50mg tabs	Antihypertensive
ACE inhibitor	Read v2	bi57.	PERINDOPRIL ERBUMINE 8mg tabs	Antihypertensive
ACE inhibitor	Read v2	bi31.	LISINOPRIL 2.5mg tablets	Antihypertensive
ACE inhibitor	Read v2	bk43.	VALSARTAN 160mg capsules	Antihypertensive
ACE inhibitor	Read v2	bk52.	IRBESARTAN 150mg tablets	Antihypertensive
ACE inhibitor	Read v2	bk37.	LOSARTAN POTASSIUM 100mg tabs	Antihypertensive
ACE inhibitor	Read v2	bi2z.	ENALAPRIL MAL 20mg tabs x28	Antihypertensive
ACE inhibitor	Read v2	bk53.	IRBESARTAN 300mg tablets	Antihypertensive
ACE inhibitor	Read v2	bk73.	CANDESARTAN CILEXETIL 8mg tabs	Antihypertensive
ACE inhibitor	Read v2	bi2x.	ENALAPRIL MAL 10mg tabs x28	Antihypertensive
ACE inhibitor	Read v2	bk41.	VALSARTAN 40mg capsules	Antihypertensive
ACE inhibitor	Read v2	bi2y.	ENALAPRIL MALEATE 20mg tablets	Antihypertensive
ACE inhibitor	Read v2	bk31.	LOSARTAN POTASSIUM 25mg tabs	Antihypertensive
ACE inhibitor	Read v2	bi2v.	ENALAPRIL MALEATE 5mg tabs x28	Antihypertensive
ACE inhibitor	Read v2	bk72.	CANDESARTAN CILEXETIL 4mg tabs	Antihypertensive
ACE inhibitor	Read v2	bi2w.	ENALAPRIL MALEATE 10mg tablets	Antihypertensive
ACE inhibitor	Read v2	bk74.	CANDESARTAN CILEXET 16mg tabs	Antihypertensive

Continued on next page

Table E.1 – *Continued from previous page*

Defined	System	Code	Description	Notes
ACE inhibitor	Read v2	bk51.	IRBESARTAN 75mg tablets	Antihypertensive
ACE inhibitor	Read v2	bi93.	TRANDOLAPRIL 2mg capsules	Antihypertensive
ACE inhibitor	Read v2	bi2u.	ENALAPRIL MALEATE 5mg tablets	Antihypertensive
ACE inhibitor	Read v2	bi72.	FOSINOPRIL 20mg tablets	Antihypertensive
ACE inhibitor	Read v2	bi1v.	CAPTOPRIL 12.5mg tablets	Antihypertensive
ACE inhibitor	Read v2	bi6D.	RAMIPRIL 5mg tablets	Antihypertensive
ACE inhibitor	Read v2	bi2t.	ENALAPRIL MALEATE 2.5mg tabs	Antihypertensive
ACE inhibitor	Read v2	bi6E.	RAMIPRIL 10mg tablets	Antihypertensive
ACE inhibitor	Read v2	bk81.	TELMISARTAN 40mg tablets	Antihypertensive
ACE inhibitor	Read v2	bi1x.	CAPTOPRIL 25mg tablets x56	Antihypertensive
ACE inhibitor	Read v2	bi6C.	RAMIPRIL 2.5mg tablets	Antihypertensive
ACE inhibitor	Read v2	bi1z.	CAPTOPRIL 50mg tablets	Antihypertensive
ACE inhibitor	Read v2	bi1w.	CAPTOPRIL 25mg tablets	Antihypertensive
ACE inhibitor	Read v2	bk82.	TELMISARTAN 80mg tablets	Antihypertensive
ACE inhibitor	Read v2	bi43.	QUINAPRIL 20mg tablets	Antihypertensive
ACE inhibitor	Read v2	bi54.	*COVERSYL 4mg tablets	Antihypertensive
ACE inhibitor	Read v2	bi71.	FOSINOPRIL 10mg tablets	Antihypertensive
ACE inhibitor	Read v2	bkB2.	OLMESARTAN MEDOXOMIL 20mg tabs	Antihypertensive
ACE inhibitor	Read v2	bkB1.	OLMESARTAN MEDOXOMIL 10mg tabs	Antihypertensive
ACE inhibitor	Read v2	bi3p.	LISINO+HYDROCHL 20/12.5mg tabs	Antihypertensive
ACE inhibitor	Read v2	bi42.	QUINAPRIL 10mg tablets	Antihypertensive
ACE inhibitor	Read v2	bi4A.	QUINAPRIL 40mg tablets	Antihypertensive

Continued on next page

Table E.1 – *Continued from previous page*

Defined	System	Code	Description	Notes
ACE inhibitor	Read v2	bk71.	CANDESARTAN CILEXETIL 2mg tabs	Antihypertensive
ACE inhibitor	Read v2	bi92.	TRANDOLAPRIL 1mg capsules	Antihypertensive
ACE inhibitor	Read v2	bk45.	DIOVAN 80mg capsules	Antihypertensive
ACE inhibitor	Read v2	bi3h.	ZESTRIL 10mg tablets	Antihypertensive
ACE inhibitor	Read v2	bk9z.	EPROSARTAN 600mg tablets	Antihypertensive
ACE inhibitor	Read v2	bk8z.	TELMISARTAN 20mg tablets	Antihypertensive
ACE inhibitor	Read v2	bi1y.	CAPTOPRIL 50mg tablets x56	Antihypertensive
ACE inhibitor	Read v2	bi53.	*COVERSYL 2mg tablets	Antihypertensive
ACE inhibitor	Read v2	bi3f.	ZESTRIL 5mg tablets	Antihypertensive
ACE inhibitor	Read v2	bi3j.	ZESTRIL 20mg tablets	Antihypertensive
ACE inhibitor	Read v2	bi3t.	LISINO+HYDROCHL 10/12.5mg tabs	Antihypertensive
ACE inhibitor	Read v2	bk34.	COZAAR 50mg tablets	Antihypertensive
ACE inhibitor	Read v2	bk7z.	CANDESARTAN CILEXETL 32mg tabs	Antihypertensive
ACE inhibitor	Read v2	bi55.	*PERIND ERB+INDAP 4/1.25mg tab	Antihypertensive
ACE inhibitor	Read v2	bi6B.	RAMIPRIL 1.25mg tablets	Antihypertensive
ACE inhibitor	Read v2	bi41.	QUINAPRIL 5mg tablets	Antihypertensive
ACE inhibitor	Read v2	bkB3.	OLMESARTAN MEDOXOMIL 40mg tabs	Antihypertensive
ACE inhibitor	Read v2	bi25.	INNOVACE 10mg tablets x28	Antihypertensive
ACE inhibitor	Read v2	bi3n.	ZESTORETIC 20/12.5mg tablets	Antihypertensive
ACE inhibitor	Read v2	bk9y.	EPROSARTAN 400mg tablets	Antihypertensive
ACE inhibitor	Read v2	bk35.	LOSART+HYDROCHLTHZ 50/12.5 tab	Antihypertensive
ACE inhibitor	Read v2	bi27.	INNOVACE 20mg tablets x28	Antihypertensive

Continued on next page

Table E.1 – *Continued from previous page*

Defined	System	Code	Description	Notes
ACE inhibitor	Read v2	bk5y.	IRBES+HYDROCHL 300/12.5mg tabs	Antihypertensive
ACE inhibitor	Read v2	bk46.	DIOVAN 160mg capsules	Antihypertensive
ACE inhibitor	Read v2	bi9z.	TRANDOLAPRIL 4mg capsules	Antihypertensive
ACE inhibitor	Read v2	bk36.	COZAAR-COMP 50mg/12.5mg tabs	Antihypertensive
ACE inhibitor	Read v2	bk9x.	EPROSARTAN 300mg tablets	Antihypertensive
ACE inhibitor	Read v2	bi1c.	*CAPOZIDE 50mg tablets x28	Antihypertensive
ACE inhibitor	Read v2	bk5z.	IRBES+HYDROCHL 150/12.5mg tabs	Antihypertensive
ACE inhibitor	Read v2	bk55.	APROVEL 150mg tablets	Antihypertensive
ACE inhibitor	Read v2	bi91.	TRANDOLAPRIL 500mcg capsules	Antihypertensive
ACE inhibitor	Read v2	bi3g.	ZESTRIL 10mg tablets 28CP	Antihypertensive
ACE inhibitor	Read v2	bi23.	INNOVACE 5mg tablets x28	Antihypertensive
ACE inhibitor	Read v2	bi3e.	ZESTRIL 5mg tablets 28CP	Antihypertensive
ACE inhibitor	Read v2	bi3d.	ZESTRIL 2.5mg tablets	Antihypertensive
ACE inhibitor	Read v2	bi74.	*STARIL 20mg tablets	Antihypertensive
ACE inhibitor	Read v2	bk4w.	VALSARTAN 40mg tablets	Antihypertensive
ACE inhibitor	Read v2	bi73.	*STARIL 10mg tablets	Antihypertensive
ACE inhibitor	Read v2	bi24.	INNOVACE 10mg tablets	Antihypertensive
ACE inhibitor	Read v2	bi56.	*COVERSYL PLUS 4mg/1.25mg tabs	Antihypertensive
ACE inhibitor	Read v2	bk58.	COAPROVEL 300mg/12.5mg tablets	Antihypertensive
ACE inhibitor	Read v2	bi96.	GOPTEN 2mg capsules	Antihypertensive
ACE inhibitor	Read v2	bi26.	INNOVACE 20mg tablets	Antihypertensive
ACE inhibitor	Read v2	bi22.	INNOVACE 5mg tablets	Antihypertensive

Continued on next page

Table E.1 – *Continued from previous page*

Defined	System	Code	Description	Notes
ACE inhibitor	Read v2	bi18.	CAPOTEN 25mg tablets x56	Antihypertensive
ACE inhibitor	Read v2	bi16.	*CAPOTEN 12.5mg tablets	Antihypertensive
ACE inhibitor	Read v2	bi28.	INNOZIDE 20/12.5mg tablets	Antihypertensive
ACE inhibitor	Read v2	bk44.	DIOVAN 40mg capsules	Antihypertensive
ACE inhibitor	Read v2	bi17.	CAPOTEN 25mg tablets	Antihypertensive
ACE inhibitor	Read v2	bi45.	ACCUPRO 10mg tablets 28CP	Antihypertensive
ACE inhibitor	Read v2	bk4z.	VALSRT+HYDROCHL 160/12.5mg tab	Antihypertensive
ACE inhibitor	Read v2	bi3i.	ZESTRIL 20mg tablets 28CP	Antihypertensive
ACE inhibitor	Read v2	bk8y.	TELMIS+HYDROCHL 80/12.5mg tabs	Antihypertensive
ACE inhibitor	Read v2	bi69.	*RAMIPRIL 2.5mg+5mg+10mg caps	Antihypertensive
ACE inhibitor	Read v2	bi66.	*TRITACE 5mg capsules	Antihypertensive
ACE inhibitor	Read v2	bk3z.	LOSART+HYDROCHLTHZ 100/25 tabs	Antihypertensive
ACE inhibitor	Read v2	bi1G.	*CO-ZIDOCAPT 50mg/25mg tablets	Antihypertensive
ACE inhibitor	Read v2	bk56.	APROVEL 300mg tablets	Antihypertensive
ACE inhibitor	Read v2	bk4x.	VALSART+HYDROCHL 80/12.5mg tab	Antihypertensive
ACE inhibitor	Read v2	bk77.	AMIAS 8mg tablets	Antihypertensive
ACE inhibitor	Read v2	bi1a.	CAPOTEN 50mg tablets x56	Antihypertensive
ACE inhibitor	Read v2	bi44.	ACCUPRO 5mg tablets 28CP	Antihypertensive
ACE inhibitor	Read v2	bk57.	COAPROVEL 150mg/12.5mg tablets	Antihypertensive
ACE inhibitor	Read v2	bi46.	ACCUPRO 20mg tablets 28CP	Antihypertensive
ACE inhibitor	Read v2	bi1s.	CAPOZIDE 50mg/25mg tablets	Antihypertensive
ACE inhibitor	Read v2	bk54.	APROVEL 75mg tablets	Antihypertensive

Continued on next page

Table E.1 – *Continued from previous page*

Defined	System	Code	Description	Notes
ACE inhibitor	Read v2	bk76.	AMIAS 4mg tablets	Antihypertensive
ACE inhibitor	Read v2	bi2b.	ENALAP+HYDROCHL 20/12.5mg tabs	Antihypertensive
ACE inhibitor	Read v2	bi65.	*TRITACE 2.5mg capsules	Antihypertensive
ACE inhibitor	Read v2	biC8.	PERIND ARG+INDAP 5/1.25mg tabs	Antihypertensive
ACE inhibitor	Read v2	biBz.	IMIDAPRIL HCL 10mg tablets	Antihypertensive
ACE inhibitor	Read v2	bi3s.	ZESTORETIC 10/12.5mg tablets	Antihypertensive
ACE inhibitor	Read v2	bk4y.	VALSART+HYDROCHL 160/25mg tabs	Antihypertensive
ACE inhibitor	Read v2	bk33.	COZAAR HALF-STRENGTH 25mg tabs	Antihypertensive
ACE inhibitor	Read v2	bkB4.	OLMETEC 10mg tablets	Antihypertensive
ACE inhibitor	Read v2	bi3m.	*ZESTORETIC tablets 28CP	Antihypertensive
ACE inhibitor	Read v2	bk8x.	TELMIS+HYDROCHL 40/12.5mg tabs	Antihypertensive
ACE inhibitor	Read v2	bi21.	INNOVACE 2.5mg tablets	Antihypertensive
ACE inhibitor	Read v2	bi39.	*CARACE 10mg tablets	Antihypertensive
ACE inhibitor	Read v2	bi95.	GOPTEN 1mg capsules	Antihypertensive
ACE inhibitor	Read v2	bi3k.	CARACE 20 PLUS tablets	Antihypertensive
ACE inhibitor	Read v2	bk83.	MICARDIS 40mg tablets	Antihypertensive
ACE inhibitor	Read v2	bk78.	AMIAS 16mg tablets	Antihypertensive
ACE inhibitor	Read v2	bk87.	MICARDISPLUS 80mg/12.5mg tabs	Antihypertensive
ACE inhibitor	Read v2	bi47.	ACCURETIC tablets	Antihypertensive
ACE inhibitor	Read v2	bi3b.	*CARACE 20mg tablets	Antihypertensive
ACE inhibitor	Read v2	bi3q.	*ZESTRIL 2.5mg starter pack	Antihypertensive
ACE inhibitor	Read v2	bi64.	*TRITACE 1.25mg capsules	Antihypertensive

Continued on next page

Table E.1 – *Continued from previous page*

Defined	System	Code	Description	Notes
ACE inhibitor	Read v2	bk38.	COZAAR 100mg tablets	Antihypertensive
ACE inhibitor	Read v2	bi49.	ACCUPRO 40mg tablets	Antihypertensive
ACE inhibitor	Read v2	bi35.	*CARACE 2.5mg tablets	Antihypertensive
ACE inhibitor	Read v2	bi8a.	CILAZAPRIL 5mg tablets	Antihypertensive
ACE inhibitor	Read v2	bi38.	*CARACE 10mg tablets 28CP	Antihypertensive
ACE inhibitor	Read v2	bk4v.	VALSARTAN 320mg tablets	Antihypertensive
ACE inhibitor	Read v2	bi19.	CAPOTEN 50mg tablets	Antihypertensive
ACE inhibitor	Read v2	bi3c.	ZESTRIL 2.5mg tablets 28CP	Antihypertensive
ACE inhibitor	Read v2	bi36.	*CARACE 5mg tablets 28CP	Antihypertensive
ACE inhibitor	Read v2	bkD1.	AMLODIPNE+VALSARTN 5/80mg tabs	Antihypertensive
ACE inhibitor	Read v2	bi68.	*TRITACE 10mg capsules	Antihypertensive
ACE inhibitor	Read v2	bi37.	*CARACE 5mg tablets	Antihypertensive
ACE inhibitor	Read v2	bkFy.	ALISKIREN 300mg tablets	Antihypertensive
ACE inhibitor	Read v2	bi58.	*COVERSYL 8mg tablets	Antihypertensive
ACE inhibitor	Read v2	biBy.	IMIDAPRIL HCL 5mg tablets	Antihypertensive
ACE inhibitor	Read v2	bk62.	TARKA 2mg/180mg m/r capsules	Antihypertensive
ACE inhibitor	Read v2	bi3a.	*CARACE 20mg tablets 28CP	Antihypertensive
ACE inhibitor	Read v2	bk84.	MICARDIS 80mg tablets	Antihypertensive
ACE inhibitor	Read v2	bk93.	TEVETEN 600mg tablets	Antihypertensive
ACE inhibitor	Read v2	bi3l.	CARACE 10 PLUS tablets	Antihypertensive
ACE inhibitor	Read v2	bk47.	CO-DIOVAN 160mg/12.5mg tablets	Antihypertensive
ACE inhibitor	Read v2	bk49.	CO-DIOVAN 80mg/12.5mg tablets	Antihypertensive

Continued on next page

Table E.1 – *Continued from previous page*

Defined	System	Code	Description	Notes
ACE inhibitor	Read v2	bA1z.	FELODIPINE+RAMIPRIL 5/5mg tabs	Antihypertensive
ACE inhibitor	Read v2	bkD2.	AMLODPNE+VALSARTN 5/160mg tabs	Antihypertensive
ACE inhibitor	Read v2	bi13.	ACEPRIL 25mg tablets x56	Antihypertensive
ACE inhibitor	Read v2	bkB5.	OLMETEC 20mg tablets	Antihypertensive
ACE inhibitor	Read v2	bi2a.	*ENALAPRIL MAL tabs titre pack	Antihypertensive
ACE inhibitor	Read v2	bi15.	ACEPRIL 50mg tablets x56	Antihypertensive
ACE inhibitor	Read v2	bi6x.	TRITACE 5mg tablets	Antihypertensive
ACE inhibitor	Read v2	bA12.	TRIAPIN 5mg/5mg tablets	Antihypertensive
ACE inhibitor	Read v2	bk86.	MICARDISPLUS 40mg/12.5mg tabs	Antihypertensive
ACE inhibitor	Read v2	bkCz.	OLMESAR+HYDROCH 20/12.5mg tabs	Antihypertensive
ACE inhibitor	Read v2	bi6w.	TRITACE 10mg tablets	Antihypertensive
ACE inhibitor	Read v2	bkD3.	AMLODPNE+VALSRTN 10/160mg tabs	Antihypertensive
ACE inhibitor	Read v2	bi6y.	TRITACE 2.5mg tablets	Antihypertensive
ACE inhibitor	Read v2	biBx.	IMIDAPRIL HCL 20mg tablets	Antihypertensive
ACE inhibitor	Read v2	bi3r.	*LISINOPRIL 2.5mg tabs starter	Antihypertensive
ACE inhibitor	Read v2	bk75.	AMIAS 2mg tablets	Antihypertensive
ACE inhibitor	Read v2	bk3C.	LOSARTAN POTASSIUM 12.5mg tabs	Antihypertensive
ACE inhibitor	Read v2	bi11.	ACEPRIL 12.5mg tablets	Antihypertensive
ACE inhibitor	Read v2	bi82.	CILAZAPRIL 500mcg tablets	Antihypertensive
ACE inhibitor	Read v2	bi94.	GOPTEN 500micrograms capsules	Antihypertensive
ACE inhibitor	Read v2	bk39.	COZAAR-COMP 100mg/25mg tablets	Antihypertensive
ACE inhibitor	Read v2	bi89.	VASCACE 5mg tablets	Antihypertensive

Continued on next page

Table E.1 – *Continued from previous page*

Defined	System	Code	Description	Notes
ACE inhibitor	Read v2	bk48.	CO-DIOVAN 160mg/25mg tablets	Antihypertensive
ACE inhibitor	Read v2	bk85.	MICARDIS 20mg tablets	Antihypertensive
ACE inhibitor	Read v2	bi88.	VASCACE 2.5mg tablets	Antihypertensive
ACE inhibitor	Read v2	biC7.	COVRSYL ARGIN PLS 5/1.25mg tab	Antihypertensive
ACE inhibitor	Read v2	bi48.	QUINAPRIL+HYDROCHLOROTHIAZIDE	Antihypertensive
ACE inhibitor	Read v2	bi1d.	CAPOZIDE LS 25mg tablets x28CP	Antihypertensive
ACE inhibitor	Read v2	biC2.	PERINDOPRIL ARGININE 2.5mg tab	Antihypertensive
ACE inhibitor	Read v2	biC4.	PERINDOPRIL ARGININE 5mg tabs	Antihypertensive
ACE inhibitor	Read v2	biC6.	PERINDOPRIL ARGININE 10mg tabs	Antihypertensive
ACE inhibitor	Read v2	bi1b.	ACEZIDE 50mg tablets x56	Antihypertensive
ACE inhibitor	Read v2	bi87.	VASCACE 1mg tablets	Antihypertensive
ACE inhibitor	Read v2	bkDx.	EXFORGE 10mg/160mg tablets	Antihypertensive
ACE inhibitor	Read v2	bi84.	CILAZAPRIL 2.5mg tablets	Antihypertensive
ACE inhibitor	Read v2	bk3y.	LOSART+HYDRCHL 100/12.5mg tabs	Antihypertensive
ACE inhibitor	Read v2	bi6A.	*TRITACE Titration Pack caps	Antihypertensive
ACE inhibitor	Read v2	bA1y.	FELODIP+RAMIPRL 2.5/2.5mg tabs	Antihypertensive
ACE inhibitor	Read v2	biA1.	MOEXIPRIL HCL 7.5mg tablets	Antihypertensive
ACE inhibitor	Read v2	bk91.	TEVETEN 300mg tablets	Antihypertensive
ACE inhibitor	Read v2	bi1f.	CAPTOP+HYDROCHL 50/25mg tabs	Antihypertensive
ACE inhibitor	Read v2	bkDy.	EXFORGE 5mg/160mg tablets	Antihypertensive
ACE inhibitor	Read v2	bi12.	ACEPRIL 25mg tablets	Antihypertensive
ACE inhibitor	Read v2	bi6F.	RAMIPRIL 2.5+5+10mg tabs pack	Antihypertensive

Continued on next page

Table E.1 – *Continued from previous page*

Defined	System	Code	Description	Notes
ACE inhibitor	Read v2	bk5x.	IRBES+HYDROCHL 300mg/25mg tabs	Antihypertensive
ACE inhibitor	Read v2	bk92.	TEVETEN 400mg tablets	Antihypertensive
ACE inhibitor	Read v2	bkDz.	EXFORGE 5mg/80mg tablets	Antihypertensive
ACE inhibitor	Read v2	bi6z.	TRITACE 1.25mg tablets	Antihypertensive
ACE inhibitor	Read v2	biB1.	TANATRIL 5mg tablets	Antihypertensive
ACE inhibitor	Read v2	bkCy.	OLMESART+HYDROCHL 20/25mg tabs	Antihypertensive
ACE inhibitor	Read v2	bi83.	CILAZAPRIL 1mg tablets	Antihypertensive
ACE inhibitor	Read v2	biB2.	TANATRIL 10mg tablets	Antihypertensive
ACE inhibitor	Read v2	bk61.	TRANDOL+VERAP 2/180mg m/r caps	Antihypertensive
ACE inhibitor	Read v2	bi86.	VASCACE 500micrograms tablets	Antihypertensive
ACE inhibitor	Read v2	biA3.	PERDIX 7.5mg tablets	Antihypertensive
ACE inhibitor	Read v2	bi14.	ACEPRIL 50mg tablets	Antihypertensive
ACE inhibitor	Read v2	bkB6.	OLMETEC 40mg tablets	Antihypertensive
ACE inhibitor	Read v2	bkC1.	OLMETEC PLUS 20mg/12.5mg tabs	Antihypertensive
ACE inhibitor	Read v2	bk8w.	TELMIS+HYDROCHL 80mg/25mg tabs	Antihypertensive
ACE inhibitor	Read v2	biA2.	MOEXIPRIL HCL 15mg tablets	Antihypertensive
ACE inhibitor	Read v2	biC3.	COVERSYL ARGININE 5mg tablets	Antihypertensive
ACE inhibitor	Read v2	bk4A.	DIOVAN 40mg tablets	Antihypertensive
ACE inhibitor	Read v2	bi9A.	GOPTEN 4mg capsules	Antihypertensive
ACE inhibitor	Read v2	bkHz.	OLMESART+AMLODIPNE 20/5mg tabs	Antihypertensive
ACE inhibitor	Read v2	bk59.	COAPROVEL 300mg/25mg tablets	Antihypertensive
ACE inhibitor	Read v2	biC5.	COVERSYL ARGININE 10mg tablets	Antihypertensive

Continued on next page

Table E.1 – *Continued from previous page*

Defined	System	Code	Description	Notes
ACE inhibitor	Read v2	bkHy.	OLMESART+AMLODIPNE 40/5mg tabs	Antihypertensive
ACE inhibitor	Read v2	bA11.	TRIAPIN MITE 2.5mg/2.5mg tabs	Antihypertensive
ACE inhibitor	Read v2	biA4.	PERDIX 15mg tablets	Antihypertensive
ACE inhibitor	Read v2	bi1p.	*TENSOPRIL 12.5mg tablets	Antihypertensive
ACE inhibitor	Read v2	bk3A.	COZAAR-COMP 100mg/12.5mg tabs	Antihypertensive
ACE inhibitor	Read v2	bi6o.	TRITACE Titration Pack tablets	Antihypertensive
ACE inhibitor	Read v2	bkHx.	OLMESART+AMLODIPN 40/10mg tabs	Antihypertensive
ACE inhibitor	Read v2	bk79.	AMIAS 32mg tablets	Antihypertensive
ACE inhibitor	Read v2	bi6v.	*LOPACE 10mg capsules	Antihypertensive
ACE inhibitor	Read v2	bkH3.	SEVIKAR 40mg/10mg tablets	Antihypertensive
ACE inhibitor	Read v2	bk88.	MICARDISPLUS 80mg/25mg tablets	Antihypertensive
ACE inhibitor	Read v2	bi2F.	*INNOVACE MELT 5mg wafer	Antihypertensive
ACE inhibitor	Read v2	bi2G.	*INNOVACE MELT 10mg wafer	Antihypertensive
ACE inhibitor	Read v2	bkH2.	SEVIKAR 40mg/5mg tablets	Antihypertensive
ACE inhibitor	Read v2	bkH1.	SEVIKAR 20mg/5mg tablets	Antihypertensive
ACE inhibitor	Read v2	bkC3.	OLMETEC PLUS 40mg/12.5mg tabs	Antihypertensive
ACE inhibitor	Read v2	bi2D.	*ENALAPRIL MALEATE 20mg wafer	Antihypertensive
ACE inhibitor	Read v2	biC1.	COVERSYL ARGININE 2.5mg tabs	Antihypertensive
ACE inhibitor	Read v2	bi2L.	*PRALENAL 10mg tablets	Antihypertensive
ACE inhibitor	Read v2	bi81.	*CILAZAPRIL 250mcg tablets	Antihypertensive
ACE inhibitor	Read v2	bi2E.	*INNOVACE MELT 2.5mg wafer	Antihypertensive
ACE inhibitor	Read v2	bi6u.	*LOPACE 5mg capsules	Antihypertensive

Continued on next page

Table E.1 – *Continued from previous page*

Defined	System	Code	Description	Notes
ACE inhibitor	Read v2	bi29.	*INNOVACE tabs titration pack	Antihypertensive
ACE inhibitor	Read v2	bi2M.	*PRALENAL 20mg tablets	Antihypertensive
ACE inhibitor	Read v2	bi2C.	*ENALAPRIL MALEATE 10mg wafer	Antihypertensive
ACE inhibitor	Read v2	bi1q.	*TENSOPRIL 25mg tablets	Antihypertensive
ACE inhibitor	Read v2	bkCx.	OLMESAR+HYDROCH 40/12.5mg tabs	Antihypertensive
ACE inhibitor	Read v2	bi4D.	QUINIL 20mg tablets	Antihypertensive
ACE inhibitor	Read v2	biB3.	TANATRIL 20mg tablets	Antihypertensive
ACE inhibitor	Read v2	bi1k.	*KAPLON 25mg tablets	Antihypertensive
ACE inhibitor	Read v2	bi1j.	*KAPLON 12.5mg tablets	Antihypertensive
ACE inhibitor	Read v2	bi2K.	*PRALENAL 5mg tablets	Antihypertensive
ACE inhibitor	Read v2	bi6t.	*LOPACE 2.5mg capsules	Antihypertensive
ACE inhibitor	Read v2	bi2J.	*PRALENAL 2.5mg tablets	Antihypertensive
ACE inhibitor	Read v2	bkGy.	AMBRISENTAN 10mg tablets	Antihypertensive
ACE inhibitor	Read v2	bk3E.	LOSARTAN POTASS 2.5mg/mL susp	Antihypertensive
ACE inhibitor	Read v2	bkGz.	AMBRISENTAN 5mg tablets	Antihypertensive
Thiazide diuretic	Read v2	b211.	BENDROFLUMETHIAZIDE 2.5mg tabs	Antihypertensive only
Thiazide diuretic	Read v2	b212.	BENDROFLUMETHIAZIDE 5mg tablet	Antihypertensive only
Thiazide diuretic	Read v2	b28z.	INDAPAMIDE 2.5mg tablets	Antihypertensive only
Thiazide diuretic	Read v2	b285.	INDAPAMIDE 1.5mg m/r tablets	Antihypertensive only

Continued on next page

Table E.1 – *Continued from previous page*

Defined	System	Code	Description	Notes
Thiazide diuretic	Read v2	b281.	NATRILIX 2.5mg tablets	Antihypertensive only
Thiazide diuretic	Read v2	b286.	NATRILIX SR 1.5mg m/r tablets	Antihypertensive only
Thiazide diuretic	Read v2	b2bz.	METOLAZONE 5mg tablets	Antihypertensive only
Thiazide diuretic	Read v2	b25z.	CYCLOPENTHIAZIDE 500mcg tabs	Antihypertensive only
Thiazide diuretic	Read v2	b23y.	CHLORTALIDONE 50mg tablets	Antihypertensive only
Thiazide diuretic	Read v2	b2d1.	DIUREXAN 20mg tablets	Antihypertensive only
Thiazide diuretic	Read v2	b2dz.	XIPAMIDE 20mg tablets	Antihypertensive only
Thiazide diuretic	Read v2	b214.	APRINOX 5mg tablets	Antihypertensive only
Thiazide diuretic	Read v2	b231.	HYGROTON 50mg tablets	Antihypertensive only
Thiazide diuretic	Read v2	b251.	NAVIDREX 500micrograms tablets	Antihypertensive only
Thiazide diuretic	Read v2	b26z.	*HYDROCHLOROTHIAZIDE 25mg tabs	Antihypertensive only
Thiazide diuretic	Read v2	b213.	APRINOX 2.5mg tablets	Antihypertensive only
Thiazide diuretic	Read v2	b26y.	*HYDROCHLOROTHIAZIDE 50mg tabs	Antihypertensive only

Continued on next page

Table E.1 – *Continued from previous page*

Defined	System	Code	Description	Notes
Thiazide diuretic	Read v2	b219.	NEO-NACLEX 5mg tablets	Antihypertensive only
Thiazide diuretic	Read v2	b2b1.	METENIX-5 5mg tablets	Antihypertensive only
Thiazide diuretic	Read v2	b283.	*NATRAMID 2.5mg tablets	Antihypertensive only
Thiazide diuretic	Read v2	b2c1.	*NEPHRIL 1mg tablets	Antihypertensive only
Thiazide diuretic	Read v2	b2b2.	*XURET 500micrograms tablets	Antihypertensive only
Thiazide diuretic	Read v2	b263.	*HYDROSALURIC 25mg tablets	Antihypertensive only
Thiazide diuretic	Read v2	b216.	*BERKOZIDE 5mg tablets	Antihypertensive only
Thiazide diuretic	Read v2	b232.	*HYGROTON 100mg tablets	Antihypertensive only
Thiazide diuretic	Read v2	b22y.	CHLOROTHIAZIDE 250mg/5mL susp	Antihypertensive only
Thiazide diuretic	Read v2	b291.	*BAYCARON 25mg tablets	Antihypertensive only
Thiazide diuretic	Read v2	b22z.	*CHLOROTHIAZIDE 500mg tablets	Antihypertensive only
Thiazide diuretic	Read v2	b215.	*BERKOZIDE 2.5mg tablets	Antihypertensive only
Thiazide diuretic	Read v2	b221.	*SALURIC 500mg tablets	Antihypertensive only

Continued on next page

Table E.1 – *Continued from previous page*

Defined	System	Code	Description	Notes
Thiazide diuretic	Read v2	b2cz.	*POLYTHIAZIDE 1mg tablets	Antihypertensive only
Thiazide diuretic	Read v2	b264.	*HYDROSALURIC 50mg tablets	Antihypertensive only
Thiazide diuretic	Read v2	b21A.	*NEO-BENDROMAX 2.5mg tablets	Antihypertensive only
Thiazide diuretic	Read v2	b23z.	*CHLORTHALIDONE 100mg tablets	Antihypertensive only
Thiazide diuretic	Read v2	b2b3.	*METOLAZONE 500mcg tablets	Antihypertensive only
Thiazide diuretic	Read v2	b29z.	*MEFRUSIDE 25mg tablets	Antihypertensive only
Thiazide diuretic	Read v2	b271.	*HYDRENOX 50mg tablets	Antihypertensive only
Thiazide diuretic	Read v2	b21B.	*NEO-BENDROMAX 5mg tablets	Antihypertensive only
Thiazide diuretic	Read v2	b218.	*CENTYL 5mg tablets	Antihypertensive only
Thiazide diuretic	Read v2	b217.	*CENTYL 2.5mg tablets	Antihypertensive only
Aspirin	Read v2	bu23.	ASPIRIN 75mg disp tabs	
Aspirin	Read v2	bu25.	*ASPIRIN 75mg tablets	
Aspirin	Read v2	bu2B.	ASPIRIN 75mg e/c tablets	
Aspirin	Read v2	di1f.	ASPIRIN 300mg e/c tablets	
Aspirin	Read v2	di13.	*ASPIRIN 75mg disp tabs	

Continued on next page

Table E.1 – *Continued from previous page*

Defined	System	Code	Description	Notes
Aspirin	Read v2	j112.	ASPIRIN 300mg disp tablets	
Aspirin	Read v2	j111.	ASPIRIN 300mg tablets	
Aspirin	Read v2	bu2A.	NU-SEALS ASPIRIN 75mg e/c tabs	
Aspirin	Read v2	di1m.	ASPIRIN 300mg soluble tablets	
Aspirin	Read v2	bu27.	*ASPIRIN 300mg eff tabs	
Aspirin	Read v2	bu2c.	ASPIRIN 75mg soluble tablets	
Aspirin	Read v2	di1c.	NU-SEALS ASPIRIN 300mg e/ctabs	
Aspirin	Read v2	di11.	ASPIRIN [CNS] 300mg tablets	
Aspirin	Read v2	bu2F.	CAPRIN 75mg e/c tablets	
Aspirin	Read v2	bu28.	*DISPRIN CV 100mg m/r tablets	
Aspirin	Read v2	bu29.	*ASPIRIN 100mg m/r tablets	
Aspirin	Read v2	di12.	ASPIRIN [CNS] 300mg disp tabs	
Aspirin	Read v2	bu2E.	*POSTMI 75mg e/c tablets	
Aspirin	Read v2	bu2K.	MICROPIRIN 75mg e/c tablets	
Aspirin	Read v2	bu2G.	*NU-SEALS CARDIO 75 e/c tabs	
Aspirin	Read v2	di1g.	*ASPIRIN 600mg e/c tablets	
Aspirin	Read v2	bu21.	*ASPIRIN 100mg eff tabs	
Aspirin	Read v2	di1h.	*ASPIRIN 324mg e/c tablets	
Aspirin	Read v2	di1r.	DISPRIN 300mg disp tabs	
Aspirin	Read v2	bu24.	*ANGETTES 75mg tablets	
Aspirin	Read v2	di1k.	CAPRIN 300mg e/c tablets	
Aspirin	Read v2	di1e.	*PALAPRIN FORTE 600mg tablets	

Continued on next page

Table E.1 – *Continued from previous page*

Defined	System	Code	Description	Notes
Aspirin	Read v2	di1d.	*NU-SEALS ASPIRIN 600mg tabs	
Aspirin	Read v2	di1o.	ASPIRIN 150mg suppositories	
Aspirin	Read v2	di1n.	ASPIRIN 300mg suppositories	
Aspirin	Read v2	bu2H.	*ENPRIN 75mg e/c tablets	
Aspirin	Read v2	bu2I.	ASPIRIN 162.5mg m/r capsules	
Aspirin	Read v2	di14.	*ASPERGUM 227mg chewing gum	
Aspirin	Read v2	di18.	*SOLPRIN 300mg disp tabs	
CABG	OPCS	K401	SAPHENOUS VEIN GRAFT REPLACEMENT OF CORONARY ARTERY	
CABG	OPCS	K402	SAPHENOUS VEIN GRAFT REPLACEMENT OF CORONARY ARTERY	
CABG	OPCS	K403	SAPHENOUS VEIN GRAFT REPLACEMENT OF CORONARY ARTERY	
CABG	OPCS	K404	SAPHENOUS VEIN GRAFT REPLACEMENT OF CORONARY ARTERY	
CABG	OPCS	K408	SAPHENOUS VEIN GRAFT REPLACEMENT OF CORONARY ARTERY	
CABG	OPCS	K409	SAPHENOUS VEIN GRAFT REPLACEMENT OF CORONARY ARTERY	
CABG	OPCS	K411	OTHER AUTOGRAFT REPLACEMENT OF CORONARY ARTERY	
CABG	OPCS	K412	OTHER AUTOGRAFT REPLACEMENT OF CORONARY ARTERY	
CABG	OPCS	K413	OTHER AUTOGRAFT REPLACEMENT OF CORONARY ARTERY	

Continued on next page

Table E.1 – *Continued from previous page*

Defined	System	Code	Description	Notes
CABG	OPCS	K414	OTHER AUTOGRAFT REPLACEMENT OF CORONARY ARTERY	
CABG	OPCS	K419	OTHER AUTOGRAFT REPLACEMENT OF CORONARY ARTERY	
CABG	OPCS	K421	ALLOGRAFT REPLACEMENT OF CORONARY ARTERY	
CABG	OPCS	K423	ALLOGRAFT REPLACEMENT OF CORONARY ARTERY	
CABG	OPCS	K424	ALLOGRAFT REPLACEMENT OF CORONARY ARTERY	
CABG	OPCS	K429	ALLOGRAFT REPLACEMENT OF CORONARY ARTERY	
CABG	OPCS	K431	PROSTHETIC REPLACEMENT OF CORONARY ARTERY	
CABG	OPCS	K433	PROSTHETIC REPLACEMENT OF CORONARY ARTERY	
CABG	OPCS	K434	PROSTHETIC REPLACEMENT OF CORONARY ARTERY	
CABG	OPCS	K441	OTHER REPLACEMENT OF CORONARY ARTERY	
CABG	OPCS	K442	OTHER REPLACEMENT OF CORONARY ARTERY	
CABG	OPCS	K448	OTHER REPLACEMENT OF CORONARY ARTERY	
CABG	OPCS	K449	OTHER REPLACEMENT OF CORONARY ARTERY	
CABG	OPCS	K451	CONNECTION OF THORACIC ARTERY TO CORONARY ARTERY	
CABG	OPCS	K452	CONNECTION OF THORACIC ARTERY TO CORONARY ARTERY	
CABG	OPCS	K453	CONNECTION OF THORACIC ARTERY TO CORONARY ARTERY	
CABG	OPCS	K454	CONNECTION OF THORACIC ARTERY TO CORONARY ARTERY	

Continued on next page

Table E.1 – *Continued from previous page*

Defined	System	Code	Description	Notes
CABG	OPCS	K455	CONNECTION OF THORACIC ARTERY TO CORONARY ARTERY	
CABG	OPCS	K456	CONNECTION OF THORACIC ARTERY TO CORONARY ARTERY	
CABG	OPCS	K458	CONNECTION OF THORACIC ARTERY TO CORONARY ARTERY	
CABG	OPCS	K459	CONNECTION OF THORACIC ARTERY TO CORONARY ARTERY	
CABG	OPCS	K463	OTHER BYPASS OF CORONARY ARTERY	
CABG	OPCS	K468	OTHER BYPASS OF CORONARY ARTERY	
CABG	OPCS	K473	REPAIR OF CORONARY ARTERY	
CABG	OPCS	K475	REPAIR OF CORONARY ARTERY	
CABG	OPCS	K478	REPAIR OF CORONARY ARTERY	
CABG	OPCS	K479	REPAIR OF CORONARY ARTERY	
CABG	OPCS	K482	OTHER OPEN OPERATIONS ON CORONARY ARTERY	
CABG	OPCS	K484	OTHER OPEN OPERATIONS ON CORONARY ARTERY	
CABG	OPCS	K488	OTHER OPEN OPERATIONS ON CORONARY ARTERY	
PCI	OPCS	K491	TRANSLUMINAL BALLOON ANGIOPLASTY OF CORONARY ARTERY	
PCI	OPCS	K492	TRANSLUMINAL BALLOON ANGIOPLASTY OF CORONARY ARTERY	
PCI	OPCS	K493	TRANSLUMINAL BALLOON ANGIOPLASTY OF CORONARY ARTERY	
PCI	OPCS	K494	TRANSLUMINAL BALLOON ANGIOPLASTY OF CORONARY ARTERY	

Continued on next page

Table E.1 – *Continued from previous page*

Defined	System	Code	Description	Notes
PCI	OPCS	K498	TRANSLUMINAL BALLOON ANGIOPLASTY OF CORONARY ARTERY	
PCI	OPCS	K499	TRANSLUMINAL BALLOON ANGIOPLASTY OF CORONARY ARTERY	
PCI	OPCS	K501	OTHER THERAPEUTIC TRANSLUMINAL OPERATIONS ON CORONARY	
PCI	OPCS	K502	OTHER THERAPEUTIC TRANSLUMINAL OPERATIONS ON CORONARY	
PCI	OPCS	K503	OTHER THERAPEUTIC TRANSLUMINAL OPERATIONS ON CORONARY	
PCI	OPCS	K508	OTHER THERAPEUTIC TRANSLUMINAL OPERATIONS ON CORONARY	
PCI	OPCS	K509	OTHER THERAPEUTIC TRANSLUMINAL OPERATIONS ON CORONARY	
PCI	OPCS	K751	PERCUTANEOUS TRANSLUMINAL BALLOON ANGIOPLASTY AND INSER	
PCI	OPCS	K752	PERCUTANEOUS TRANSLUMINAL BALLOON ANGIOPLASTY AND INSER	
PCI	OPCS	K753	PERCUTANEOUS TRANSLUMINAL BALLOON ANGIOPLASTY AND INSER	
PCI	OPCS	K754	PERCUTANEOUS TRANSLUMINAL BALLOON ANGIOPLASTY AND INSER	
PCI	OPCS	K758	PERCUTANEOUS TRANSLUMINAL BALLOON ANGIOPLASTY AND INSER	
PCI	OPCS	K759	PERCUTANEOUS TRANSLUMINAL BALLOON ANGIOPLASTY AND INSER	
Charlson	ICD-10	F00	Dementia in Alzheimer disease	

Continued on next page

Table E.1 – *Continued from previous page*

Defined	System	Code	Description	Notes
Charlson	ICD-10	F01	Vascular dementia	
Charlson	ICD-10	F02	Dementia in other diseases classified elsewhere	
Charlson	ICD-10	F03	Unspecified dementia	
Charlson	ICD-10	F051	Delirium superimposed on dementia	
Charlson	ICD-10	G30	Alzheimer disease	
Charlson	ICD-10	G311	Senile degeneration of brain, not elsewhere classified	
Charlson	ICD-10	I278	Other specified pulmonary heart diseases	
Charlson	ICD-10	I279	Pulmonary heart disease, unspecified	
Charlson	ICD-10	J40	Bronchitis, not specified as acute or chronic	
Charlson	ICD-10	J41	Simple and mucopurulent chronic bronchitis	
Charlson	ICD-10	J42	Unspecified chronic bronchitis	
Charlson	ICD-10	J43	Emphysema	
Charlson	ICD-10	J44	Other chronic obstructive pulmonary disease	
Charlson	ICD-10	J45	Asthma	
Charlson	ICD-10	J46	Status asthmaticus	
Charlson	ICD-10	J47	Bronchiectasis	
Charlson	ICD-10	J60	Coalworker pneumoconiosis	
Charlson	ICD-10	J61	Pneumoconiosis due to asbestos and other mineral fibres	
Charlson	ICD-10	J62	Pneumoconiosis due to dust containing silica	
Charlson	ICD-10	J63	Pneumoconiosis due to other inorganic dusts	
Charlson	ICD-10	J64	Unspecified pneumoconiosis	
Charlson	ICD-10	J65	Pneumoconiosis associated with tuberculosis	

Continued on next page

Table E.1 – *Continued from previous page*

Defined	System	Code	Description	Notes
Charlson	ICD-10	J66	Airway disease due to specific organic dust	
Charlson	ICD-10	J67	Hypersensitivity pneumonitis due to organic dust	
Charlson	ICD-10	J684	Chronic respiratory conditions due to chemicals, gases, fumes and vapours	
Charlson	ICD-10	J701	Chronic and other pulmonary manifestations due to radiation	
Charlson	ICD-10	J703	Chronic drug-induced interstitial lung disorders	
Charlson	ICD-10	M05	Seropositive rheumatoid arthritis	
Charlson	ICD-10	M06	Other rheumatoid arthritis	
Charlson	ICD-10	M315	Giant cell arteritis with polymyalgia rheumatica	
Charlson	ICD-10	M32	Systemic lupus erythematosus	
Charlson	ICD-10	M33	Dermatopolymyositis	
Charlson	ICD-10	M34	Systemic sclerosis	
Charlson	ICD-10	M351	Other overlap syndromes	
Charlson	ICD-10	M353	Polymyalgia rheumatica	
Charlson	ICD-10	M360	Dermato(poly)myositis in neoplastic disease	
Charlson	ICD-10	K25	Gastric ulcer	
Charlson	ICD-10	K26	Duodenal ulcer	
Charlson	ICD-10	K27	Peptic ulcer, site unspecified	
Charlson	ICD-10	K28	Gastrojejunal ulcer	
Charlson	ICD-10	B18	Chronic viral hepatitis	
Charlson	ICD-10	K700	Alcoholic fatty liver	

Continued on next page

Table E.1 – *Continued from previous page*

Defined	System	Code	Description	Notes
Charlson	ICD-10	K701	Alcoholic hepatitis	
Charlson	ICD-10	K702	Alcoholic fibrosis and sclerosis of liver	
Charlson	ICD-10	K703	Alcoholic cirrhosis of liver	
Charlson	ICD-10	K709	Alcoholic liver disease, unspecified	
Charlson	ICD-10	K713	Toxic liver disease with chronic persistent hepatitis	
Charlson	ICD-10	K714	Toxic liver disease with chronic lobular hepatitis	
Charlson	ICD-10	K715	Toxic liver disease with chronic active hepatitis	
Charlson	ICD-10	K717	Toxic liver disease with fibrosis and cirrhosis of liver	
Charlson	ICD-10	K73	Chronic hepatitis, not elsewhere classified	
Charlson	ICD-10	K74	Fibrosis and cirrhosis of liver	
Charlson	ICD-10	K760	Fatty (change of) liver, not elsewhere classified	
Charlson	ICD-10	K762	Central haemorrhagic necrosis of liver	
Charlson	ICD-10	K763	Infarction of liver	
Charlson	ICD-10	K744	Secondary biliary cirrhosis	
Charlson	ICD-10	K768	Other specified diseases of liver	
Charlson	ICD-10	K769	Liver disease, unspecified	
Charlson	ICD-10	Z944	Liver transplant status	
Charlson	ICD-10	G041	Tropical spastic paraplegia	
Charlson	ICD-10	G114	Hereditary spastic paraplegia	
Charlson	ICD-10	G801	Spastic diplegic cerebral palsy	
Charlson	ICD-10	G802	Spastic hemiplegic cerebral palsy	
Charlson	ICD-10	G81	Hemiplegia	

Continued on next page

Table E.1 – *Continued from previous page*

Defined	System	Code	Description	Notes
Charlson	ICD-10	G82	Paraplegia and tetraplegia	
Charlson	ICD-10	G830	Diplegia of upper limbs	
Charlson	ICD-10	G831	Monoplegia of lower limb	
Charlson	ICD-10	G832	Monoplegia of upper limb	
Charlson	ICD-10	G833	Monoplegia, unspecified	
Charlson	ICD-10	G834	Cauda equina syndrome	
Charlson	ICD-10	G839	Paralytic syndrome, unspecified	
Charlson	ICD-10	I120	Hypertensive renal disease with renal failure	
Charlson	ICD-10	I131	Hypertensive heart and renal disease with renal failure	
Charlson	ICD-10	N032	Chronic nephritic syndrome: Diffuse membranous glomerulonephritis	
Charlson	ICD-10	N033	Chronic nephritic syndrome: Diffuse mesangial proliferative glomerulonephritis	
Charlson	ICD-10	N034	Chronic nephritic syndrome: Diffuse endocapillary proliferative glomerulonephritis	
Charlson	ICD-10	N035	Chronic nephritic syndrome: Diffuse mesangiocapillary glomerulonephritis	
Charlson	ICD-10	N036	Chronic nephritic syndrome: Dense deposit disease	
Charlson	ICD-10	N037	Chronic nephritic syndrome: Diffuse crescentic glomerulonephritis	
Charlson	ICD-10	N052	Unspecified nephritic syndrome: Diffuse membranous glomerulonephritis	
Charlson	ICD-10	N053	Unspecified nephritic syndrome: Diffuse mesangial proliferative glomerulonephritis	

Continued on next page

Table E.1 – *Continued from previous page*

Defined	System	Code	Description	Notes
Charlson	ICD-10	N054	Unspecified nephritic syndrome: Diffuse endocapillary proliferative glomerulonephritis	
Charlson	ICD-10	N055	Unspecified nephritic syndrome: Diffuse mesangiocapillary glomerulonephritis	
Charlson	ICD-10	N056	Unspecified nephritic syndrome: Dense deposit disease	
Charlson	ICD-10	N057	Unspecified nephritic syndrome: Diffuse crescentic glomerulonephritis	
Charlson	ICD-10	N18	Chronic kidney disease	
Charlson	ICD-10	N19	Unspecified kidney failure	
Charlson	ICD-10	N250	Renal osteodystrophy	
Charlson	ICD-10	Z490	Preparatory care for dialysis	
Charlson	ICD-10	Z491	Extracorporeal dialysis	
Charlson	ICD-10	Z492	Other dialysis	
Charlson	ICD-10	Z940	Kidney transplant status	
Charlson	ICD-10	Z992	Dependence on renal dialysis	
Charlson	ICD-10	C00	Malignant neoplasm of lip	
Charlson	ICD-10	C01	Malignant neoplasm of base of tongue	
Charlson	ICD-10	C02	Malignant neoplasm of other and unspecified parts of tongue	
Charlson	ICD-10	C03	Malignant neoplasm of gum	
Charlson	ICD-10	C04	Malignant neoplasm of floor of mouth	
Charlson	ICD-10	C05	Malignant neoplasm of palate	
Charlson	ICD-10	C06	Malignant neoplasm of other and unspecified parts of mouth	

Continued on next page

Table E.1 – *Continued from previous page*

Defined	System	Code	Description	Notes
Charlson	ICD-10	C07	Malignant neoplasm of parotid gland	
Charlson	ICD-10	C08	Malignant neoplasm of other and unspecified major salivary glands	
Charlson	ICD-10	C09	Malignant neoplasm of tonsil	
Charlson	ICD-10	C10	Malignant neoplasm of oropharynx	
Charlson	ICD-10	C11	Malignant neoplasm of nasopharynx	
Charlson	ICD-10	C12	Malignant neoplasm of piriform sinus	
Charlson	ICD-10	C13	Malignant neoplasm of hypopharynx	
Charlson	ICD-10	C14	Malignant neoplasm of other and ill-defined sites in the lip, oral cavity and pharynx	
Charlson	ICD-10	C15	Malignant neoplasm of oesophagus	
Charlson	ICD-10	C16	Malignant neoplasm of stomach	
Charlson	ICD-10	C17	Malignant neoplasm of small intestine	
Charlson	ICD-10	C18	Malignant neoplasm of colon	
Charlson	ICD-10	C19	Malignant neoplasm of rectosigmoid junction	
Charlson	ICD-10	C20	Malignant neoplasm of rectum	
Charlson	ICD-10	C21	Malignant neoplasm of anus and anal canal	
Charlson	ICD-10	C22	Malignant neoplasm of liver and intrahepatic bile ducts	
Charlson	ICD-10	C23	Malignant neoplasm of gallbladder	
Charlson	ICD-10	C24	Malignant neoplasm of other and unspecified parts of biliary tract	
Charlson	ICD-10	C25	Malignant neoplasm of pancreas	

Continued on next page

Table E.1 – *Continued from previous page*

Defined	System	Code	Description	Notes
Charlson	ICD-10	C26	Malignant neoplasm of other and ill-defined digestive organs	
Charlson	ICD-10	C30	Malignant neoplasm of nasal cavity and middle ear	
Charlson	ICD-10	C31	Malignant neoplasm of accessory sinuses	
Charlson	ICD-10	C32	Malignant neoplasm of larynx	
Charlson	ICD-10	C33	Malignant neoplasm of trachea	
Charlson	ICD-10	C34	Malignant neoplasm of bronchus and lung	
Charlson	ICD-10	C37	Malignant neoplasm of thymus	
Charlson	ICD-10	C38	Malignant neoplasm of heart, mediastinum and pleura	
Charlson	ICD-10	C39	Malignant neoplasm of other and ill-defined sites in the respiratory system and intrathoracic organs	
Charlson	ICD-10	C40	Malignant neoplasm of bone and articular cartilage of limbs	
Charlson	ICD-10	C41	Malignant neoplasm of bone and articular cartilage of other and unspecified sites	
Charlson	ICD-10	C43	Malignant melanoma of skin	
Charlson	ICD-10	C45	Mesothelioma	
Charlson	ICD-10	C46	Kaposi sarcoma	
Charlson	ICD-10	C47	Malignant neoplasm of peripheral nerves and autonomic nervous system	
Charlson	ICD-10	C48	Malignant neoplasm of retroperitoneum and peritoneum	
Charlson	ICD-10	C49	Malignant neoplasm of other connective and soft tissue	
Charlson	ICD-10	C50	Malignant neoplasm of breast	
Charlson	ICD-10	C51	Malignant neoplasm of vulva	

Continued on next page

Table E.1 – *Continued from previous page*

Defined	System	Code	Description	Notes
Charlson	ICD-10	C52	Malignant neoplasm of vagina	
Charlson	ICD-10	C53	Malignant neoplasm of cervix uteri	
Charlson	ICD-10	C54	Malignant neoplasm of corpus uteri	
Charlson	ICD-10	C55	Malignant neoplasm of uterus, part unspecified	
Charlson	ICD-10	C56	Malignant neoplasm of ovary	
Charlson	ICD-10	C57	Malignant neoplasm of other and unspecified female genital organs	
Charlson	ICD-10	C58	Malignant neoplasm of placenta	
Charlson	ICD-10	C60	Malignant neoplasm of penis	
Charlson	ICD-10	C61	Malignant neoplasm of prostate	
Charlson	ICD-10	C62	Malignant neoplasm of testis	
Charlson	ICD-10	C63	Malignant neoplasm of other and unspecified male genital organs	
Charlson	ICD-10	C64	Malignant neoplasm of kidney, except renal pelvis	
Charlson	ICD-10	C65	Malignant neoplasm of renal pelvis	
Charlson	ICD-10	C66	Malignant neoplasm of ureter	
Charlson	ICD-10	C67	Malignant neoplasm of bladder	
Charlson	ICD-10	C68	Malignant neoplasm of other and unspecified urinary organs	
Charlson	ICD-10	C69	Malignant neoplasm of eye and adnexa	
Charlson	ICD-10	C70	Malignant neoplasm of meninges	
Charlson	ICD-10	C71	Malignant neoplasm of brain	

Continued on next page

Table E.1 – *Continued from previous page*

Defined	System	Code	Description	Notes
Charlson	ICD-10	C72	Malignant neoplasm of spinal cord, cranial nerves and other parts of central nervous system	
Charlson	ICD-10	C73	Malignant neoplasm of thyroid gland	
Charlson	ICD-10	C74	Malignant neoplasm of adrenal gland	
Charlson	ICD-10	C75	Malignant neoplasm of other endocrine glands and related structures	
Charlson	ICD-10	C76	Malignant neoplasm of other and ill-defined sites	
Charlson	ICD-10	C81	Hodgkin lymphoma	
Charlson	ICD-10	C82	Follicular lymphoma	
Charlson	ICD-10	C83	Non-follicular lymphoma	
Charlson	ICD-10	C84	Mature T/NK-cell lymphomas	
Charlson	ICD-10	C85	Other and unspecified types of non-Hodgkin lymphoma	
Charlson	ICD-10	C88	Malignant immunoproliferative diseases	
Charlson	ICD-10	C90	Multiple myeloma and malignant plasma cell neoplasms	
Charlson	ICD-10	C91	Lymphoid leukaemia	
Charlson	ICD-10	C92	Myeloid leukaemia	
Charlson	ICD-10	C93	Monocytic leukaemia	
Charlson	ICD-10	C94	Other leukaemias of specified cell type	
Charlson	ICD-10	C95	Leukaemia of unspecified cell type	
Charlson	ICD-10	C96	Other and unspecified malignant neoplasms of lymphoid, haematopoietic and related tissue	
Charlson	ICD-10	C97	Malignant neoplasms of independent (primary) multiple sites	

Continued on next page

Table E.1 – *Continued from previous page*

Defined	System	Code	Description	Notes
Charlson	ICD-10	I850	Oesophageal varices with bleeding	
Charlson	ICD-10	I859	Oesophageal varices without bleeding	
Charlson	ICD-10	I864	Gastric varices	
Charlson	ICD-10	I982	Oesophageal varices without bleeding in diseases classified elsewhere	
Charlson	ICD-10	K704	Alcoholic hepatic failure	
Charlson	ICD-10	K711	Toxic liver disease with hepatic necrosis	
Charlson	ICD-10	K721	Chronic hepatic failure	
Charlson	ICD-10	K729	Hepatic failure, unspecified	
Charlson	ICD-10	K765	Hepatic veno-occlusive disease	
Charlson	ICD-10	K766	Portal hypertension	
Charlson	ICD-10	K767	Hepatorenal syndrome	
Charlson	ICD-10	C77	Secondary and unspecified malignant neoplasm of lymph nodes	
Charlson	ICD-10	C78	Secondary malignant neoplasm of respiratory and digestive organs	
Charlson	ICD-10	C79	Secondary malignant neoplasm of other and unspecified sites	
Charlson	ICD-10	C80	Malignant neoplasm, without specification of site	
Charlson	ICD-10	B20	Human immunodeficiency virus [HIV] disease resulting in infectious and parasitic diseases	
Charlson	ICD-10	B21	Human immunodeficiency virus [HIV] disease resulting in malignant neoplasms	

Continued on next page

Table E.1 – *Continued from previous page*

Defined	System	Code	Description	Notes
Charlson	ICD-10	B22	Human immunodeficiency virus [HIV] disease resulting in other specified diseases	
Charlson	ICD-10	B24	Unspecified human immunodeficiency virus [HIV] disease	
