# Supplementary material for: Equity in healthcare for coronary heart disease, Wales (UK) 2004–2010: A population-based electronic cohort study
Source: PLoS One. 2017 Mar 16;12(3):e0172618. doi: 10.1371/journal.pone.0172618 (PMC5354260; doi:10.1371/journal.pone.0172618)
Supplement: S2 File — (PDF) [file pone.0172618.s002.pdf]

S2: Covariates and outputs from cox models

The outputs from the Cox models with random effect terms used to investigate inequity across a pathway of care for coronary heart disease are shown on subsequent pages.

The figures are taken from the thesis on which this paper is based. Further information on the way these codes were employed in the full thesis can be obtained at: http://orca.cf.ac.uk/73460/

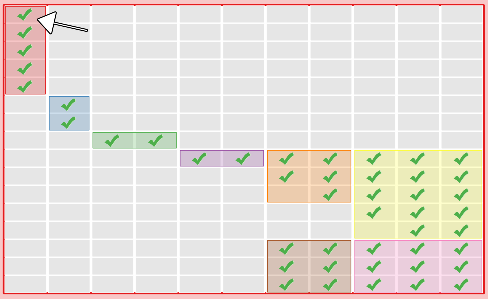

Mixed−effects model for 'aged 40+ with no high risk diagnosis' and 'ascertainment of smoking status'. Incident clinical trigger

	HR	95% CI
Quintile 1	1	(Reference)
Quintile 2	1.02	(1.00; 1.05)
Quintile 3	1.09	(1.06; 1.12)
Quintile 4	1.13	(1.10; 1.16)
Quintile 5	1.20	(1.17; 1.24)
Male	1	(Reference)
Female	1.51	(1.49; 1.54)
No hyp.	1	(Reference)
Hyp. contr.	1.86	(1.79; 1.93)
Hyp. uncontr.	1.87	(1.75; 2.00)
Untreat. hyp.	1.28	(1.22; 1.36)
No oth. co.	1	(Reference)
Other co.	1.65	(1.59; 1.70)

Number of clinical triggers 122486; Number of clinical actions 72291. ICC for practice = 0.041. Missing values imputed using MICE.

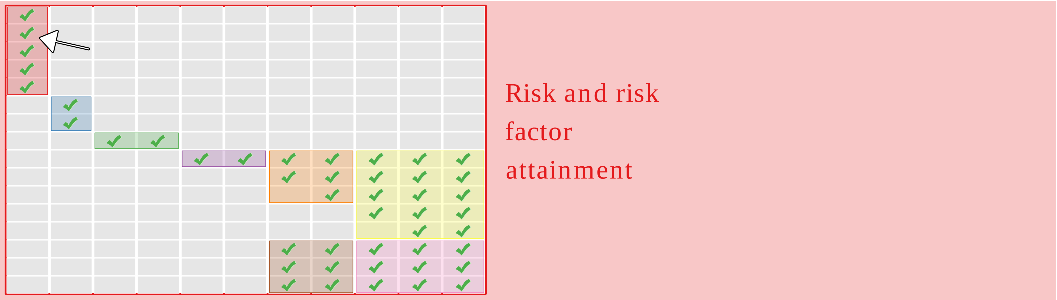

Mixed−effects model for 'aged 40+ with no high risk diagnosis' and 'measurement of BMI'. Incident clinical trigger

	HR	95% CI
Quintile 1	1	(Reference)
Quintile 2	1.04	(1.01; 1.08)
Quintile 3	1.09	(1.05; 1.12)
Quintile 4	1.08	(1.04; 1.12)
Quintile 5	1.12	(1.08; 1.16)
Male	1	(Reference)
Female	1.91	(1.88; 1.95)
No hyp.	1	(Reference)
Hyp. contr.	2.39	(2.29; 2.49)
Hyp. uncontr.	2.62	(2.43; 2.82)
Untreat. hyp.	1.61	(1.51; 1.72)
No oth. co.	1	(Reference)
Other co.	1.58	(1.52; 1.65)

Number of clinical triggers 122486; Number of clinical actions 46235. ICC for practice = 0.133. Missing values imputed using MICE.

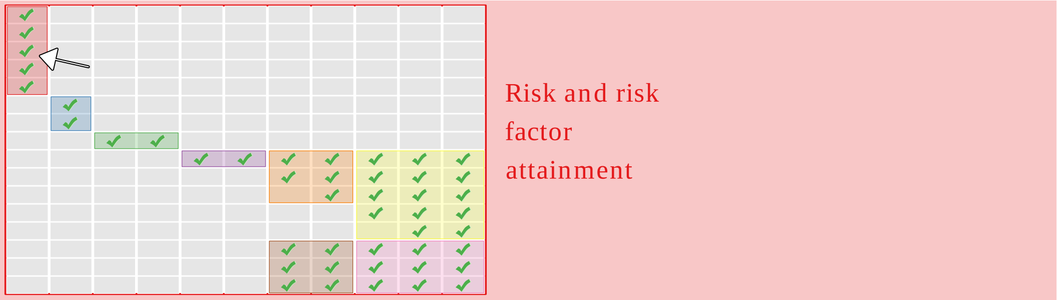

Mixed−effects model for 'aged 40+ with no high risk diagnosis' and 'measurement of BP'. Incident clinical trigger

	HR	95% CI
Quintile 1	1	(Reference)
Quintile 2	1.00	(0.97; 1.03)
Quintile 3	1.03	(1.00; 1.06)
Quintile 4	1.02	(0.99; 1.05)
Quintile 5	1.03	(1.00; 1.06)
Male	1	(Reference)
Female	1.92	(1.89; 1.95)
No hyp.	1	(Reference)
Hyp. contr.	3.88	(3.74; 4.02)
Hyp. uncontr.	5.18	(4.86; 5.51)
Untreat. hyp.	1.65	(1.57; 1.75)
No oth. co.	1	(Reference)
Other co.	1.38	(1.33; 1.43)

Number of clinical triggers 122486; Number of clinical actions 64312. ICC for practice = 0.125. Missing values imputed using MICE.

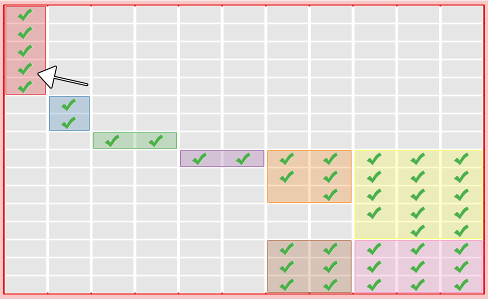

Mixed−effects model for 'aged 40+ with no high risk diagnosis' and 'measurement of cholesterol'. Incident clinical trigger

	HR	95% CI
Quintile 1	1	(Reference)
Quintile 2	0.95	(0.91; 0.99)
Quintile 3	1.01	(0.97; 1.05)
Quintile 4	0.97	(0.93; 1.02)
Quintile 5	0.97	(0.93; 1.01)
Male	1	(Reference)
Female	1.00	(0.98; 1.02)
No hyp.	1	(Reference)
Hyp. contr.	4.62	(4.42; 4.83)
Hyp. uncontr.	5.03	(4.66; 5.42)
Untreat. hyp.	2.16	(2.02; 2.32)
No oth. co.	1	(Reference)
Other co.	1.23	(1.17; 1.30)

Number of clinical triggers 122486; Number of clinical actions 28652. ICC for practice = 0.055. Missing values imputed using MICE.

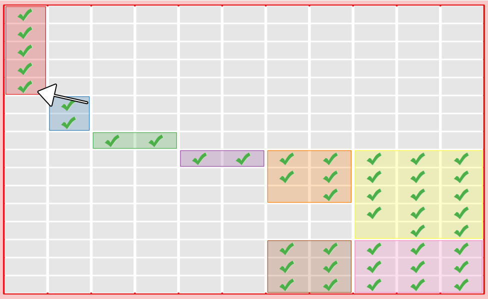

Mixed−effects model for 'aged 40+ with no high risk diagnosis' and 'full cardiovascular risk assessment'. Incident clinical trigger

	HR	95% CI
Quintile 1	1	(Reference)
Quintile 2	1.00	(0.98; 1.03)
Quintile 3	1.00	(0.97; 1.02)
Quintile 4	0.98	(0.95; 1.00)
Quintile 5	0.97	(0.95; 1.00)
Male	1	(Reference)
Female	2.21	(2.18; 2.24)
No hyp.	1	(Reference)
Hyp. contr.	1.96	(1.89; 2.02)
Hyp. uncontr.	2.24	(2.11; 2.37)
Untreat. hyp.	2.22	(2.12; 2.32)
No oth. co.	1	(Reference)
Other co.	1.38	(1.34; 1.42)

Number of clinical triggers 122486; Number of clinical actions 84969. ICC for practice = 0.166. Missing values imputed using MICE.

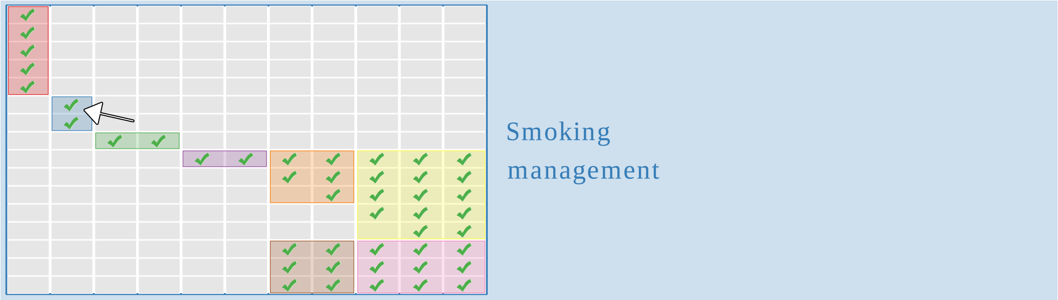

Mixed−effects model for 'first identified as smoker' and 'provision of smoking−cessation advice'. Incident clinical trigger

	HR	95% CI
Quintile 1	1	(Reference)
Quintile 2	1.02	(0.99; 1.06)
Quintile 3	1.05	(1.02; 1.09)
Quintile 4	1.08	(1.05; 1.12)
Quintile 5	1.10	(1.06; 1.14)
Age 35 to 39	0.96	(0.92; 0.99)
Age 40 to 44	0.98	(0.95; 1.02)
Age 45 to 49	1.02	(0.99; 1.06)
Age 50 to 54	1	(Reference)
Age 55 to 59	1.01	(0.98; 1.05)
Age 60 to 64	1.01	(0.97; 1.05)
Age 65 to 69	0.98	(0.94; 1.03)
Age 70 to 74	0.93	(0.88; 0.98)
Age 75 to 79	0.92	(0.86; 0.97)
Age 80 to 84	0.82	(0.77; 0.89)
Age 85+	0.80	(0.73; 0.88)
Male	1	(Reference)
Female	1.06	(1.03; 1.08)
BMI low / norm.	1	(Reference)
Overweight	0.99	(0.96; 1.01)
Obese	1.01	(0.98; 1.04)
No hyp.	1	(Reference)
Hyp. contr.	1.19	(1.15; 1.23)
Hyp. uncontr.	1.22	(1.16; 1.28)
Untreat. hyp.	1.02	(0.97; 1.08)
Chol:HDL < 4	1	(Reference)
Chol:HDL >= 4	1.08	(1.03; 1.13)
No CVA	1	(Reference)
CVA	1.10	(1.04; 1.17)
No oth. co.	1	(Reference)
Other co.	1.06	(1.02; 1.10)
No diabetes	1	(Reference)
Diabetes	1.03	(0.98; 1.08)

Number of clinical triggers 55161; Number of clinical actions 45926. ICC for practice = 0.056. Missing values imputed using MICE.

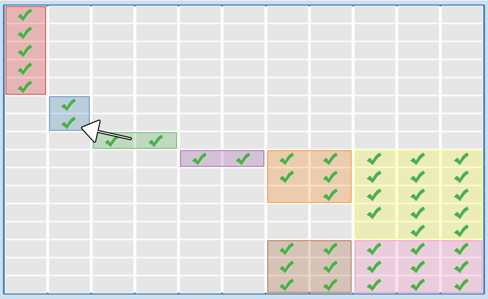

Mixed−effects model for 'first identified as smoker' and 'referral to smoking−cessation services'. Incident clinical trigger

	HR	95% CI
Quintile 1	1	(Reference)
Quintile 2	1.06	(0.90; 1.23)
Quintile 3	1.10	(0.93; 1.29)
Quintile 4	1.14	(0.97; 1.33)
Quintile 5	1.17	(0.99; 1.38)
Age 35 to 39	0.97	(0.85; 1.12)
Age 40 to 44	1.02	(0.89; 1.18)
Age 45 to 49	0.98	(0.85; 1.13)
Age 50 to 54	1	(Reference)
Age 55 to 59	0.99	(0.85; 1.15)
Age 60 to 64	1.01	(0.85; 1.19)
Age 65 to 69	0.99	(0.81; 1.20)
Age 70 to 74	0.66	(0.52; 0.85)
Age 75 to 79	0.41	(0.29; 0.58)
Age 80 to 84	0.17	(0.09; 0.33)
Age 85+	0.13	(0.05; 0.34)
Male	1	(Reference)
Female	1.12	(1.02; 1.23)
BMI low / norm.	1	(Reference)
Overweight	0.99	(0.90; 1.10)
Obese	1.05	(0.93; 1.19)
No hyp.	1	(Reference)
Hyp. contr.	1.43	(1.25; 1.64)
Hyp. uncontr.	1.27	(1.03; 1.57)
Untreat. hyp.	1.10	(0.86; 1.40)
Chol:HDL < 4	1	(Reference)
Chol:HDL >= 4	1.11	(0.75; 1.65)
No CVA	1	(Reference)
CVA	1.02	(0.77; 1.37)
No oth. co.	1	(Reference)
Other co.	1.46	(1.26; 1.69)
No diabetes	1	(Reference)
Diabetes	1.20	(0.99; 1.46)

Number of clinical triggers 55161; Number of clinical actions 2514. ICC for practice = 0.533. Missing values imputed using MICE.

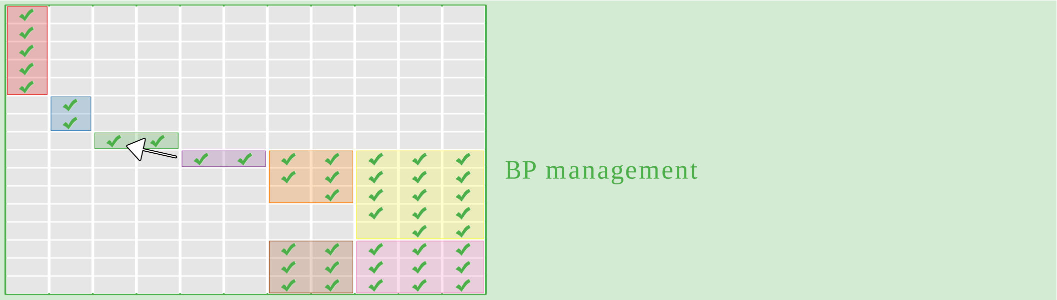

Mixed−effects model for 'BP raised and low−risk' and 'treatment with antihypertensive medication'. Incident clinical trigger

	HR	95% CI
Quintile 1	1	(Reference)
Quintile 2	1.08	(1.00; 1.15)
Quintile 3	1.14	(1.06; 1.22)
Quintile 4	1.11	(1.04; 1.20)
Quintile 5	1.22	(1.13; 1.31)
Age 35 to 39	0.96	(0.87; 1.06)
Age 40 to 44	1.16	(1.07; 1.25)
Age 45 to 49	1.09	(1.01; 1.17)
Age 50 to 54	1	(Reference)
Age 55 to 59	0.89	(0.82; 0.95)
Age 60 to 64	0.80	(0.74; 0.87)
Age 65 to 69	0.95	(0.87; 1.05)
Age 70 to 74	1.08	(0.98; 1.19)
Age 75 to 79	1.09	(0.99; 1.20)
Age 80 to 84	1.15	(1.04; 1.28)
Age 85+	0.93	(0.83; 1.05)
Male	1	(Reference)
Female	0.95	(0.90; 0.99)
BMI low / norm.	1	(Reference)
Overweight	1.03	(0.97; 1.09)
Obese	1.03	(0.96; 1.11)
Chol:HDL < 4	1	(Reference)
Chol:HDL >= 4	1.08	(0.99; 1.17)
No oth. co.	1	(Reference)
Other co.	1.08	(1.00; 1.17)

Number of clinical triggers 13814; Number of clinical actions 9899. ICC for practice = 0.052. Missing values imputed using MICE.

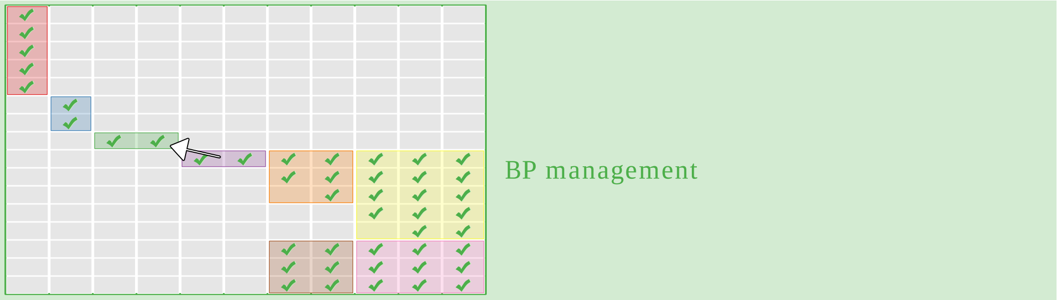

Mixed−effects model for 'BP raised and high−risk' and 'treatment with antihypertensive medication'. Incident clinical trigger

	HR	95% CI
Quintile 1	1	(Reference)
Quintile 2	1.01	(0.99; 1.04)
Quintile 3	1.00	(0.97; 1.02)
Quintile 4	1.00	(0.97; 1.03)
Quintile 5	1.00	(0.98; 1.03)
Age 35 to 39	0.88	(0.81; 0.96)
Age 40 to 44	1.09	(1.03; 1.15)
Age 45 to 49	1.06	(1.02; 1.10)
Age 50 to 54	1	(Reference)
Age 55 to 59	0.83	(0.80; 0.85)
Age 60 to 64	0.71	(0.69; 0.73)
Age 65 to 69	0.64	(0.62; 0.65)
Age 70 to 74	0.60	(0.58; 0.62)
Age 75 to 79	0.57	(0.55; 0.59)
Age 80 to 84	0.55	(0.53; 0.57)
Age 85+	0.51	(0.49; 0.53)
Male	1	(Reference)
Female	1.30	(1.28; 1.32)
BMI low / norm.	1	(Reference)
Overweight	1.10	(1.08; 1.12)
Obese	1.17	(1.15; 1.19)
Chol:HDL < 4	1	(Reference)
Chol:HDL >= 4	0.97	(0.96; 0.99)
No oth. co.	1	(Reference)
Other co.	0.95	(0.93; 0.97)
Indication 1	1	(Reference)
Indication 2	1.93	(1.85; 2.00)
Indic. years	1.01	(1.01; 1.02)

Number of clinical triggers 106079; Number of clinical actions 75797. ICC for practice = 0.046. Missing values imputed using MICE.

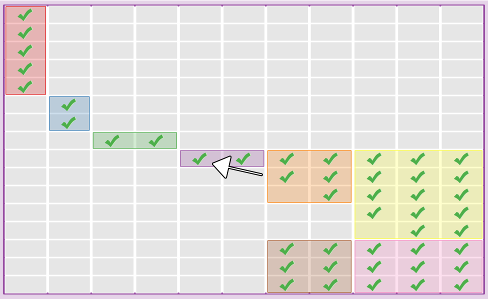

Mixed−effects model for 'risk assessed high' and 'statin'. Incident clinical trigger

	HR	95% CI
Quintile 1	1	(Reference)
Quintile 2	1.01	(0.96; 1.06)
Quintile 3	0.99	(0.94; 1.04)
Quintile 4	1.01	(0.96; 1.06)
Quintile 5	1.01	(0.95; 1.07)
Age 35 to 39	0.77	(0.58; 1.03)
Age 40 to 44	0.94	(0.84; 1.06)
Age 45 to 49	0.90	(0.84; 0.96)
Age 50 to 54	1	(Reference)
Age 55 to 59	1.20	(1.14; 1.26)
Age 60 to 64	1.30	(1.24; 1.37)
Age 65 to 69	1.31	(1.24; 1.39)
Age 70 to 74	1.19	(1.12; 1.27)
Age 75 to 79	0.82	(0.75; 0.88)
Age 80 to 84	0.56	(0.51; 0.62)
Age 85+	0.34	(0.29; 0.40)
Male	1	(Reference)
Female	1.28	(1.24; 1.32)
Non−smoker	1	(Reference)
Smoker	1.00	(0.96; 1.03)
BMI low / norm.	1	(Reference)
Overweight	1.05	(1.01; 1.09)
Obese	0.92	(0.88; 0.96)
No hyp.	1	(Reference)
Hyp. contr.	1.80	(1.73; 1.88)
Hyp. uncontr.	1.79	(1.71; 1.87)
Untreat. hyp.	1.43	(1.36; 1.49)
Chol:HDL < 4	1	(Reference)
Chol:HDL >= 4	2.35	(2.21; 2.51)
No oth. co.	1	(Reference)
Other co.	0.90	(0.85; 0.95)

Number of clinical triggers 105301; Number of clinical actions 20661. ICC for practice = 0.089. Missing values imputed using MICE.

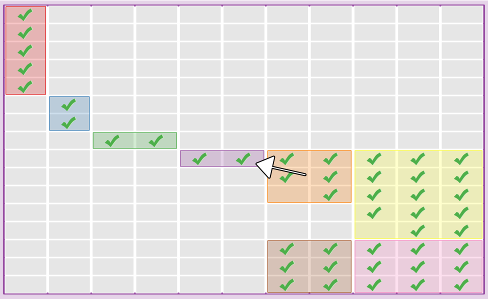

Mixed−effects model for 'high−risk diagnosis' and 'statin'. Incident clinical trigger

	HR	95% CI
Quintile 1	1	(Reference)
Quintile 2	0.97	(0.92; 1.02)
Quintile 3	1.01	(0.96; 1.07)
Quintile 4	1.02	(0.97; 1.08)
Quintile 5	1.01	(0.96; 1.07)
Age 35 to 39	0.55	(0.50; 0.61)
Age 40 to 44	0.79	(0.73; 0.85)
Age 45 to 49	0.92	(0.86; 0.98)
Age 50 to 54	1	(Reference)
Age 55 to 59	1.06	(1.00; 1.13)
Age 60 to 64	1.11	(1.04; 1.18)
Age 65 to 69	1.10	(1.04; 1.18)
Age 70 to 74	1.10	(1.02; 1.17)
Age 75 to 79	0.96	(0.90; 1.03)
Age 80 to 84	0.74	(0.68; 0.80)
Age 85+	0.48	(0.44; 0.52)
Male	1	(Reference)
Female	1.09	(1.06; 1.13)
Non−smoker	1	(Reference)
Smoker	1.12	(1.08; 1.17)
BMI low / norm.	1	(Reference)
Overweight	1.06	(1.01; 1.11)
Obese	0.99	(0.93; 1.04)
Chol:HDL < 4	1	(Reference)
Chol:HDL >= 4	1.49	(1.40; 1.59)
No oth. co.	1	(Reference)
Other co.	0.72	(0.70; 0.75)
Indication 1	1	(Reference)
Indication 2	1.15	(1.10; 1.20)
Indic. years	1.00	(1.00; 1.01)

Number of clinical triggers 34387; Number of clinical actions 19389. ICC for practice = 0.062. Missing values imputed using MICE.

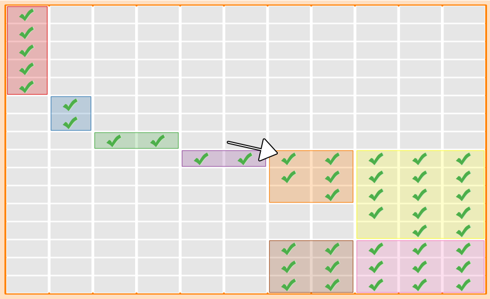

Mixed−effects model for 'stable angina' and 'statin'. Incident clinical trigger

	HR	95% CI
Quintile 1	1	(Reference)
Quintile 2	0.96	(0.87; 1.07)
Quintile 3	1.04	(0.94; 1.15)
Quintile 4	0.99	(0.89; 1.10)
Quintile 5	0.87	(0.79; 0.97)
Age 35 to 39	0.40	(0.27; 0.58)
Age 40 to 44	0.67	(0.54; 0.84)
Age 45 to 49	0.87	(0.74; 1.03)
Age 50 to 54	1	(Reference)
Age 55 to 59	1.08	(0.95; 1.23)
Age 60 to 64	1.08	(0.95; 1.24)
Age 65 to 69	1.00	(0.88; 1.14)
Age 70 to 74	0.85	(0.74; 0.97)
Age 75 to 79	0.66	(0.57; 0.76)
Age 80 to 84	0.50	(0.43; 0.58)
Age 85+	0.24	(0.20; 0.29)
Male	1	(Reference)
Female	0.96	(0.90; 1.03)
Non−smoker	1	(Reference)
Smoker	0.95	(0.88; 1.03)
BMI low / norm.	1	(Reference)
Overweight	1.06	(0.98; 1.15)
Obese	0.88	(0.80; 0.97)
No hyp.	1	(Reference)
Hyp. contr.	0.91	(0.85; 0.98)
Hyp. uncontr.	1.09	(0.99; 1.19)
Untreat. hyp.	1.28	(1.15; 1.44)
Chol:HDL < 4	1	(Reference)
Chol:HDL >= 4	1.44	(1.27; 1.63)
No CVA	1	(Reference)
CVA	1.13	(0.99; 1.30)
No oth. co.	1	(Reference)
Other co.	0.63	(0.59; 0.68)
Indication 1	1	(Reference)
Indication 2	1.21	(1.11; 1.31)
Indication 3	1.10	(0.85; 1.42)
Indic. years	1.00	(0.99; 1.01)

Number of clinical triggers 11104; Number of clinical actions 4660. ICC for practice = 0.04. Missing values imputed using MICE.

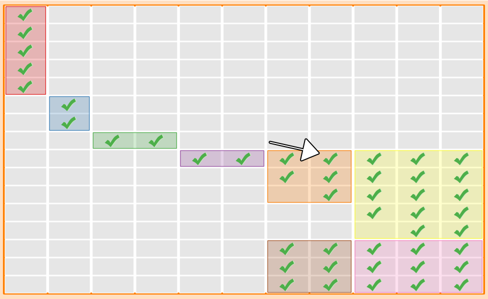

Mixed−effects model for 'stable angina and diabetes' and 'statin'.
Incident clinical trigger

	HR	95% CI
Quintile 1	1	(Reference)
Quintile 2	0.96	(0.75; 1.21)
Quintile 3	1.09	(0.86; 1.36)
Quintile 4	1.07	(0.85; 1.35)
Quintile 5	0.97	(0.77; 1.23)
Age 35 to 39	0.94	(0.52; 1.71)
Age 40 to 44	0.96	(0.56; 1.65)
Age 45 to 49	1.15	(0.76; 1.72)
Age 50 to 54	1	(Reference)
Age 55 to 59	0.88	(0.63; 1.24)
Age 60 to 64	0.88	(0.64; 1.21)
Age 65 to 69	0.83	(0.60; 1.13)
Age 70 to 74	0.76	(0.56; 1.04)
Age 75 to 79	0.64	(0.47; 0.88)
Age 80 to 84	0.50	(0.36; 0.70)
Age 85+	0.24	(0.16; 0.36)
Male	1	(Reference)
Female	1.08	(0.94; 1.24)
Non−smoker	1	(Reference)
Smoker	0.93	(0.77; 1.12)
BMI low / norm.	1	(Reference)
Overweight	1.01	(0.82; 1.24)
Obese	0.96	(0.79; 1.16)
No hyp.	1	(Reference)
Hyp. contr.	1.01	(0.86; 1.19)
Hyp. uncontr.	1.38	(1.13; 1.68)
Untreat. hyp.	1.34	(0.99; 1.82)
Chol:HDL < 4	1	(Reference)
Chol:HDL >= 4	1.52	(1.29; 1.78)
No CVA	1	(Reference)
CVA	0.98	(0.78; 1.23)
No oth. co.	1	(Reference)
Other co.	0.73	(0.63; 0.84)
Indication 1	1	(Reference)
Indication 2	2.34	(1.51; 3.63)
Indication 3	2.41	(1.53; 3.79)
Indication 4	1.15	(0.38; 3.49)

	HR	95% CI
Indic. years	1.00	(0.99; 1.01)

Number of clinical triggers 2457; Number of clinical actions 968. ICC for practice = 0.067. Missing values imputed using MICE.

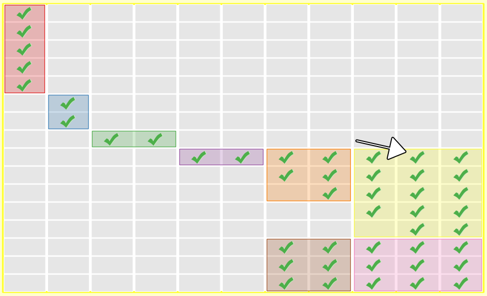

Mixed−effects model for 'unstable angina' and 'statin'. Incident clinical trigger

	HR	95% CI
Quintile 1	1	(Reference)
Quintile 2	1.12	(0.96; 1.32)
Quintile 3	1.18	(1.01; 1.38)
Quintile 4	1.03	(0.88; 1.21)
Quintile 5	1.03	(0.88; 1.20)
Age 35 to 39	0.72	(0.44; 1.18)
Age 40 to 44	0.75	(0.56; 1.01)
Age 45 to 49	1.02	(0.80; 1.30)
Age 50 to 54	1	(Reference)
Age 55 to 59	1.16	(0.94; 1.44)
Age 60 to 64	1.35	(1.09; 1.66)
Age 65 to 69	1.26	(1.01; 1.57)
Age 70 to 74	1.14	(0.92; 1.42)
Age 75 to 79	0.96	(0.77; 1.21)
Age 80 to 84	0.87	(0.70; 1.10)
Age 85+	0.70	(0.55; 0.88)
Male	1	(Reference)
Female	0.81	(0.74; 0.90)
Non−smoker	1	(Reference)
Smoker	1.16	(1.03; 1.30)
BMI low / norm.	1	(Reference)
Overweight	1.20	(1.06; 1.36)
Obese	1.18	(1.03; 1.36)
No hyp.	1	(Reference)
Hyp. contr.	0.91	(0.82; 1.01)
Hyp. uncontr.	1.08	(0.94; 1.25)
Untreat. hyp.	1.13	(0.91; 1.40)
Chol:HDL < 4	1	(Reference)
Chol:HDL >= 4	1.02	(0.90; 1.16)
No CVA	1	(Reference)
CVA	0.83	(0.70; 0.97)
No oth. co.	1	(Reference)
Other co.	0.71	(0.64; 0.78)
No diabetes	1	(Reference)
Diabetes	0.87	(0.76; 1.00)
Elect. adm.	1	(Reference)
Emer. adm.	1.14	(0.88; 1.46)

	HR	95% CI
Other adm.	1.48	(1.13; 1.93)
Cardiac cen.	1	(Reference)
Other cen.	0.76	(0.62; 0.94)
Cardiology	1	(Reference)
Med. spec.	0.53	(0.48; 0.59)
Other spec.	0.41	(0.34; 0.50)
Indication 1	1	(Reference)
Indication 2	1.26	(1.12; 1.43)
Indication 3	1.22	(1.04; 1.43)
Indication 4	0.87	(0.66; 1.15)
Indication 5+	0.57	(0.36; 0.91)
Indic. years	1.00	(0.99; 1.01)

Number of clinical triggers 4462; Number of clinical actions 2178. ICC for practice = 0.079. ICC for hospital = 0.011. Missing values imputed using MICE.

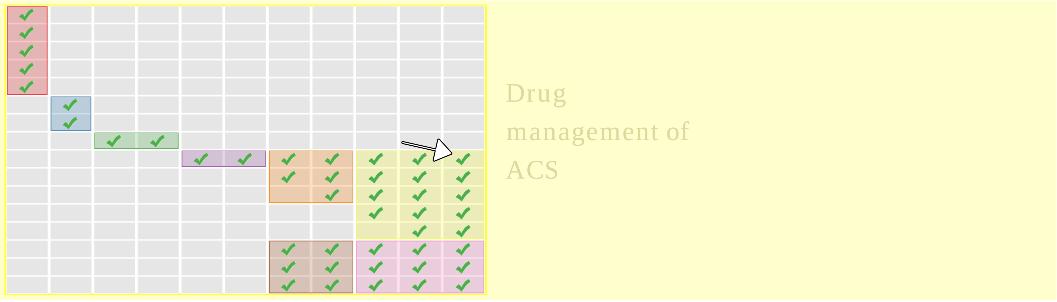

Mixed−effects model for 'MI' and 'statin'. Incident clinical trigger

	HR	95% CI
Quintile 1	1	(Reference)
Quintile 2	1.02	(0.92; 1.13)
Quintile 3	1.00	(0.90; 1.11)
Quintile 4	1.04	(0.94; 1.15)
Quintile 5	0.97	(0.87; 1.08)
Age 35 to 39	1.21	(0.97; 1.51)
Age 40 to 44	1.08	(0.91; 1.28)
Age 45 to 49	1.26	(1.09; 1.46)
Age 50 to 54	1	(Reference)
Age 55 to 59	1.03	(0.91; 1.17)
Age 60 to 64	1.02	(0.90; 1.15)
Age 65 to 69	0.95	(0.84; 1.08)
Age 70 to 74	0.86	(0.76; 0.98)
Age 75 to 79	0.78	(0.68; 0.89)
Age 80 to 84	0.66	(0.58; 0.76)
Age 85+	0.38	(0.33; 0.44)
Male	1	(Reference)
Female	0.93	(0.87; 0.99)
Non−smoker	1	(Reference)
Smoker	1.04	(0.97; 1.11)
BMI low / norm.	1	(Reference)
Overweight	1.15	(1.06; 1.24)
Obese	1.05	(0.96; 1.14)
No hyp.	1	(Reference)
Hyp. contr.	0.97	(0.90; 1.03)
Hyp. uncontr.	1.05	(0.96; 1.16)
Untreat. hyp.	1.03	(0.91; 1.16)
Chol:HDL < 4	1	(Reference)
Chol:HDL >= 4	1.18	(1.09; 1.27)
No CVA	1	(Reference)
CVA	0.77	(0.68; 0.87)
No oth. co.	1	(Reference)
Other co.	0.68	(0.64; 0.72)
No diabetes	1	(Reference)
Diabetes	0.86	(0.78; 0.94)
Elect. adm.	1	(Reference)
Emer. adm.	1.28	(1.05; 1.56)

	HR	95% CI
Other adm.	1.44	(1.17; 1.77)
Cardiac cen.	1	(Reference)
Other cen.	0.84	(0.62; 1.14)
Cardiology	1	(Reference)
Med. spec.	0.64	(0.60; 0.69)
Other spec.	0.40	(0.35; 0.46)
Indication 1	1	(Reference)
Indication 2	1.16	(1.08; 1.26)
Indication 3	1.13	(1.02; 1.26)
Indication 4	1.09	(0.90; 1.32)
Indication 5+	0.93	(0.66; 1.32)
Indic. years	1.00	(0.99; 1.00)

Number of clinical triggers 10442; Number of clinical actions 5372. ICC for practice = 0.095. ICC for hospital = 0.041. Missing values imputed using MICE.

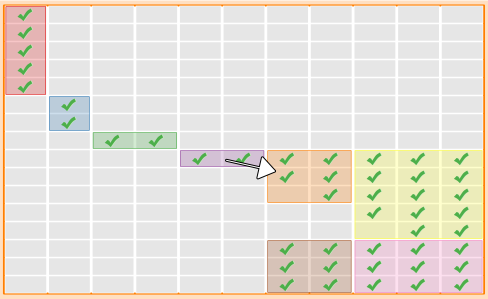

Drug management of chronic CHD

Mixed−effects model for 'stable angina' and 'aspirin'. Incident clinical trigger

	HR	95% CI
Quintile 1	1	(Reference)
Quintile 2	0.95	(0.85; 1.06)
Quintile 3	1.02	(0.91; 1.14)
Quintile 4	1.00	(0.89; 1.12)
Quintile 5	0.98	(0.88; 1.10)
Age 35 to 39	0.49	(0.34; 0.72)
Age 40 to 44	0.95	(0.76; 1.18)
Age 45 to 49	0.97	(0.81; 1.15)
Age 50 to 54	1	(Reference)
Age 55 to 59	1.18	(1.03; 1.35)
Age 60 to 64	1.19	(1.04; 1.36)
Age 65 to 69	1.06	(0.92; 1.22)
Age 70 to 74	0.96	(0.83; 1.12)
Age 75 to 79	0.90	(0.77; 1.04)
Age 80 to 84	0.89	(0.76; 1.05)
Age 85+	0.71	(0.59; 0.85)
Male	1	(Reference)
Female	0.86	(0.80; 0.92)
Non−smoker	1	(Reference)
Smoker	1.06	(0.97; 1.16)
BMI low / norm.	1	(Reference)
Overweight	1.00	(0.93; 1.09)
Obese	0.90	(0.82; 0.98)
No hyp.	1	(Reference)
Hyp. contr.	0.91	(0.84; 0.98)
Hyp. uncontr.	1.03	(0.93; 1.13)
Untreat. hyp.	1.26	(1.11; 1.44)
Chol:HDL < 4	1	(Reference)
Chol:HDL >= 4	1.13	(0.99; 1.28)
No CVA	1	(Reference)
CVA	0.81	(0.70; 0.94)
No oth. co.	1	(Reference)
Other co.	0.64	(0.59; 0.69)

Number of clinical triggers 9433; Number of clinical actions 3923. ICC for practice = 0.056. Missing values imputed using MICE.

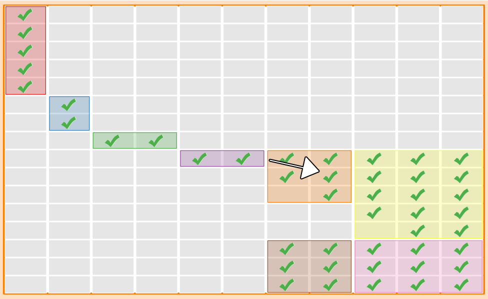

Mixed−effects model for 'stable angina and diabetes' and 'aspirin'.
Incident clinical trigger

	HR	95% CI
Quintile 1	1	(Reference)
Quintile 2	0.88	(0.70; 1.12)
Quintile 3	1.09	(0.87; 1.37)
Quintile 4	0.96	(0.77; 1.20)
Quintile 5	1.01	(0.81; 1.27)
Age 35 to 39	1.12	(0.60; 2.10)
Age 40 to 44	1.14	(0.72; 1.81)
Age 45 to 49	1.07	(0.71; 1.59)
Age 50 to 54	1	(Reference)
Age 55 to 59	1.10	(0.80; 1.50)
Age 60 to 64	1.00	(0.74; 1.36)
Age 65 to 69	0.88	(0.65; 1.20)
Age 70 to 74	0.94	(0.69; 1.27)
Age 75 to 79	0.93	(0.68; 1.28)
Age 80 to 84	0.99	(0.70; 1.39)
Age 85+	0.81	(0.55; 1.20)
Male	1	(Reference)
Female	0.87	(0.75; 0.99)
Non−smoker	1	(Reference)
Smoker	1.00	(0.83; 1.21)
BMI low / norm.	1	(Reference)
Overweight	1.11	(0.90; 1.38)
Obese	0.99	(0.80; 1.22)
No hyp.	1	(Reference)
Hyp. contr.	0.93	(0.79; 1.10)
Hyp. uncontr.	1.18	(0.97; 1.44)
Untreat. hyp.	1.22	(0.87; 1.73)
Chol:HDL < 4	1	(Reference)
Chol:HDL >= 4	1.21	(1.00; 1.47)
No CVA	1	(Reference)
CVA	0.90	(0.71; 1.16)
No oth. co.	1	(Reference)
Other co.	0.69	(0.60; 0.80)
Indication 1	1	(Reference)
Indication 2	0.44	(0.36; 0.54)
Indic. years	1.01	(0.99; 1.03)

Number of clinical triggers 2736; Number of clinical actions 919. ICC for practice = 0.066. Missing values imputed using MICE.

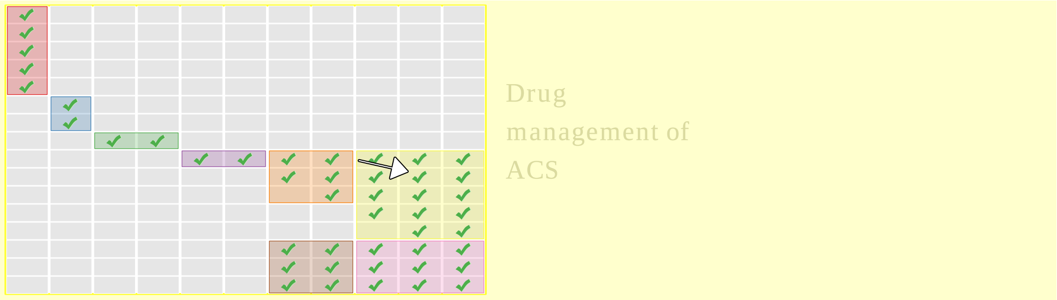

Mixed−effects model for 'unstable angina' and 'aspirin'. Incident clinical trigger

	HR	95% CI
Quintile 1	1	(Reference)
Quintile 2	1.08	(0.91; 1.28)
Quintile 3	1.26	(1.06; 1.48)
Quintile 4	1.15	(0.97; 1.35)
Quintile 5	1.24	(1.05; 1.46)
Age 35 to 39	0.91	(0.57; 1.46)
Age 40 to 44	0.68	(0.51; 0.90)
Age 45 to 49	0.90	(0.71; 1.15)
Age 50 to 54	1	(Reference)
Age 55 to 59	1.03	(0.85; 1.25)
Age 60 to 64	1.18	(0.97; 1.43)
Age 65 to 69	1.03	(0.83; 1.26)
Age 70 to 74	1.01	(0.81; 1.25)
Age 75 to 79	0.81	(0.65; 1.02)
Age 80 to 84	1.03	(0.82; 1.28)
Age 85+	0.90	(0.71; 1.13)
Male	1	(Reference)
Female	0.78	(0.71; 0.86)
Non−smoker	1	(Reference)
Smoker	1.20	(1.07; 1.36)
BMI low / norm.	1	(Reference)
Overweight	0.95	(0.83; 1.09)
Obese	1.05	(0.92; 1.20)
No hyp.	1	(Reference)
Hyp. contr.	1.00	(0.90; 1.12)
Hyp. uncontr.	1.26	(1.09; 1.44)
Untreat. hyp.	1.32	(1.05; 1.64)
Chol:HDL < 4	1	(Reference)
Chol:HDL >= 4	1.05	(0.85; 1.31)
No CVA	1	(Reference)
CVA	0.78	(0.64; 0.94)
No oth. co.	1	(Reference)
Other co.	0.75	(0.68; 0.83)
No diabetes	1	(Reference)
Diabetes	0.93	(0.82; 1.06)
Elect. adm.	1	(Reference)
Emer. adm.	0.92	(0.72; 1.19)

	HR	95% CI
Other adm.	1.68	(1.29; 2.19)
Cardiac cen.	1	(Reference)
Other cen.	0.77	(0.59; 0.99)
Cardiology	1	(Reference)
Med. spec.	0.66	(0.59; 0.74)
Other spec.	0.58	(0.48; 0.71)
Indication 1	1	(Reference)
Indication 2	0.80	(0.71; 0.91)
Indication 3	0.64	(0.51; 0.79)
Indication 4	0.51	(0.35; 0.74)
Indication 5+	0.43	(0.27; 0.68)
Indic. years	0.99	(0.98; 1.00)

Number of clinical triggers 4172; Number of clinical actions 2041. ICC for practice = 0.084. ICC for hospital = 0.021. Missing values imputed using MICE.

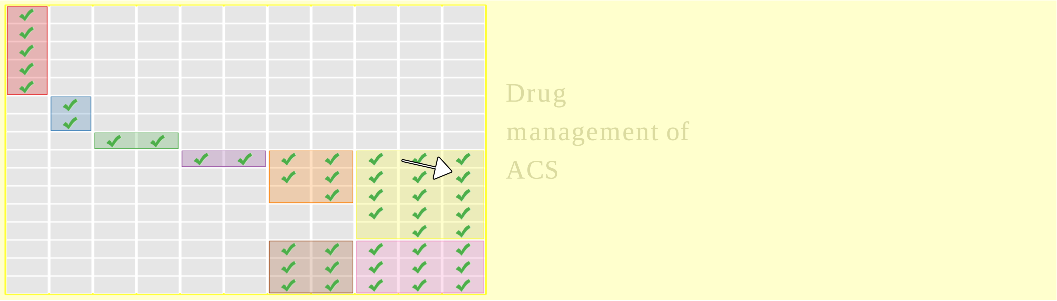

Mixed−effects model for 'MI' and 'aspirin'. Incident clinical trigger

	HR	95% CI
Quintile 1	1	(Reference)
Quintile 2	0.98	(0.88; 1.09)
Quintile 3	1.01	(0.91; 1.12)
Quintile 4	0.98	(0.88; 1.09)
Quintile 5	0.99	(0.89; 1.10)
Age 35 to 39	1.12	(0.90; 1.39)
Age 40 to 44	1.10	(0.93; 1.29)
Age 45 to 49	1.10	(0.95; 1.26)
Age 50 to 54	1	(Reference)
Age 55 to 59	1.00	(0.89; 1.13)
Age 60 to 64	0.98	(0.87; 1.11)
Age 65 to 69	0.98	(0.87; 1.11)
Age 70 to 74	0.83	(0.73; 0.94)
Age 75 to 79	0.78	(0.68; 0.90)
Age 80 to 84	0.68	(0.58; 0.79)
Age 85+	0.61	(0.52; 0.71)
Male	1	(Reference)
Female	0.85	(0.79; 0.90)
Non−smoker	1	(Reference)
Smoker	1.06	(0.98; 1.15)
BMI low / norm.	1	(Reference)
Overweight	1.08	(1.00; 1.16)
Obese	1.03	(0.95; 1.13)
No hyp.	1	(Reference)
Hyp. contr.	0.98	(0.92; 1.05)
Hyp. uncontr.	1.02	(0.93; 1.11)
Untreat. hyp.	1.06	(0.94; 1.20)
Chol:HDL < 4	1	(Reference)
Chol:HDL >= 4	1.15	(1.00; 1.31)
No CVA	1	(Reference)
CVA	0.56	(0.48; 0.66)
No oth. co.	1	(Reference)
Other co.	0.72	(0.67; 0.77)
No diabetes	1	(Reference)
Diabetes	0.88	(0.80; 0.96)
Elect. adm.	1	(Reference)
Emer. adm.	1.13	(0.92; 1.39)

	HR	95% CI
Other adm.	1.35	(1.08; 1.67)
Cardiac cen.	1	(Reference)
Other cen.	0.85	(0.65; 1.12)
Cardiology	1	(Reference)
Med. spec.	0.72	(0.66; 0.77)
Other spec.	0.46	(0.40; 0.52)
Indication 1	1	(Reference)
Indication 2	0.97	(0.89; 1.06)
Indication 3	0.86	(0.73; 1.01)
Indication 4	0.67	(0.48; 0.95)
Indication 5+	0.49	(0.26; 0.92)
Indic. years	0.98	(0.97; 0.99)

Number of clinical triggers 9577; Number of clinical actions 5098. ICC for practice = 0.107. ICC for hospital = 0.031. Missing values imputed using MICE.

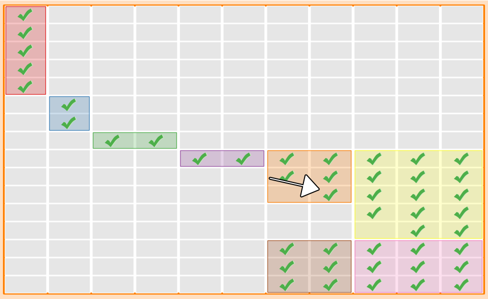

Drug management of chronic CHD

Mixed−effects model for 'stable angina and diabetes' and 'ACE inhibitor'. Incident clinical trigger

	HR	95% CI
Quintile 1	1	(Reference)
Quintile 2	1.12	(0.89; 1.40)
Quintile 3	1.20	(0.97; 1.48)
Quintile 4	1.06	(0.86; 1.31)
Quintile 5	1.12	(0.91; 1.39)
Age 35 to 39	0.87	(0.41; 1.82)
Age 40 to 44	0.70	(0.42; 1.19)
Age 45 to 49	0.74	(0.49; 1.13)
Age 50 to 54	1	(Reference)
Age 55 to 59	0.81	(0.60; 1.10)
Age 60 to 64	0.82	(0.61; 1.09)
Age 65 to 69	0.88	(0.66; 1.16)
Age 70 to 74	0.92	(0.70; 1.23)
Age 75 to 79	0.73	(0.54; 0.99)
Age 80 to 84	0.88	(0.64; 1.21)
Age 85+	0.60	(0.42; 0.88)
Male	1	(Reference)
Female	0.91	(0.80; 1.03)
Non−smoker	1	(Reference)
Smoker	0.92	(0.77; 1.09)
BMI low / norm.	1	(Reference)
Overweight	1.10	(0.90; 1.34)
Obese	1.27	(1.05; 1.54)
No hyp.	1	(Reference)
Hyp. contr.	1.36	(1.18; 1.57)
Hyp. uncontr.	2.72	(2.27; 3.26)
Untreat. hyp.	1.61	(1.26; 2.05)
Chol:HDL < 4	1	(Reference)
Chol:HDL >= 4	1.21	(1.05; 1.39)
No CVA	1	(Reference)
CVA	1.16	(0.96; 1.40)
No oth. co.	1	(Reference)
Other co.	0.88	(0.77; 1.01)

Number of clinical triggers 3361; Number of clinical actions 1092. ICC for practice = 0.03. Missing values imputed using MICE.

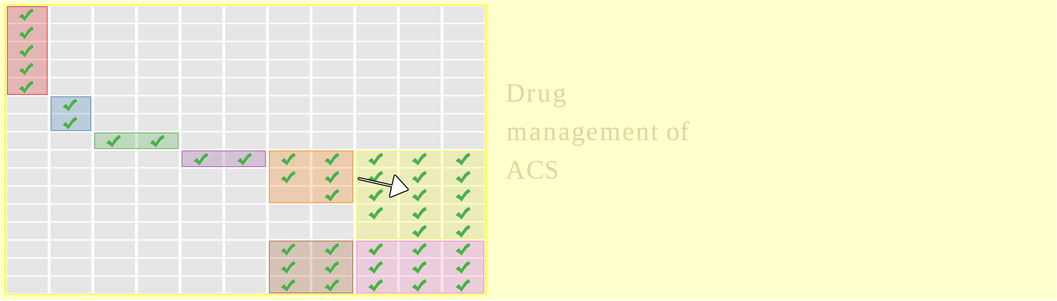

Mixed−effects model for 'unstable angina' and 'ACE inhibitor'.
Incident clinical trigger

	HR	95% CI
Quintile 1	1	(Reference)
Quintile 2	1.06	(0.90; 1.25)
Quintile 3	1.21	(1.04; 1.42)
Quintile 4	1.19	(1.01; 1.39)
Quintile 5	1.06	(0.91; 1.25)
Age 35 to 39	0.71	(0.41; 1.23)
Age 40 to 44	0.70	(0.51; 0.97)
Age 45 to 49	1.03	(0.79; 1.33)
Age 50 to 54	1	(Reference)
Age 55 to 59	1.04	(0.84; 1.29)
Age 60 to 64	1.26	(1.02; 1.56)
Age 65 to 69	1.24	(1.00; 1.53)
Age 70 to 74	1.51	(1.21; 1.89)
Age 75 to 79	1.23	(0.98; 1.55)
Age 80 to 84	1.27	(1.00; 1.60)
Age 85+	1.03	(0.80; 1.31)
Male	1	(Reference)
Female	0.80	(0.72; 0.88)
Non−smoker	1	(Reference)
Smoker	1.35	(1.21; 1.51)
BMI low / norm.	1	(Reference)
Overweight	1.05	(0.93; 1.18)
Obese	1.07	(0.93; 1.24)
No hyp.	1	(Reference)
Hyp. contr.	1.20	(1.08; 1.33)
Hyp. uncontr.	1.52	(1.30; 1.78)
Untreat. hyp.	1.26	(1.02; 1.55)
Chol:HDL < 4	1	(Reference)
Chol:HDL >= 4	1.03	(0.84; 1.27)
No CVA	1	(Reference)
CVA	0.98	(0.84; 1.14)
No oth. co.	1	(Reference)
Other co.	0.76	(0.69; 0.84)
No diabetes	1	(Reference)
Diabetes	1.17	(1.02; 1.34)
Elect. adm.	1	(Reference)
Emer. adm.	1.51	(1.20; 1.88)

	HR	95% CI
Other adm.	2.27	(1.79; 2.86)
Cardiac cen.	1	(Reference)
Other cen.	0.75	(0.57; 0.97)
Cardiology	1	(Reference)
Med. spec.	0.55	(0.49; 0.62)
Other spec.	0.52	(0.43; 0.62)
Indication 1	1	(Reference)
Indication 2	1.15	(1.01; 1.30)
Indication 3	0.96	(0.75; 1.22)
Indication 4	1.03	(0.70; 1.51)
Indication 5+	0.57	(0.35; 0.92)
Indic. years	0.96	(0.93; 1.00)

Number of clinical triggers 5287; Number of clinical actions 1967. ICC for practice = 0.039. ICC for hospital = 0.024. Missing values imputed using MICE.

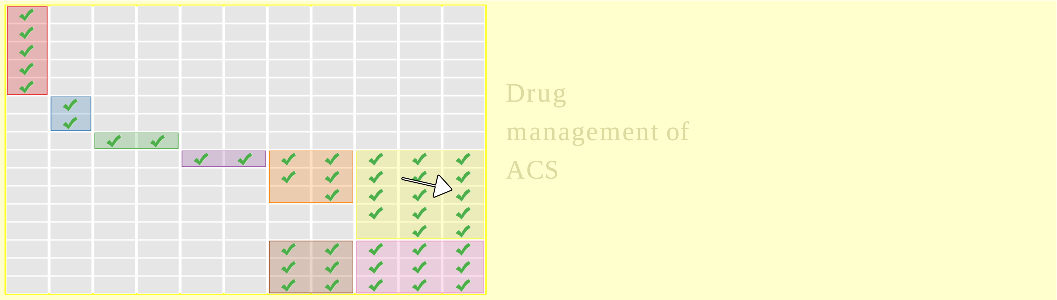

Mixed−effects model for 'MI' and 'ACE inhibitor'. Incident clinical trigger

	HR	95% CI
Quintile 1	1	(Reference)
Quintile 2	1.05	(0.95; 1.16)
Quintile 3	1.01	(0.91; 1.12)
Quintile 4	1.02	(0.92; 1.12)
Quintile 5	1.02	(0.91; 1.13)
Age 35 to 39	0.86	(0.69; 1.07)
Age 40 to 44	1.01	(0.86; 1.19)
Age 45 to 49	1.09	(0.94; 1.25)
Age 50 to 54	1	(Reference)
Age 55 to 59	0.96	(0.85; 1.08)
Age 60 to 64	1.04	(0.92; 1.17)
Age 65 to 69	0.98	(0.86; 1.11)
Age 70 to 74	0.83	(0.73; 0.94)
Age 75 to 79	0.84	(0.74; 0.96)
Age 80 to 84	0.78	(0.69; 0.90)
Age 85+	0.46	(0.40; 0.54)
Male	1	(Reference)
Female	0.89	(0.83; 0.94)
Non−smoker	1	(Reference)
Smoker	1.05	(0.98; 1.12)
BMI low / norm.	1	(Reference)
Overweight	1.22	(1.13; 1.31)
Obese	1.20	(1.10; 1.31)
No hyp.	1	(Reference)
Hyp. contr.	1.02	(0.95; 1.09)
Hyp. uncontr.	1.19	(1.08; 1.32)
Untreat. hyp.	1.12	(1.00; 1.25)
Chol:HDL < 4	1	(Reference)
Chol:HDL >= 4	1.10	(1.01; 1.20)
No CVA	1	(Reference)
CVA	0.82	(0.73; 0.91)
No oth. co.	1	(Reference)
Other co.	0.73	(0.68; 0.77)
No diabetes	1	(Reference)
Diabetes	1.05	(0.96; 1.15)
Elect. adm.	1	(Reference)
Emer. adm.	1.26	(1.04; 1.53)

	HR	95% CI
Other adm.	1.45	(1.19; 1.77)
Cardiac cen.	1	(Reference)
Other cen.	1.04	(0.82; 1.32)
Cardiology	1	(Reference)
Med. spec.	0.69	(0.64; 0.74)
Other spec.	0.38	(0.34; 0.44)
Indication 1	1	(Reference)
Indication 2	1.04	(0.95; 1.14)
Indication 3	0.99	(0.84; 1.18)
Indication 4	0.91	(0.61; 1.36)
Indication 5+	0.83	(0.41; 1.70)
Indic. years	0.94	(0.91; 0.96)

Number of clinical triggers 10595; Number of clinical actions 5270. ICC for practice = 0.048. ICC for hospital = 0.024. Missing values imputed using MICE.

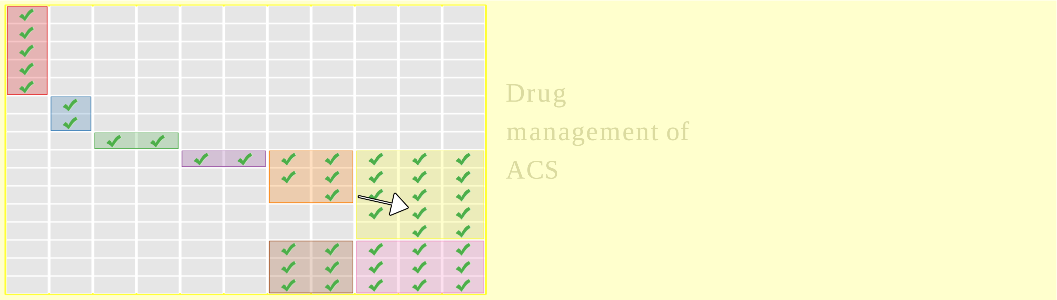

Mixed−effects model for 'unstable angina' and 'beta−blocker'.
Incident clinical trigger

	HR	95% CI
Quintile 1	1	(Reference)
Quintile 2	0.60	(0.39; 0.92)
Quintile 3	0.83	(0.57; 1.21)
Quintile 4	0.86	(0.59; 1.26)
Quintile 5	0.91	(0.63; 1.32)
Age 35 to 39	0.85	(0.20; 3.68)
Age 40 to 44	1.80	(0.94; 3.46)
Age 45 to 49	0.73	(0.35; 1.56)
Age 50 to 54	1	(Reference)
Age 55 to 59	1.11	(0.66; 1.86)
Age 60 to 64	0.80	(0.46; 1.36)
Age 65 to 69	0.58	(0.33; 1.02)
Age 70 to 74	0.79	(0.46; 1.36)
Age 75 to 79	0.64	(0.36; 1.12)
Age 80 to 84	0.87	(0.50; 1.51)
Age 85+	0.34	(0.17; 0.66)
Male	1	(Reference)
Female	1.03	(0.80; 1.32)
Non−smoker	1	(Reference)
Smoker	0.79	(0.57; 1.10)
BMI low / norm.	1	(Reference)
Overweight	0.94	(0.67; 1.33)
Obese	0.78	(0.53; 1.15)
No hyp.	1	(Reference)
Hyp. contr.	1.64	(1.21; 2.22)
Hyp. uncontr.	2.38	(1.66; 3.41)
Untreat. hyp.	1.72	(0.94; 3.16)
Chol:HDL < 4	1	(Reference)
Chol:HDL >= 4	1.12	(0.73; 1.70)
No CVA	1	(Reference)
CVA	0.98	(0.67; 1.44)
No oth. co.	1	(Reference)
Other co.	0.40	(0.30; 0.53)
No diabetes	1	(Reference)
Diabetes	0.75	(0.55; 1.04)
Elect. adm.	1	(Reference)
Emer. adm.	1.53	(0.87; 2.70)

	HR	95% CI
Other adm.	1.66	(0.91; 3.05)
Cardiac cen.	1	(Reference)
Other cen.	0.86	(0.30; 2.47)
Cardiology	1	(Reference)
Med. spec.	1.21	(0.88; 1.67)
Other spec.	1.33	(0.84; 2.11)
Indication 1	1	(Reference)
Indication 2	0.75	(0.53; 1.07)
Indication 3	0.68	(0.36; 1.26)
Indication 4	0.24	(0.06; 1.03)
Indication 5+	0.27	(0.06; 1.19)
Indic. years	0.92	(0.80; 1.08)

Number of clinical triggers 10405; Number of clinical actions 285. ICC for practice = 0.093. ICC for hospital = 0.292. Missing values imputed using MICE.

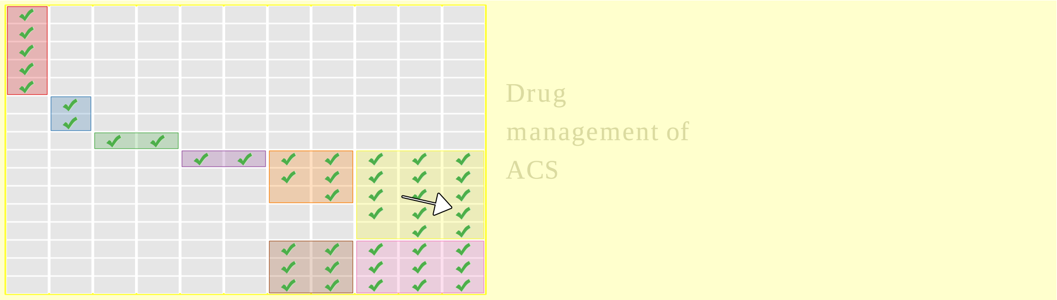

Mixed−effects model for 'MI' and 'beta−blocker'. Incident clinical trigger

	HR	95% CI
Quintile 1	1	(Reference)
Quintile 2	0.93	(0.66; 1.30)
Quintile 3	0.76	(0.54; 1.07)
Quintile 4	0.81	(0.58; 1.13)
Quintile 5	0.83	(0.59; 1.17)
Age 35 to 39	1.06	(0.49; 2.30)
Age 40 to 44	1.70	(1.00; 2.88)
Age 45 to 49	1.18	(0.71; 1.96)
Age 50 to 54	1	(Reference)
Age 55 to 59	0.83	(0.53; 1.31)
Age 60 to 64	0.95	(0.62; 1.47)
Age 65 to 69	0.87	(0.55; 1.35)
Age 70 to 74	0.60	(0.37; 0.97)
Age 75 to 79	0.56	(0.34; 0.92)
Age 80 to 84	0.58	(0.35; 0.96)
Age 85+	0.24	(0.13; 0.44)
Male	1	(Reference)
Female	1.07	(0.84; 1.35)
Non−smoker	1	(Reference)
Smoker	0.77	(0.59; 1.02)
BMI low / norm.	1	(Reference)
Overweight	1.10	(0.85; 1.44)
Obese	0.98	(0.70; 1.36)
No hyp.	1	(Reference)
Hyp. contr.	1.40	(1.09; 1.80)
Hyp. uncontr.	1.56	(1.13; 2.14)
Untreat. hyp.	0.83	(0.48; 1.45)
Chol:HDL < 4	1	(Reference)
Chol:HDL >= 4	1.55	(1.01; 2.37)
No CVA	1	(Reference)
CVA	0.75	(0.49; 1.15)
No oth. co.	1	(Reference)
Other co.	0.39	(0.30; 0.52)
No diabetes	1	(Reference)
Diabetes	1.00	(0.76; 1.32)
Elect. adm.	1	(Reference)
Emer. adm.	0.54	(0.29; 0.98)

	HR	95% CI
Other adm.	0.48	(0.25; 0.92)
Cardiac cen.	1	(Reference)
Other cen.	0.69	(0.26; 1.84)
Cardiology	1	(Reference)
Med. spec.	1.32	(1.00; 1.75)
Other spec.	0.53	(0.29; 0.95)
Indication 1	1	(Reference)
Indication 2	0.85	(0.60; 1.19)
Indication 3	0.59	(0.29; 1.20)
Indication 4	0.21	(0.03; 1.55)
Indication 5+	0.00	(0.00; >99)
Indic. years	1.00	(0.85; 1.17)

Number of clinical triggers 16639; Number of clinical actions 363. ICC for practice = 0.021. ICC for hospital = 0.305. Missing values imputed using MICE.

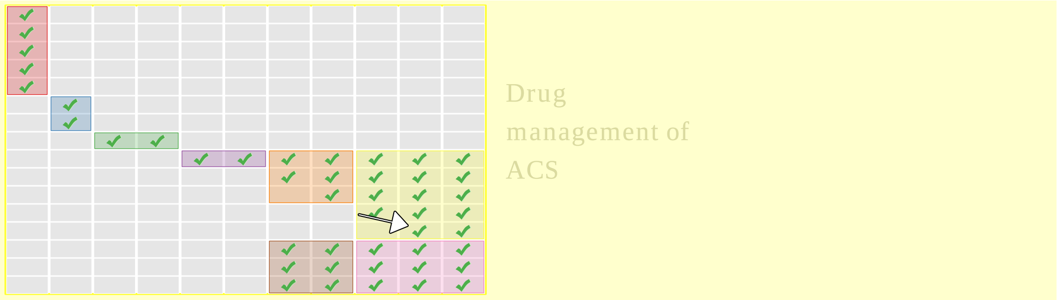

Mixed−effects model for 'unstable angina' and 'clopidogrel'. Incident clinical trigger

	HR	95% CI
Quintile 1	1	(Reference)
Quintile 2	0.99	(0.90; 1.10)
Quintile 3	1.08	(0.98; 1.18)
Quintile 4	1.01	(0.92; 1.11)
Quintile 5	1.02	(0.93; 1.12)
Age 35 to 39	0.88	(0.62; 1.24)
Age 40 to 44	0.83	(0.67; 1.03)
Age 45 to 49	0.98	(0.83; 1.16)
Age 50 to 54	1	(Reference)
Age 55 to 59	0.93	(0.81; 1.06)
Age 60 to 64	0.97	(0.86; 1.10)
Age 65 to 69	0.98	(0.86; 1.11)
Age 70 to 74	0.96	(0.84; 1.09)
Age 75 to 79	0.83	(0.72; 0.94)
Age 80 to 84	0.90	(0.79; 1.03)
Age 85+	0.80	(0.70; 0.92)
Male	1	(Reference)
Female	0.84	(0.79; 0.89)
Non−smoker	1	(Reference)
Smoker	1.15	(1.07; 1.24)
BMI low / norm.	1	(Reference)
Overweight	1.05	(0.97; 1.13)
Obese	1.04	(0.96; 1.12)
No hyp.	1	(Reference)
Hyp. contr.	1.04	(0.97; 1.11)
Hyp. uncontr.	1.02	(0.93; 1.12)
Untreat. hyp.	1.17	(0.99; 1.40)
Chol:HDL < 4	1	(Reference)
Chol:HDL >= 4	1.18	(1.08; 1.29)
No CVA	1	(Reference)
CVA	1.06	(0.98; 1.13)
No oth. co.	1	(Reference)
Other co.	0.96	(0.91; 1.01)
No diabetes	1	(Reference)
Diabetes	1.07	(1.01; 1.14)
Elect. adm.	1	(Reference)
Emer. adm.	1.03	(0.91; 1.16)

	HR	95% CI
Other adm.	1.36	(1.19; 1.55)
Cardiac cen.	1	(Reference)
Other cen.	0.69	(0.53; 0.91)
Cardiology	1	(Reference)
Med. spec.	0.64	(0.60; 0.68)
Other spec.	0.33	(0.29; 0.38)

Number of clinical triggers 13907; Number of clinical actions 5783. ICC for practice = 0.04. ICC for hospital = 0.035. Missing values imputed using MICE.

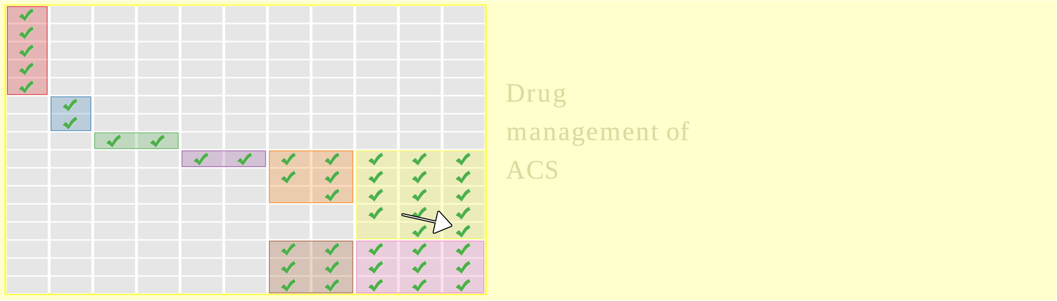

Mixed−effects model for 'MI' and 'clopidogrel'. Incident clinical trigger

	HR	95% CI
Quintile 1	1	(Reference)
Quintile 2	1.04	(0.96; 1.12)
Quintile 3	1.05	(0.97; 1.13)
Quintile 4	1.06	(0.99; 1.15)
Quintile 5	1.03	(0.95; 1.11)
Age 35 to 39	0.93	(0.77; 1.12)
Age 40 to 44	1.00	(0.87; 1.15)
Age 45 to 49	1.03	(0.92; 1.16)
Age 50 to 54	1	(Reference)
Age 55 to 59	0.90	(0.82; 0.99)
Age 60 to 64	0.86	(0.79; 0.95)
Age 65 to 69	0.81	(0.74; 0.89)
Age 70 to 74	0.69	(0.63; 0.76)
Age 75 to 79	0.63	(0.57; 0.69)
Age 80 to 84	0.59	(0.54; 0.65)
Age 85+	0.49	(0.44; 0.54)
Male	1	(Reference)
Female	0.99	(0.94; 1.03)
Non−smoker	1	(Reference)
Smoker	1.03	(0.98; 1.09)
BMI low / norm.	1	(Reference)
Overweight	1.14	(1.08; 1.20)
Obese	1.08	(1.02; 1.15)
No hyp.	1	(Reference)
Hyp. contr.	1.01	(0.96; 1.06)
Hyp. uncontr.	1.06	(1.00; 1.13)
Untreat. hyp.	1.02	(0.92; 1.13)
Chol:HDL < 4	1	(Reference)
Chol:HDL >= 4	1.03	(0.96; 1.11)
No CVA	1	(Reference)
CVA	0.91	(0.86; 0.97)
No oth. co.	1	(Reference)
Other co.	0.85	(0.81; 0.88)
No diabetes	1	(Reference)
Diabetes	0.97	(0.92; 1.02)
Elect. adm.	1	(Reference)
Emer. adm.	1.13	(0.99; 1.30)

	HR	95% CI
Other adm.	1.24	(1.08; 1.43)
Cardiac cen.	1	(Reference)
Other cen.	0.88	(0.73; 1.06)
Cardiology	1	(Reference)
Med. spec.	0.67	(0.64; 0.70)
Other spec.	0.27	(0.24; 0.31)

Number of clinical triggers 20467; Number of clinical actions 10132. ICC for practice = 0.05. ICC for hospital = 0.015. Missing values imputed using MICE.

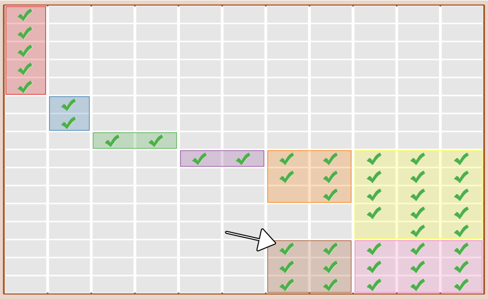

Revascularisation of chronic CHD

Mixed−effects model for 'stable angina' and 'PCI'. Incident clinical trigger

	HR	95% CI
Quintile 1	1	(Reference)
Quintile 2	0.97	(0.80; 1.17)
Quintile 3	0.91	(0.75; 1.09)
Quintile 4	0.78	(0.64; 0.96)
Quintile 5	0.72	(0.58; 0.88)
Age 35 to 39	1.07	(0.61; 1.87)
Age 40 to 44	1.27	(0.89; 1.79)
Age 45 to 49	1.22	(0.92; 1.61)
Age 50 to 54	1	(Reference)
Age 55 to 59	0.99	(0.79; 1.26)
Age 60 to 64	1.03	(0.82; 1.29)
Age 65 to 69	0.84	(0.67; 1.07)
Age 70 to 74	0.75	(0.59; 0.96)
Age 75 to 79	0.35	(0.26; 0.47)
Age 80 to 84	0.24	(0.16; 0.35)
Age 85+	0.07	(0.04; 0.15)
Male	1	(Reference)
Female	0.60	(0.53; 0.68)
Non−smoker	1	(Reference)
Smoker	0.97	(0.84; 1.13)
BMI low / norm.	1	(Reference)
Overweight	1.00	(0.85; 1.18)
Obese	0.82	(0.69; 0.96)
No hyp.	1	(Reference)
Hyp. contr.	1.02	(0.88; 1.17)
Hyp. uncontr.	1.26	(1.07; 1.49)
Untreat. hyp.	1.30	(1.04; 1.63)
Chol:HDL < 4	1	(Reference)
Chol:HDL >= 4	1.75	(1.50; 2.04)
No CVA	1	(Reference)
CVA	0.53	(0.40; 0.70)
No oth. co.	1	(Reference)
Other co.	0.61	(0.52; 0.71)
No prev. acti.	1	(Reference)
1+ prev. acti.	0.00	(0.00; >99)

Number of clinical triggers 18934; Number of clinical actions 1172. ICC for practice = 0.084. Missing values imputed using MICE.

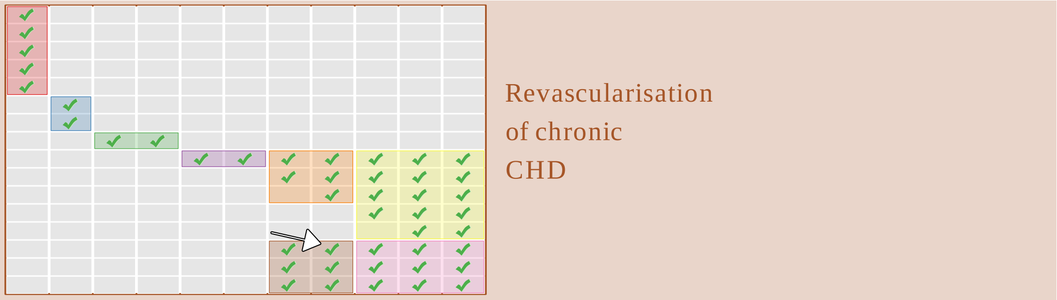

Mixed−effects model for 'stable angina and diabetes' and 'PCI'.
Incident clinical trigger

	HR	95% CI
Quintile 1	1	(Reference)
Quintile 2	0.71	(0.47; 1.06)
Quintile 3	0.82	(0.57; 1.19)
Quintile 4	0.69	(0.47; 1.01)
Quintile 5	0.84	(0.59; 1.21)
Age 35 to 39	1.12	(0.39; 3.19)
Age 40 to 44	1.35	(0.66; 2.77)
Age 45 to 49	1.29	(0.73; 2.28)
Age 50 to 54	1	(Reference)
Age 55 to 59	0.90	(0.58; 1.42)
Age 60 to 64	0.73	(0.46; 1.14)
Age 65 to 69	0.65	(0.42; 1.03)
Age 70 to 74	0.65	(0.41; 1.02)
Age 75 to 79	0.28	(0.16; 0.50)
Age 80 to 84	0.09	(0.03; 0.25)
Age 85+	0.14	(0.05; 0.40)
Male	1	(Reference)
Female	0.69	(0.53; 0.88)
Non−smoker	1	(Reference)
Smoker	0.87	(0.63; 1.21)
BMI low / norm.	1	(Reference)
Overweight	1.54	(0.99; 2.40)
Obese	1.47	(0.95; 2.27)
No hyp.	1	(Reference)
Hyp. contr.	1.13	(0.83; 1.52)
Hyp. uncontr.	1.14	(0.80; 1.64)
Untreat. hyp.	1.46	(0.80; 2.66)
Chol:HDL < 4	1	(Reference)
Chol:HDL >= 4	0.97	(0.72; 1.31)
No CVA	1	(Reference)
CVA	0.91	(0.62; 1.33)
No oth. co.	1	(Reference)
Other co.	0.67	(0.51; 0.88)
Indication 1	1	(Reference)
Indication 2	0.40	(0.27; 0.60)
Indic. years	0.95	(0.90; 1.00)
No prev. acti.	1	(Reference)

	HR	95% CI
1+ prev. acti.	1.46	(0.58; 3.68)

Number of clinical triggers 8956; Number of clinical actions 300. ICC for practice = 0.09. Missing values imputed using MICE.

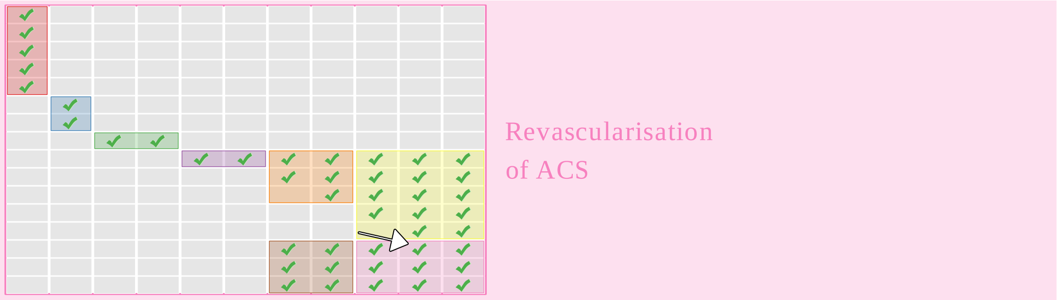

Mixed−effects model for 'unstable angina' and 'PCI'. Incident clinical trigger

	HR	95% CI
Quintile 1	1	(Reference)
Quintile 2	0.83	(0.72; 0.97)
Quintile 3	0.88	(0.76; 1.00)
Quintile 4	0.87	(0.76; 1.00)
Quintile 5	0.76	(0.66; 0.88)
Age 35 to 39	0.81	(0.52; 1.27)
Age 40 to 44	0.53	(0.39; 0.72)
Age 45 to 49	1.00	(0.81; 1.23)
Age 50 to 54	1	(Reference)
Age 55 to 59	0.83	(0.70; 0.99)
Age 60 to 64	0.82	(0.69; 0.97)
Age 65 to 69	0.75	(0.63; 0.89)
Age 70 to 74	0.73	(0.61; 0.87)
Age 75 to 79	0.52	(0.42; 0.63)
Age 80 to 84	0.40	(0.32; 0.50)
Age 85+	0.17	(0.12; 0.24)
Male	1	(Reference)
Female	0.74	(0.67; 0.81)
Non−smoker	1	(Reference)
Smoker	1.16	(1.03; 1.29)
BMI low / norm.	1	(Reference)
Overweight	1.08	(0.96; 1.23)
Obese	0.97	(0.85; 1.11)
No hyp.	1	(Reference)
Hyp. contr.	0.87	(0.78; 0.97)
Hyp. uncontr.	1.03	(0.90; 1.19)
Untreat. hyp.	0.79	(0.59; 1.05)
Chol:HDL < 4	1	(Reference)
Chol:HDL >= 4	1.37	(1.21; 1.54)
No CVA	1	(Reference)
CVA	0.71	(0.61; 0.82)
No oth. co.	1	(Reference)
Other co.	0.72	(0.66; 0.80)
No diabetes	1	(Reference)
Diabetes	0.85	(0.76; 0.95)
Elect. adm.	1	(Reference)
Emer. adm.	0.94	(0.79; 1.13)

	HR	95% CI
Other adm.	1.81	(1.52; 2.17)
Cardiac cen.	1	(Reference)
Other cen.	0.53	(0.32; 0.86)
Cardiology	1	(Reference)
Med. spec.	0.31	(0.28; 0.35)
Other spec.	0.05	(0.04; 0.08)
Indication 1	1	(Reference)
Indication 2	0.96	(0.86; 1.08)
Indication 3	0.87	(0.75; 1.02)
Indication 4	0.72	(0.58; 0.89)
Indication 5+	0.69	(0.55; 0.86)
Indic. years	1.00	(0.99; 1.01)
No prev. acti.	1	(Reference)
1+ prev. acti.	1.40	(1.22; 1.60)

Number of clinical triggers 13907; Number of clinical actions 2130. ICC for practice < 0.005. ICC for hospital = 0.115. Missing values imputed using MICE.

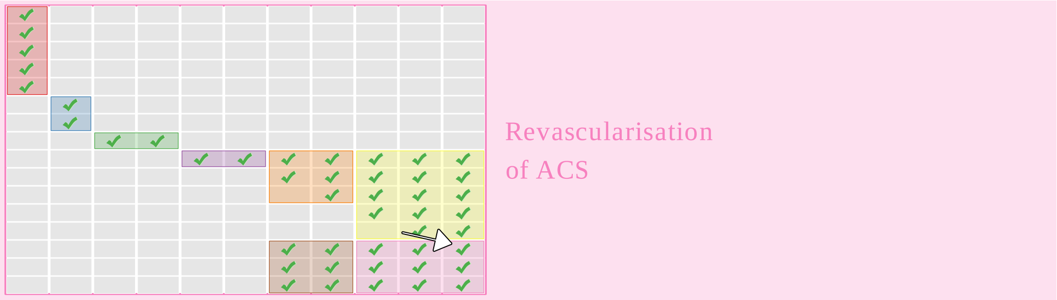

Mixed−effects model for 'MI' and 'PCI'. Incident clinical trigger

	HR	95% CI
Quintile 1	1	(Reference)
Quintile 2	0.89	(0.80; 0.98)
Quintile 3	0.90	(0.82; 0.99)
Quintile 4	0.87	(0.79; 0.95)
Quintile 5	0.83	(0.75; 0.91)
Age 35 to 39	0.77	(0.62; 0.95)
Age 40 to 44	0.90	(0.77; 1.05)
Age 45 to 49	0.90	(0.79; 1.03)
Age 50 to 54	1	(Reference)
Age 55 to 59	0.90	(0.81; 1.01)
Age 60 to 64	0.80	(0.71; 0.89)
Age 65 to 69	0.78	(0.70; 0.87)
Age 70 to 74	0.56	(0.50; 0.63)
Age 75 to 79	0.43	(0.37; 0.48)
Age 80 to 84	0.24	(0.20; 0.28)
Age 85+	0.08	(0.07; 0.10)
Male	1	(Reference)
Female	0.86	(0.80; 0.91)
Non−smoker	1	(Reference)
Smoker	1.15	(1.08; 1.23)
BMI low / norm.	1	(Reference)
Overweight	1.11	(1.01; 1.22)
Obese	1.08	(0.98; 1.18)
No hyp.	1	(Reference)
Hyp. contr.	0.99	(0.92; 1.06)
Hyp. uncontr.	1.04	(0.95; 1.13)
Untreat. hyp.	1.14	(1.00; 1.30)
Chol:HDL < 4	1	(Reference)
Chol:HDL >= 4	1.18	(1.09; 1.28)
No CVA	1	(Reference)
CVA	0.67	(0.59; 0.75)
No oth. co.	1	(Reference)
Other co.	0.70	(0.65; 0.74)
No diabetes	1	(Reference)
Diabetes	0.82	(0.75; 0.88)
Elect. adm.	1	(Reference)
Emer. adm.	1.67	(1.30; 2.13)

	HR	95% CI
Other adm.	1.97	(1.53; 2.53)
Cardiac cen.	1	(Reference)
Other cen.	0.56	(0.34; 0.92)
Cardiology	1	(Reference)
Med. spec.	0.43	(0.40; 0.46)
Other spec.	0.07	(0.05; 0.09)
Indication 1	1	(Reference)
Indication 2	0.84	(0.77; 0.91)
Indication 3	0.68	(0.60; 0.78)
Indication 4	0.67	(0.55; 0.82)
Indication 5+	0.65	(0.50; 0.84)
Indic. years	1.00	(0.99; 1.01)
No prev. acti.	1	(Reference)
1+ prev. acti.	0.93	(0.81; 1.06)

Number of clinical triggers 20467; Number of clinical actions 5118. ICC for practice = 0.013. ICC for hospital = 0.127. Missing values imputed using MICE.

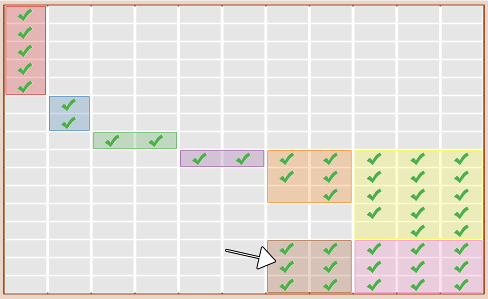

Revascularisation of chronic CHD

Mixed−effects model for 'stable angina' and 'CABG'. Incident clinical trigger

	HR	95% CI
Quintile 1	1	(Reference)
Quintile 2	0.93	(0.77; 1.12)
Quintile 3	0.79	(0.66; 0.96)
Quintile 4	0.78	(0.64; 0.96)
Quintile 5	0.87	(0.71; 1.06)
Age 35 to 39	0.64	(0.26; 1.60)
Age 40 to 44	0.36	(0.17; 0.74)
Age 45 to 49	0.93	(0.62; 1.38)
Age 50 to 54	1	(Reference)
Age 55 to 59	1.22	(0.90; 1.64)
Age 60 to 64	1.45	(1.09; 1.94)
Age 65 to 69	1.70	(1.28; 2.25)
Age 70 to 74	1.68	(1.26; 2.24)
Age 75 to 79	1.57	(1.16; 2.11)
Age 80 to 84	0.78	(0.55; 1.11)
Age 85+	0.19	(0.10; 0.36)
Male	1	(Reference)
Female	0.32	(0.28; 0.37)
Non−smoker	1	(Reference)
Smoker	0.88	(0.75; 1.04)
BMI low / norm.	1	(Reference)
Overweight	0.91	(0.77; 1.08)
Obese	0.71	(0.59; 0.85)
No hyp.	1	(Reference)
Hyp. contr.	1.24	(1.08; 1.43)
Hyp. uncontr.	1.31	(1.10; 1.55)
Untreat. hyp.	1.35	(1.07; 1.69)
Chol:HDL < 4	1	(Reference)
Chol:HDL >= 4	1.49	(1.27; 1.75)
No CVA	1	(Reference)
CVA	0.75	(0.61; 0.93)
No oth. co.	1	(Reference)
Other co.	0.74	(0.64; 0.85)
No prev. acti.	1	(Reference)
1+ prev. acti.	0.00	(0.00; >99)

Number of clinical triggers 18934; Number of clinical actions 1150. ICC for practice = 0.072. Missing values imputed using MICE.

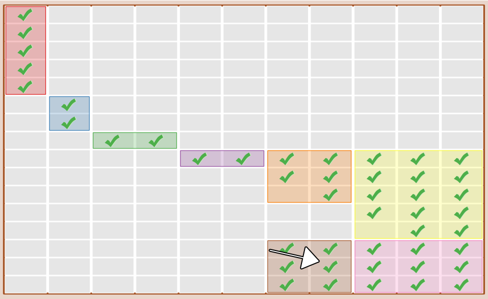

Mixed−effects model for 'stable angina and diabetes' and 'CABG'.
Incident clinical trigger

	HR	95% CI
Quintile 1	1	(Reference)
Quintile 2	0.83	(0.59; 1.18)
Quintile 3	0.96	(0.69; 1.34)
Quintile 4	0.91	(0.65; 1.28)
Quintile 5	0.77	(0.54; 1.09)
Age 35 to 39	0.41	(0.05; 3.10)
Age 40 to 44	0.20	(0.03; 1.51)
Age 45 to 49	0.83	(0.36; 1.89)
Age 50 to 54	1	(Reference)
Age 55 to 59	1.57	(0.93; 2.64)
Age 60 to 64	1.51	(0.91; 2.52)
Age 65 to 69	1.54	(0.93; 2.54)
Age 70 to 74	1.55	(0.94; 2.56)
Age 75 to 79	1.40	(0.83; 2.36)
Age 80 to 84	0.63	(0.33; 1.21)
Age 85+	0.21	(0.07; 0.63)
Male	1	(Reference)
Female	0.44	(0.35; 0.56)
Non−smoker	1	(Reference)
Smoker	0.57	(0.40; 0.81)
BMI low / norm.	1	(Reference)
Overweight	0.90	(0.65; 1.26)
Obese	0.79	(0.57; 1.11)
No hyp.	1	(Reference)
Hyp. contr.	1.20	(0.91; 1.57)
Hyp. uncontr.	1.10	(0.79; 1.52)
Untreat. hyp.	0.85	(0.44; 1.65)
Chol:HDL < 4	1	(Reference)
Chol:HDL >= 4	1.58	(1.22; 2.03)
No CVA	1	(Reference)
CVA	0.87	(0.64; 1.20)
No oth. co.	1	(Reference)
Other co.	0.66	(0.52; 0.83)
Indication 1	1	(Reference)
Indication 2	0.39	(0.27; 0.55)
Indic. years	0.97	(0.93; 1.00)
No prev. acti.	1	(Reference)

	HR	95% CI
1+ prev. acti.	0.00	(0.00; >99)

Number of clinical triggers 8956; Number of clinical actions 385. ICC for practice = 0.138. Missing values imputed using MICE.

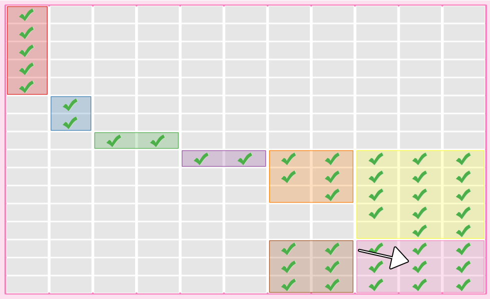

Mixed−effects model for 'unstable angina' and 'CABG'. Incident clinical trigger

	HR	95% CI
Quintile 1	1	(Reference)
Quintile 2	1.02	(0.83; 1.25)
Quintile 3	0.95	(0.78; 1.15)
Quintile 4	1.00	(0.82; 1.22)
Quintile 5	0.97	(0.79; 1.17)
Age 35 to 39	0.52	(0.19; 1.44)
Age 40 to 44	0.54	(0.30; 0.98)
Age 45 to 49	0.48	(0.29; 0.79)
Age 50 to 54	1	(Reference)
Age 55 to 59	1.25	(0.93; 1.68)
Age 60 to 64	1.21	(0.91; 1.61)
Age 65 to 69	1.54	(1.16; 2.04)
Age 70 to 74	1.49	(1.12; 1.97)
Age 75 to 79	1.30	(0.97; 1.74)
Age 80 to 84	0.57	(0.41; 0.81)
Age 85+	0.04	(0.02; 0.09)
Male	1	(Reference)
Female	0.55	(0.48; 0.63)
Non−smoker	1	(Reference)
Smoker	0.77	(0.66; 0.91)
BMI low / norm.	1	(Reference)
Overweight	1.02	(0.85; 1.21)
Obese	0.95	(0.80; 1.14)
No hyp.	1	(Reference)
Hyp. contr.	1.30	(1.11; 1.53)
Hyp. uncontr.	1.63	(1.34; 1.98)
Untreat. hyp.	1.33	(0.93; 1.91)
Chol:HDL < 4	1	(Reference)
Chol:HDL >= 4	1.47	(1.27; 1.69)
No CVA	1	(Reference)
CVA	0.78	(0.64; 0.94)
No oth. co.	1	(Reference)
Other co.	0.60	(0.52; 0.68)
No diabetes	1	(Reference)
Diabetes	1.20	(1.05; 1.37)
Elect. adm.	1	(Reference)
Emer. adm.	1.15	(0.95; 1.39)

	HR	95% CI
Other adm.	1.63	(1.33; 1.99)
Cardiac cen.	1	(Reference)
Other cen.	0.67	(0.43; 1.04)
Cardiology	1	(Reference)
Med. spec.	0.77	(0.65; 0.92)
Other spec.	8.10	(6.94; 9.47)
Indication 1	1	(Reference)
Indication 2	1.37	(1.17; 1.61)
Indication 3	1.55	(1.27; 1.89)
Indication 4	1.30	(0.99; 1.70)
Indication 5+	0.95	(0.69; 1.29)
Indic. years	0.99	(0.98; 1.00)
No prev. acti.	1	(Reference)
1+ prev. acti.	0.03	(0.01; 0.11)

Number of clinical triggers 13907; Number of clinical actions 1155. ICC for practice = 0.031. ICC for hospital = 0.075. Missing values imputed using MICE.

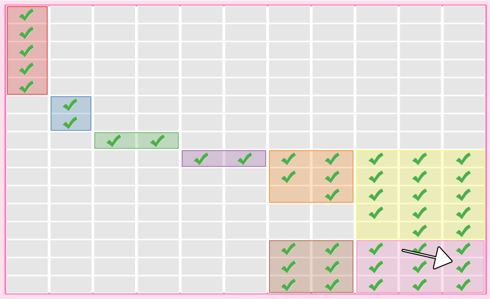

Mixed−effects model for 'MI' and 'CABG'. Incident clinical trigger

	HR	95% CI
Quintile 1	1	(Reference)
Quintile 2	0.98	(0.82; 1.16)
Quintile 3	0.94	(0.80; 1.11)
Quintile 4	0.95	(0.80; 1.13)
Quintile 5	0.89	(0.74; 1.06)
Age 35 to 39	0.33	(0.14; 0.75)
Age 40 to 44	0.70	(0.45; 1.08)
Age 45 to 49	0.80	(0.57; 1.13)
Age 50 to 54	1	(Reference)
Age 55 to 59	1.31	(1.03; 1.67)
Age 60 to 64	1.57	(1.25; 1.97)
Age 65 to 69	1.61	(1.29; 2.03)
Age 70 to 74	1.61	(1.28; 2.02)
Age 75 to 79	1.06	(0.84; 1.36)
Age 80 to 84	0.49	(0.37; 0.65)
Age 85+	0.06	(0.03; 0.10)
Male	1	(Reference)
Female	0.57	(0.50; 0.64)
Non−smoker	1	(Reference)
Smoker	0.85	(0.75; 0.96)
BMI low / norm.	1	(Reference)
Overweight	1.09	(0.95; 1.24)
Obese	1.17	(1.01; 1.35)
No hyp.	1	(Reference)
Hyp. contr.	1.12	(0.98; 1.27)
Hyp. uncontr.	1.31	(1.13; 1.52)
Untreat. hyp.	1.12	(0.87; 1.43)
Chol:HDL < 4	1	(Reference)
Chol:HDL >= 4	1.09	(0.96; 1.25)
No CVA	1	(Reference)
CVA	0.76	(0.64; 0.90)
No oth. co.	1	(Reference)
Other co.	0.59	(0.53; 0.66)
No diabetes	1	(Reference)
Diabetes	1.22	(1.08; 1.37)
Elect. adm.	1	(Reference)
Emer. adm.	2.89	(2.14; 3.89)

	HR	95% CI
Other adm.	3.35	(2.45; 4.59)
Cardiac cen.	1	(Reference)
Other cen.	0.67	(0.46; 0.98)
Cardiology	1	(Reference)
Med. spec.	0.98	(0.86; 1.13)
Other spec.	8.78	(7.64; 10.09)
Indication 1	1	(Reference)
Indication 2	1.28	(1.13; 1.46)
Indication 3	1.36	(1.13; 1.62)
Indication 4	1.37	(1.04; 1.82)
Indication 5+	1.09	(0.76; 1.55)
Indic. years	1.01	(1.00; 1.02)
No prev. acti.	1	(Reference)
1+ prev. acti.	0.04	(0.01; 0.15)

Number of clinical triggers 20467; Number of clinical actions 1645. ICC for practice = 0.05. ICC for hospital = 0.06. Missing values imputed using MICE.

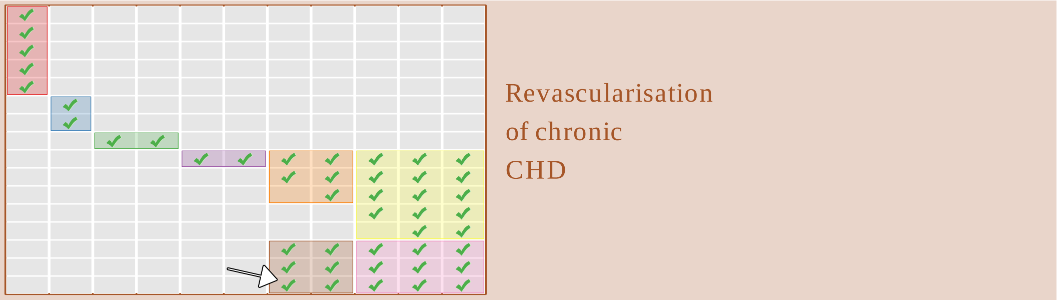

Mixed−effects model for 'stable angina' and 'revascularisation'.
Incident clinical trigger

	HR	95% CI
Quintile 1	1	(Reference)
Quintile 2	0.96	(0.84; 1.10)
Quintile 3	0.86	(0.75; 0.99)
Quintile 4	0.78	(0.68; 0.90)
Quintile 5	0.79	(0.68; 0.92)
Age 35 to 39	0.90	(0.56; 1.46)
Age 40 to 44	0.91	(0.67; 1.23)
Age 45 to 49	1.12	(0.89; 1.41)
Age 50 to 54	1	(Reference)
Age 55 to 59	1.07	(0.89; 1.29)
Age 60 to 64	1.18	(0.99; 1.41)
Age 65 to 69	1.15	(0.96; 1.38)
Age 70 to 74	1.08	(0.90; 1.30)
Age 75 to 79	0.79	(0.64; 0.96)
Age 80 to 84	0.42	(0.33; 0.55)
Age 85+	0.11	(0.07; 0.18)
Male	1	(Reference)
Female	0.43	(0.39; 0.47)
Non−smoker	1	(Reference)
Smoker	0.93	(0.83; 1.04)
BMI low / norm.	1	(Reference)
Overweight	0.98	(0.88; 1.09)
Obese	0.77	(0.67; 0.89)
No hyp.	1	(Reference)
Hyp. contr.	1.11	(1.01; 1.23)
Hyp. uncontr.	1.29	(1.14; 1.45)
Untreat. hyp.	1.34	(1.14; 1.57)
Chol:HDL < 4	1	(Reference)
Chol:HDL >= 4	1.57	(1.42; 1.74)
No CVA	1	(Reference)
CVA	0.65	(0.54; 0.77)
No oth. co.	1	(Reference)
Other co.	0.67	(0.60; 0.74)
No prev. acti.	1	(Reference)
1+ prev. acti.	0.00	(0.00; >99)

Number of clinical triggers 18934; Number of clinical actions 2298. ICC for practice = 0.074. Missing values imputed using MICE.

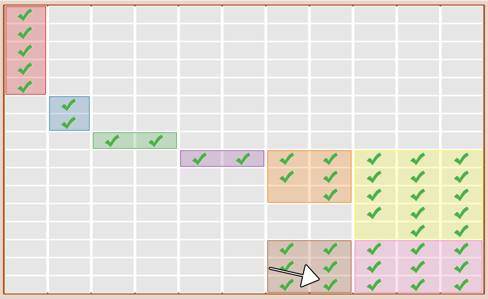

Revascularisation of chronic CHD

Mixed−effects model for 'stable angina and diabetes' and 'revascularisation'. Incident clinical trigger

	HR	95% CI
Quintile 1	1	(Reference)
Quintile 2	0.75	(0.57; 0.98)
Quintile 3	0.88	(0.68; 1.13)
Quintile 4	0.81	(0.62; 1.04)
Quintile 5	0.79	(0.61; 1.03)
Age 35 to 39	0.83	(0.33; 2.10)
Age 40 to 44	0.87	(0.45; 1.68)
Age 45 to 49	1.12	(0.70; 1.80)
Age 50 to 54	1	(Reference)
Age 55 to 59	1.13	(0.80; 1.58)
Age 60 to 64	1.00	(0.72; 1.40)
Age 65 to 69	0.96	(0.69; 1.33)
Age 70 to 74	0.95	(0.68; 1.33)
Age 75 to 79	0.68	(0.47; 0.97)
Age 80 to 84	0.29	(0.17; 0.48)
Age 85+	0.16	(0.07; 0.34)
Male	1	(Reference)
Female	0.54	(0.45; 0.64)
Non−smoker	1	(Reference)
Smoker	0.70	(0.55; 0.88)
BMI low / norm.	1	(Reference)
Overweight	1.12	(0.86; 1.46)
Obese	1.02	(0.78; 1.32)
No hyp.	1	(Reference)
Hyp. contr.	1.16	(0.95; 1.42)
Hyp. uncontr.	1.10	(0.86; 1.41)
Untreat. hyp.	1.13	(0.72; 1.76)
Chol:HDL < 4	1	(Reference)
Chol:HDL >= 4	1.30	(1.11; 1.53)
No CVA	1	(Reference)
CVA	0.87	(0.68; 1.11)
No oth. co.	1	(Reference)
Other co.	0.65	(0.54; 0.78)
Indication 1	1	(Reference)
Indication 2	0.39	(0.30; 0.51)
Indic. years	0.96	(0.93; 0.99)
No prev. acti.	1	(Reference)

	HR	95% CI
1+ prev. acti.	0.53	(0.27; 1.04)

Number of clinical triggers 8956; Number of clinical actions 676. ICC for practice = 0.104. Missing values imputed using MICE.

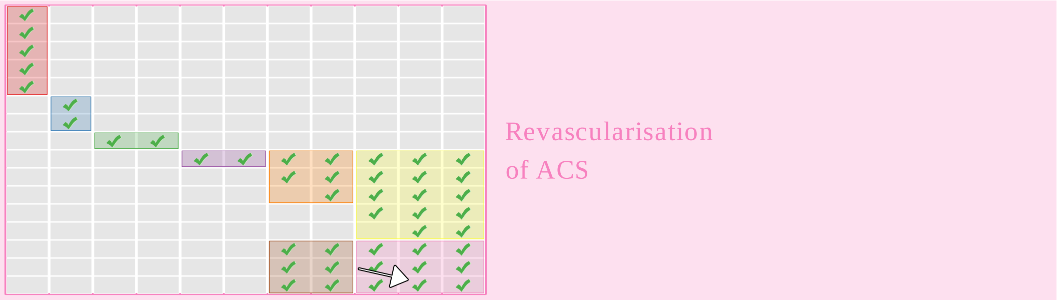

Mixed−effects model for 'unstable angina' and 'revascularisation'.
Incident clinical trigger

	HR	95% CI
Quintile 1	1	(Reference)
Quintile 2	0.90	(0.80; 1.02)
Quintile 3	0.92	(0.82; 1.04)
Quintile 4	0.92	(0.82; 1.04)
Quintile 5	0.83	(0.74; 0.94)
Age 35 to 39	0.70	(0.47; 1.06)
Age 40 to 44	0.53	(0.40; 0.70)
Age 45 to 49	0.89	(0.73; 1.08)
Age 50 to 54	1	(Reference)
Age 55 to 59	0.93	(0.80; 1.08)
Age 60 to 64	0.90	(0.77; 1.04)
Age 65 to 69	0.93	(0.80; 1.08)
Age 70 to 74	0.88	(0.76; 1.02)
Age 75 to 79	0.69	(0.59; 0.81)
Age 80 to 84	0.41	(0.34; 0.49)
Age 85+	0.11	(0.08; 0.15)
Male	1	(Reference)
Female	0.64	(0.59; 0.70)
Non−smoker	1	(Reference)
Smoker	1.01	(0.92; 1.11)
BMI low / norm.	1	(Reference)
Overweight	1.09	(0.99; 1.21)
Obese	0.96	(0.85; 1.08)
No hyp.	1	(Reference)
Hyp. contr.	0.98	(0.90; 1.07)
Hyp. uncontr.	1.23	(1.10; 1.38)
Untreat. hyp.	0.93	(0.74; 1.16)
Chol:HDL < 4	1	(Reference)
Chol:HDL >= 4	1.41	(1.25; 1.60)
No CVA	1	(Reference)
CVA	0.70	(0.62; 0.79)
No oth. co.	1	(Reference)
Other co.	0.66	(0.61; 0.71)
No diabetes	1	(Reference)
Diabetes	0.96	(0.88; 1.04)
Elect. adm.	1	(Reference)
Emer. adm.	1.01	(0.89; 1.15)

	HR	95% CI
Other adm.	1.88	(1.64; 2.15)
Cardiac cen.	1	(Reference)
Other cen.	0.49	(0.31; 0.77)
Cardiology	1	(Reference)
Med. spec.	0.36	(0.33; 0.40)
Other spec.	1.17	(1.04; 1.31)
Indication 1	1	(Reference)
Indication 2	1.14	(1.04; 1.25)
Indication 3	1.19	(1.06; 1.35)
Indication 4	1.02	(0.86; 1.21)
Indication 5+	0.86	(0.71; 1.03)
Indic. years	0.99	(0.99; 1.00)
No prev. acti.	1	(Reference)
1+ prev. acti.	0.73	(0.65; 0.82)

Number of clinical triggers 13907; Number of clinical actions 3230. ICC for practice = 0.01. ICC for hospital = 0.104. Missing values imputed using MICE.

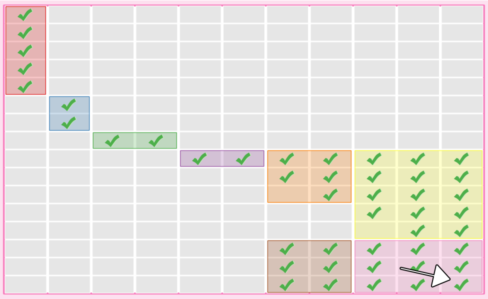

Mixed−effects model for 'MI' and 'revascularisation'. Incident clinical trigger

	HR	95% CI
Quintile 1	1	(Reference)
Quintile 2	0.89	(0.82; 0.97)
Quintile 3	0.90	(0.83; 0.97)
Quintile 4	0.87	(0.80; 0.95)
Quintile 5	0.83	(0.77; 0.91)
Age 35 to 39	0.68	(0.55; 0.83)
Age 40 to 44	0.87	(0.75; 1.01)
Age 45 to 49	0.89	(0.78; 1.00)
Age 50 to 54	1	(Reference)
Age 55 to 59	0.97	(0.87; 1.07)
Age 60 to 64	0.89	(0.81; 0.98)
Age 65 to 69	0.89	(0.80; 0.98)
Age 70 to 74	0.69	(0.62; 0.76)
Age 75 to 79	0.49	(0.44; 0.55)
Age 80 to 84	0.25	(0.22; 0.28)
Age 85+	0.07	(0.05; 0.08)
Male	1	(Reference)
Female	0.74	(0.70; 0.78)
Non−smoker	1	(Reference)
Smoker	1.10	(1.04; 1.16)
BMI low / norm.	1	(Reference)
Overweight	1.13	(1.06; 1.21)
Obese	1.10	(1.02; 1.19)
No hyp.	1	(Reference)
Hyp. contr.	1.04	(0.97; 1.10)
Hyp. uncontr.	1.12	(1.04; 1.21)
Untreat. hyp.	1.12	(1.00; 1.26)
Chol:HDL < 4	1	(Reference)
Chol:HDL >= 4	1.17	(1.10; 1.24)
No CVA	1	(Reference)
CVA	0.67	(0.61; 0.74)
No oth. co.	1	(Reference)
Other co.	0.65	(0.61; 0.69)
No diabetes	1	(Reference)
Diabetes	0.90	(0.84; 0.96)
Elect. adm.	1	(Reference)
Emer. adm.	2.14	(1.76; 2.60)

	HR	95% CI
Other adm.	2.73	(2.24; 3.34)
Cardiac cen.	1	(Reference)
Other cen.	0.51	(0.30; 0.87)
Cardiology	1	(Reference)
Med. spec.	0.47	(0.44; 0.50)
Other spec.	0.95	(0.85; 1.06)
Indication 1	1	(Reference)
Indication 2	0.99	(0.92; 1.06)
Indication 3	0.92	(0.83; 1.03)
Indication 4	0.94	(0.80; 1.12)
Indication 5+	0.92	(0.75; 1.14)
Indic. years	1.00	(0.99; 1.01)
No prev. acti.	1	(Reference)
1+ prev. acti.	0.55	(0.49; 0.62)

Number of clinical triggers 20467; Number of clinical actions 6649. ICC for practice = 0.007. ICC for hospital = 0.142. Missing values imputed using MICE.
